# Synthesis, Molecular Electron Density Theory Study, Molecular Docking, and Pharmacological Evaluation of New Coumarin–Sulfonamide–Nitroindazolyl–Triazole Hybrids as Monoamine Oxidase Inhibitors

**DOI:** 10.3390/ijms25126803

**Published:** 2024-06-20

**Authors:** Mohammed Eddahmi, Gabriella La Spada, Luis R. Domingo, Gérard Vergoten, Christian Bailly, Marco Catto, Latifa Bouissane

**Affiliations:** 1 Molecular Chemistry, Materials and Catalysis Laboratory, Faculty of Sciences and Technologies, Sultan Moulay Slimane University, BP 523, Beni-Mellal 23000, Morocco; eddahmi.med@gmail.com; 2 Department of Pharmacy-Pharmaceutical Sciences, University of Bari Aldo Moro, Via E. Orabona 4, 70125 Bari, Italy; gabriella.laspada@uniba.it (G.L.S.); marco.catto@uniba.it (M.C.); 3 Department of Organic Chemistry, University of Valencia, Dr. Moliner 50, 46100 Burjassot, Valencia, Spain; luisrdomingo@gmail.com; 4 Institute of Pharmaceutical Chemistry Albert Lespagnol (ICPAL), Faculty of Pharmacy, University of Lille, Rue du Professeur Laguesse, BP-83, F-59006 Lille, France

**Keywords:** coumarin–sulfonamide–nitroindazolyl–triazole hybrids, MEDT, electrophilic aromatic substitution, AS, AChE inhibitors, MAO inhibitors, nitrocoumarin, CuAAC

## Abstract

Inhibitors of monoamine oxidases (MAOs) are of interest for the treatment of neurodegenerative disorders and other human pathologies. In this frame, the present work describes different synthetic strategies to obtain MAO inhibitors via the coupling of the aminocoumarin core with arylsulfonyl chlorides followed by copper azide-alkyne cycloaddition, leading to coumarin–sulfonamide–nitroindazolyl–triazole hybrids. The nitration position on the coumarin moiety was confirmed through nuclear magnetic resonance spectroscopy and molecular electron density theory in order to elucidate the molecular mechanism and selectivity of the electrophilic aromatic substitution reaction. The coumarin derivatives were evaluated for their inhibitory potency against monoamine oxidases and cholinesterases. Molecular docking calculations provided a rational binding mode of the best compounds in the series with MAO A and B. The work identified hybrids **14a**–**c** as novel MAO inhibitors, with a selective action against isoform B, of potential interest to combat neurological diseases.

## 1. Introduction

Coumarin-based hybrid molecules present several biological interests. They play an important role in the development of new drug therapies [1,2,3,4,5,6,7]. Considering their relevance, several studies have described their reactivity and applications [8,9,10,11,12,13,14,15,16,17,18,19,20,21,22,23,24]. Heterocycles bearing sulfonamide moieties have attracted attention as well, thanks to their significant biological properties [25,26,27,28,29,30]. In most cases, *N*-arylsulfonamides were synthesized through the reduction of the nitro group and the sulfonylation of the corresponding amines with sulfonyl chloride in the presence of a base [31,32,33]. The synthesis of sulfonamide derivatives via a one-pot reductive and coupling reaction between arylsulfonyl chlorides and nitroarenes in the presence of Fe dust in an aqueous medium has recently been reported [34]. An efficient method for the construction of *N*-aryl sulfonamides starting from sodium arylsulfinates and nitroarenes in the presence of FeCl_2_ and NaHSO_3_ was described by Luo’s group [35]. More recently, a similar procedure of cross-coupling sodium arylsulfinates with the nitro group of nitroarenes was carried out in the presence of a Pd/C catalyst, leading to functionalized *N*-aryl sulfonamides [36].

Based on the previous studies describing the inhibition of monoamine oxidase and cholinesterase exerted by indazoles [37], 1,2,3-triazole–coumarin hybrids [38,39,40], and arylsulfonamides [41,42], this research aims to contribute to the development of novel molecule conjugates by exploring the linkage of a coumarin bearing a nitro group to sulfonamide and indazole, through simple and efficient synthetic routes, such as reduction, coupling reactions, and a copper-azide-alkyne-cycloaddition (CuAAC) methodology in order to prepare and evaluate the potential of new hybrids as neuroprotective agents. The drug design derives from previous studies with coumarins and related compounds (e.g., benzodioxine), which have revealed marked anti-inflammatory properties [43,44]. Here, we present the nitration condition of coumarin and its position confirmation through a relevant study of the molecular mechanism and regioselectivity in the electrophilic aromatic substitution reaction, which was analyzed within the framework of the Molecular Electron Density Theory (MEDT) [45]. In addition, we reported the synthesis of the new conjugated compounds through a series of effective pathways, their molecular docking study, and their capacity to inhibit monoamine oxidases.

## 2. Results

### 2.1. Nitration of Coumarin

**Scheme 1:**
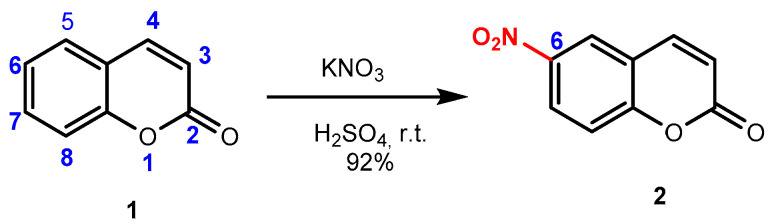
Nitration of 2*H*-chromen-2-one **1**.

The nitration of commercial coumarin (1) was carried out using an equimolar amount of potassium nitrate in sulfuric acid solution (Scheme 1). After 24 h of stirring at room temperature, 6-nitro-2*H*-chromen-2-one 2 was obtained in high yield and its structure was confirmed using NMR spectroscopy and high-resolution mass spectrometry (Figures S1–S4).

### 2.2. MEDT Study of the Nitration Reaction of Coumarin in Sulfuric Acid

**Scheme 2:**
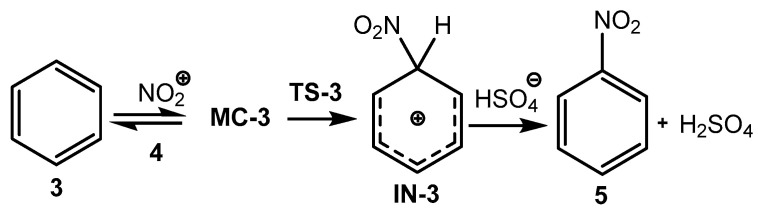
Mechanism of the EAS nitration reaction of benzene **3** with the nitronium NO_2_^+^ ion **4**.

Recent MEDT studies of the electrophilic aromatic substitution (EAS) nitration reactions of benzene and deactivated aromatic compounds have shown that these EAS reactions take place through a stepwise mechanism involving the formation of a tetrahedral cationic intermediate IN (Scheme 2) [46,47].

This EAS nitration reaction begins with the electrophilic attack of the nitronium NO_2_^+^ ion **4** on one of the six carbons of benzene **3**, yielding, via transition state **TS-3**, the tetrahedral cation intermediate **IN-3**. During the approach of the nitronium NO_2_^+^ ion **4** to benzene **3**, a weak molecular complex **MC-3**, resulting from weak electronic interactions between the strong electrophilic nitronium NO_2_^+^ ion **4** and the aromatic electron density of benzene **3**, is found on the potential energy surface (PES). The formation of **MC-3** is exothermic, but endergonic. Although the formation of **IN-3** is exergonic, the subsequent proton abstraction does not have any appreciable barrier. The strong exergonic character of this EAS reaction makes it irreversible. Consequently, the EAS nitration reaction of benzene **3** is kinetically controlled [46,47].

**Chart 1:**
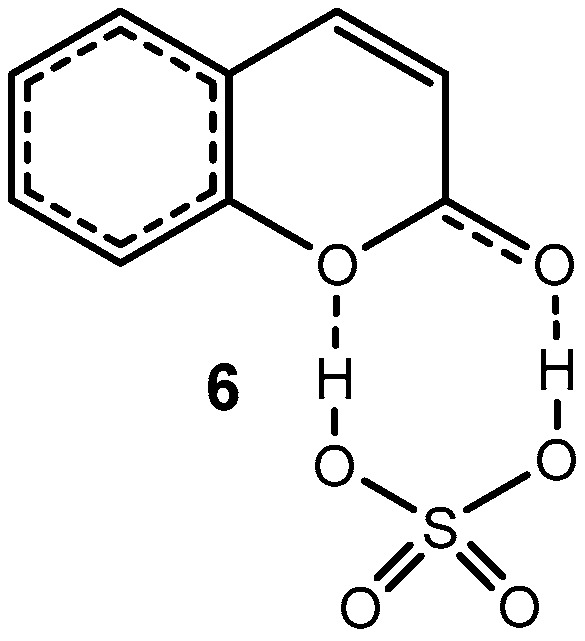
Coumarin: SO_4_H_2_ complex **6** involved in the EAS nitration reaction of coumarin **1**.

Due to the presence of two basic oxygen atoms in coumarin **1**, it is expected that in presence of sulfuric acid, they will form two hydrogen bonds (HBs) with the two hydrogens of sulfuric acid, yielding the coumarin: SO_4_H_2_ complex **6** (Chart 1). Consequently, this species has been considered as the substrate that experiences the EAS nitration reaction of coumarin **1** in this MEDT study.

#### 2.2.1. Analysis of the CDFT Reactivity Indices

The conceptual DFT [48,49] (CDFT) reactivity indices are a useful tool to analyze the molecular reactivity and the regioselectivity in polar processes [50,51]. The CDFT indices were calculated at the B3LYP/6-31G(d) computational level, since it was used to define the electrophilicity and nucleophilicity scales [49]. The B3LYP/6-31G(d) global indices, namely the electronic chemical potential, µ; chemical hardness, ɳ; global electrophilicity, ω; and global nucleophilicity, *N* of coumarin **1**, coumarin: SO_4_H_2_ complex **6**, and nitronium NO_2_^+^ ion **4**, are given in Table 1.

The electronic chemical potential [52], µ, of coumarin **1**—μ = −4.19 eV—and the coumarin: SO_4_H_2_ complex **6**—μ = −5.09 eV—are higher than that of the nitronium NO_2_^+^ ion 4—μ = −16.18 eV—suggesting that along the polar EAS reaction, the global electron density transfer [53] (GEDT) will take place from coumarin **1** and the coumarin: SO_4_H_2_ complex **6** towards the nitronium NO_2_^+^ ion **4**. According to Table 1, the electrophilicity [54], ω, and nucleophilicity [55], *N*, indices of coumarin **1** are 1.90 and 2.62 eV, respectively. These values permit its classification as a strong electrophile and a moderate nucleophile within the electrophilic and nucleophilic scales [45]. On the other hand, the coumarin: SO_4_H_2_ complex **6**, with electrophilicity, ω, and nucleophilicity, *N*, indices of 2.81 eV and 1.72 eV, respectively, is classified as a strong electrophile and as a marginal nucleophile. The formation of the two HBs between sulfuric acid and the two oxygen of coumarin **1** increases the electrophilicity and decreases the nucleophilicity of the coumarin: SO_4_H_2_ complex **6**. Consequently, it will be less reactive as a nucleophile than coumarin **1**.

Nitronium NO_2_^+^ ion **4** has electrophilicity, ω, and nucleophilicity, *N*, indices of 14.01 and −11.70 eV, respectively, being classified as a strong electrophile and an extremely marginal nucleophile. The very high electrophilicity, ω, value of nitronium NO_2_^+^ ion **4**, higher than 3.0 eV, permits its classification as a superelectrophile [50]. These species are able to react even with marginal nucleophiles such as the coumarin: SO_4_H_2_ complex **6**. Consequently, although coumarin **1** and coumarin: SO_4_H_2_ complex **6** are classified as moderate and marginal nucleophiles, respectively, the superelectrophilic character of nitronium NO_2_^+^ ion **4** makes the former molecules react as nucleophiles in polar processes [47].

#### 2.2.2. Analysis of the Electronic Structure of Coumarin **1** and Coumarin: SO_4_H_2_ Complex **6**

**Figure 1:**
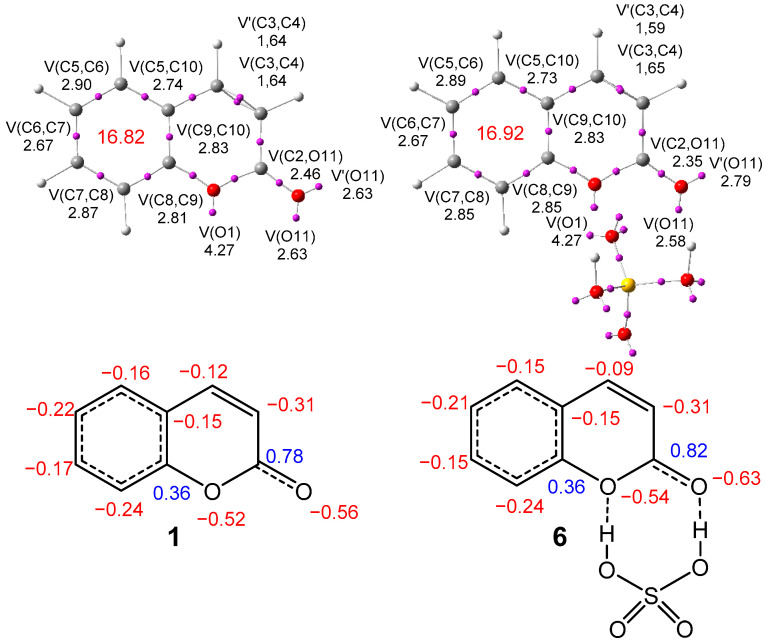
*ω*B97X-D/6-311G(d,p) ELF basin attractor positions, together with the valence basin populations of coumarin **1** and coumarin: SO_4_H_2_ complex **6**. Negative charges are colored in red, and positive charges in blue. ELF valence basin populations and natural atomic charges are given in average number of electrons, e.

The topological analysis of the electron localization function [56] (ELF) at the ground state of the reagents allows for a quantitative and qualitative description of the electronic structure of organic molecules [57]. In order to understand how the HB formation in coumarin **1** modifies its electronic structure and, consequently, its reactivity, a comparative ELF topological analysis of coumarin **1** and coumarin: SO_4_H_2_ complex **6** was performed. ELF basin attractor positions together with the valence basin populations are shown in Figure 1.

The topological analysis of the ELF of ring A of coumarin **1** shows the presence of two disynaptic basins integrating 1.61 e (V(O1,C10)) and 1.67 e (V(O1,C2)); two disynaptic basins integrating 2.40e (V(C2,C3)) and 2.30 e (V(C4,C5)); one V(C2,O11) disynaptic basin integrating 2.46 e; two disynaptic basins, V(C3,C4) and V(C3,C4), integrating a total of 3.28 e; one V(O1) monosynaptic basin integrating 4.72 e; and two monosynaptic basins, V(O11) and V’(O11), integrating a total of 5.26 e, characterizing the non-bonding electron density regions of the two oxygen atoms. The very low population of the V(C2,O11) disynaptic basin, which is associated with the carboxyl C2–O11 bonding region, points to a very depopulated C=O double bond.

Ring B of coumarin **1** shows the presence of six V(Ci,Cj) disynaptic basins, integrating between 2.67 and 2.90 e, showing some polarization of the ring electron density [58] (RED) of this aromatic ring (Figure 1). The RED of this aromatic ring, 16.82 e, is slightly higher than that of benzene, at 16.56 e [58].

The topological analysis of the ELF of coumarin: SO_4_H_2_ complex **6** shows non-remarked changes with respect to the ELF of coumarin **1**. The formation of the two HBs mainly polarizes the carbonyl C2–O11 bonding region towards the oxygen atom, decreasing the population of the V(C2,O11) disynaptic basing by 0.11 e, and increasing the population of the two V(O11) and V’(O11) monosynaptic basins by 0.11 e. The conjugated C3–C4 double bond is also slightly depopulated by 0.04 e. Unappreciable changes are observed in the aromatic region of coumarin **1**. The RED of coumarin: SO_4_H_2_ complex **6** is increased by 0.10 e (Figure 1). Finally, the topological analysis of the ELF of nitronium NO_2_^+^ ion **4** shows the presence of two V(N,O) disynaptic basins, each one integrating 3.04 e, and two V(O) nonosinaptic basins, each one integrating 4.74 e.

The natural population analysis [59,60] (NPA) of coumarin **1** shows a strong polarization of the bonding regions around the two oxygen atoms; thus, the C2 and C9 carbons, bonded to the two oxygen atoms, are positively charged by 0.78 and 0.36 e, respectively. Interestingly, the aromatic C9 carbon is positively charged. This behavior implies that the C8–C5 carbons belonging to the aromatic ring will be alternatively charged, with C6, at −0.22 e, being one of the aromatic carbons that is more negatively charged. The formation of the HBs markedly polarizes the carbonyl C2–O11 bonding region of 6, while the carbons of the aromatic ring are practically non-changed (Figure 1).

#### 2.2.3. Study of the Reaction Mechanism and Regioselectivity Associated to the EAS Nitration Reaction of Coumarin: SO_4_H_2_ complex **6** with Nitronium NO_2_^+^ ion **4**

**Scheme 3:**
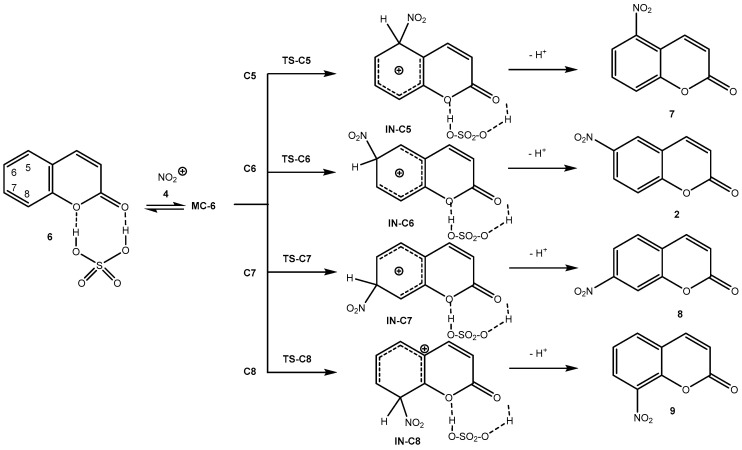
Competitive regioisomeric reaction paths associated with the EAS nitration reaction of coumarin: SO_4_H_2_ complex **6** with nitronium NO_2_^+^ ion **4**.

Four completive regioisomeric reaction paths, named C5–C8, for the EAS nitration reaction of coumarin: SO_4_H_2_ complex **6** with nitronium NO_2_^+^ ion **4** have been analyzed (Scheme 3). The exploration of the PES shows that this EAS reaction takes place through a stepwise mechanism, similar to that of benzene **3** in Scheme 1. Therefore, four transition state structures, **TS-CX** (X = 5–8), were characterized. The thermodynamic data are given in Table 2.

The formation of **MC-6** is exothermic by 0.40 kcal·mol^−1^, and endergonic by 6.25 kcal·mol^−1^. Consequently, the formation of **MC-6** does not have any relevance in this EAS reaction. The activation Gibbs free energies associated with the electrophilic attack of nitronium NO_2_^+^ ion **4** on the four aromatic positions of the ring A of coumarin **1** are 14.57 (**TS-C5**), 12.97 (**TS-C6**), 15.97 (**TS-C7**), and 16.20 (**TS-C8**) kcal·mol^−1^; the formation of the corresponding tetrahedral cation intermediate, INs, is endergonic between 1.68 (**IN-C6**) and 9.59 (**IN-C5**) kcal·mol^−1^.

The subsequent deprotonations of these cation intermediates convert them into the final nitro-derivatives 2, 7–9 without any appreciable barrier [46,47], the overall EAS nitration reaction being strongly exergonic by more than 103 kcal·mol^−1^. Consequently, this EAS nitration reaction is kinetically controlled.

**Table 2:** Values of *ω*B97X-D/6-311G(d,p) relative enthalpies. SCRF (self-consistent reaction field) values include ΔH, entropies ΔS, and Gibbs free energies ΔG (in kcal·mol^−1^). Data were computed at 25 °C and 1 atm, in water, for the stationary points involved in the EAS nitration reaction of coumarin: SO_4_H_2_ complex **6** with nitronium NO_2_^+^ ion **4**.

	ΔH	ΔS	ΔG
**MC-6**	−0.40	−22.30	6.25
**TS-C5**	4.36	−34.24	14.57
**TS-C6**	2.81	−34.08	12.97
**TS-C7**	5.51	−35.10	15.97
**TS-C8**	4.30	−39.94	16.20
**IN-C5**	−1.33	−36.57	9.58
**IN-C6**	−8.50	−34.14	1.68
**IN-C7**	−1.88	−33.35	8.06
**IN-C8**	−8.19	−38.55	3.30
**7**	−104.64	10.73	−107.84
**2**	−99.76	10.57	−102.91
**8**	−100.76	8.90	−103.41
**9**	−101.93	14.73	−106.32

Taking into account that this EAS nitration reaction takes place through a favorable kinetic control, the Eyring–Polanyi relation [61] given in Equation (1) was used to estimate the composition of the reaction mixture.

(1) $$k=\frac{{kk}_{B}T}{h}e^{-\frac{{\Delta G}^{‡}}{RT}}$$

From this equation, the relative reaction rate constants *k_rel_* can be obtained as follows:

(2) $$k_{rel}=e^{-\frac{{\Delta \Delta G}^{‡}}{RT}}$$

were ${\Delta \Delta G}^{‡}$ is the relative activation Gibbs free energy of the four transition states (TSs), *R* the ideal gas constant, and *T* is the reaction temperature.

Considering the Gibbs free energies associated with the four TSs given in Table 2 and the reaction temperature (25 °C), the following relationship between the four feasible isomeric nitro-derivatives can be estimated: 6.25 (**7**): 92.75 (**2**): 0.59 (**8**): 0.40 (**9**). This result indicates that nitro-derivative **2**, resulting from the electrophilic attack to the C6 position of coumarin **1**, is expected to be the majority isomer, at ca. 93%.

**Figure 2:**
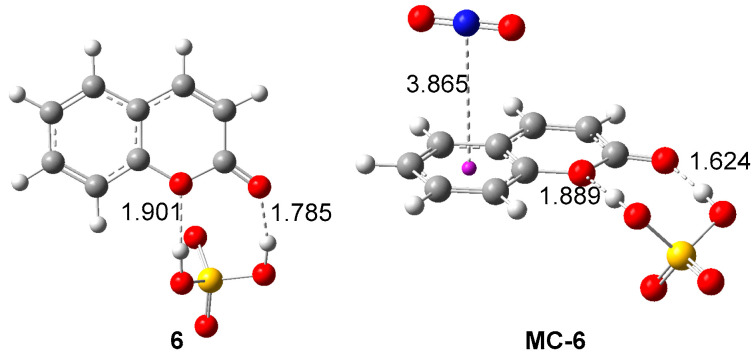
*ω*B97X-D/6-311G(d,p) geometries of coumarin: SO_4_H_2_ complex **6** and **MC-6**. Distance is given in Angstroms (Å).

**Figure 3:**
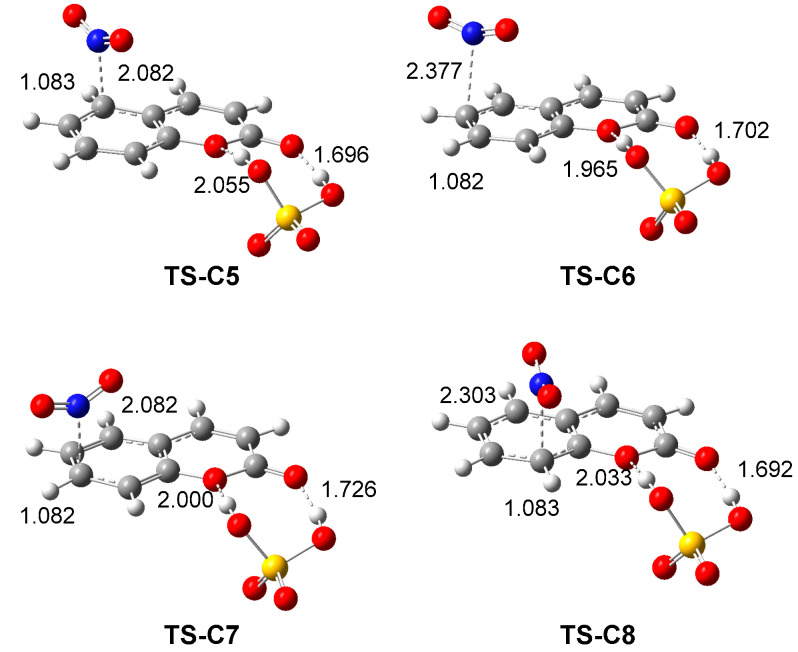
*ω*B97X-D/6-311G(d,p) geometries of the TSs involved in the EAS nitration reaction of coumarin: SO_4_H_2_ complex **6** with nitronium NO_2^+^_ ion **4**. Distances are given in Angstroms (Å).

The geometries of coumarin: SO_4_H_2_ complex **6** and **MC-6** are given in Figure 2, while the geometries of the four TSs are given in Figure 3. For coumarin: SO_4_H_2_ complex **6**, the distances between the O1 and O11 oxygen atoms of coumarin and the two hydrogens of sulfuric acid are 1.901 and 1.786 Å, respectively. These short distances point to strong HB interactions at this species, notably involving the carboxyl O11 oxygen. At **MC-6**, the nitronium NO_2_^+^ ion **4** is positioned over the aromatic plain of coumarin **1** at a distance of 3.86 Å. The two H–O distances associated with the two HBs are slightly reduced at **MC-6**. The HB involving the carboxyl O11 oxygen experiences a higher reduction, at 0.14 Å.

At the four TSs, the N–C distances between the nitrogen atom of nitronium NO_2_^+^ ion **4** and the interacting aromatic carbon are 2.082 (**TS-C5**), 2.377 (**TS-C6**), 2.082 (**TS-C7**), and 2.303 (**TS-C8**) Å, while the lengths of the corresponding H–C single bonds are ca. 1.08 Å. The most favorable, **TS-C6**, presents a larger N–C distance, indicating that this is the earlier TS. Interestingly, at the four TSs, while the HB distance involving the O1 oxygen is slightly increased, that involving the carboxyl O11 oxygen is slightly reduced.

The GEDT [53] at the four TSs, which fluxes from the aromatic ring of coumarin **1** to nitronium NO_2_^+^ ion **4** are 0.63 e at **TS-C5**, 0.36 e at **TS-C6**, 0.63 e at **TS-C7**, and 0.40 e at **TS-C8**. Along the reaction paths, the GEDT increases with the reduction in the N–C distance, reaching its maximum value at the corresponding cation intermediate; thus, at **IN-C6**, the GEDT is 1.14 e. Consequently, the most favorable **TS-C6**, which is the earlier TS, presents the lowest GEDT value, 0.36 e.

**Figure 4:**
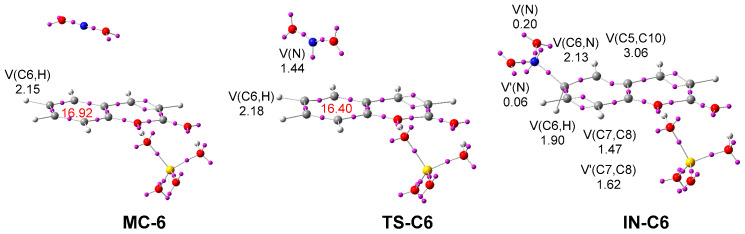
*ω*B97X-D/6-311G(d,p) ELF basin attractor positions, together with the valence basin populations of **MC-6**, **TS-C6**, and **IN-C6**. The RED is given in red. The populations of the most relevant valence basins are given in electrons (e).

Finally, a topological analysis of the ELF of the stationary points involved along the most favorable reaction path was performed. ELF attractor positions of **MC-6**, **TS-C6**, and **IN-C6** are shown in Figure 4. The ELF of **MC-6** is close to that of the separated reagents (Figure 1). The ELF of **TS-C6** shows the creation of one V(N) monosynaptic basin, integrating 1.44 e. A part of the electron density of this monosynaptic basin comes from the GEDT taking place along this polar reaction, 0.36 e. Note that the RED at **TS-6C** has been reduced by 0.50 e with respect to that of **MC-6**.

The most relevant changes are found at the tetrahedral cation intermediate **IN-C6**. A new V(C6,N) disynaptic basin, integrating 2.13 e, is created, indicating that the new **C6-N** single bond has already been formed. On the other hand, the V(C6,H) disynaptic basin has been depopulated by only 0.15 e with respect to that in **MC-6**. These two disynaptic basins characterize the tetrahedral nature of the C6 carbon of this cation intermediate. Interestingly, the C7–C8 bonding region is characterized by the presence of two disynaptic basins, V(C7,C8) and V’(C7,C8), integrating a total of 3.09 e. Note that for coumarin: SO_4_H_2_ complex **6**, the V(C7,C8) disynaptic basin integrates 2.85 e (Figure 1).

Altogether, the MEDT study characterizes the reaction pathway for the nitration of coumarin **1**. Due to the presence of two basic oxygens in coumarin **1**, a coumarin: SO_4_H_2_ complex **6** is formed earlier in the sulfuric acid solution, which undergoes the SEA nitration reaction with the NO_2_^+^ ion **4**. This reaction proceeds via a two-step mechanism with the formation of a tetrahedral cation intermediate **IN-C6**, via the most favorable regioisomeric **TS-C6**. This intermediate without an appreciable barrier experiences a proton elimination to irreversibly give the experimental 6-nitro-2*H*-chromen-2-one **2**. The preferential electrophilic attack of NO_2_^+^ ion **4** on the C6 carbon of the aromatic coumarin ring via **TS-C6** determines the regioselectivity in this SEA reaction. The most favorable **TS-C6** is the earliest, according to both geometric and ELF topological analysis of the four competing TSs. Although the formation of coumarin: SO_4_H_2_ complex **6** significantly reduces the nucleophilic character of coumarin **1**, the superelectrophilic character of the NO_2_^+^ ion **4** justifies the low activation enthalpy associated with the nitration reaction.

### 2.3. Synthesis of N-(2-oxo-2H-Chromen-6-yl)benzenesulfonamides

**Scheme 4:**
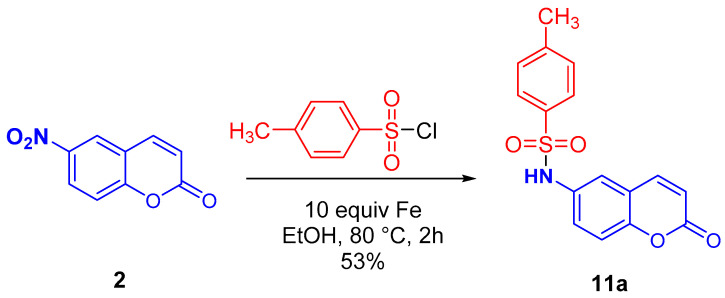
Synthesis of **11a** in one-pot reduction.

To link the coumarin moiety with arylsulfonyl, we treated 6-nitro-2*H*-chromen-2-one **2** with 4-methylbenzenesulfonyl chloride in a one-pot reaction. The reduction of the nitro group was carried out in the presence of Fe dust with ethanol reflux followed by the coupling reaction, leading to the corresponding 4-methyl-N-(2-oxo-2*H*-chromen-6-yl)benzenesulfonamide **11a** with a reasonably good yield of 53% (Scheme 4).

**Scheme 5:**
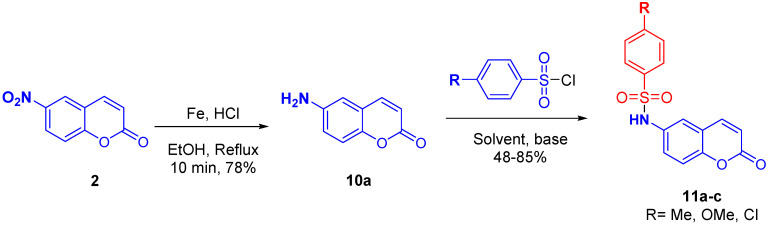
Synthesis of **11a**–**c** in two steps.

With the aim of improving the modest one-pot reaction yield, we went over the same conditions to obtain the desired N-(2-oxo-2*H*-chromen-6-yl)benzenesulfonamides **11a–c** in two steps (Scheme 5).

To begin with, we reduced 6-nitrocoumarin **2** with Fe powder at ethanol reflux, catalyzed with a few drops of HCl. The corresponding 6-amino-2*H*-chromen-2-one **10a**, obtained in good yield of 78%, was treated with arylsulfonyl chlorides under different synthetic conditions (Table 3).

The corresponding 4-methyl-N-(2-oxo-2*H*-chromen-6-yl)benzenesulfonamide **11a** was obtained at a 51% yield using potassium carbonate in water at room temperature (Entry 1). To optimize the reaction conditions, we substituted water with ethanol. After 6 h, 73% of the desired product **11a** was obtained at room temperature. We noticed that under ethanol reflux (Entry 3), the yield of compound **11a** was decreased. However, the use of pyridine as a solvent and base led to obtaining **11a** with an excellent yield of 85% (Entry 4). We applied those conditions to obtain **11b** and **11c** in good yields—71% and 82%, respectively.

The structures of the new compounds **10a** and **11a**–**c** were confirmed using NMR and mass spectrometry analysis (see experimental part and ESI, Figures S5–S20). The primary amine signal for 6-amino-2*H*-chromen-2-one **10a** appeared at 5.33 ppm. For the N-(2-oxo-2*H*-chromen-6-yl)benzenesulfonamides **11a**–**c**, the most relevant signal approving the coupling process was the signal at around 10–10.55 ppm corresponding to hydrogen of the secondary amine group, as well as the signals of the methyl group of **11a** and the methoxy group of **11b** at 2.27 and 3.79 ppm, respectively.

### 2.4. CuAAC Reaction of N-(2-oxo-2H-Chromen-6-yl)-N-(prop-2-ynyl)benzenesulfonamides

**Scheme 6:**
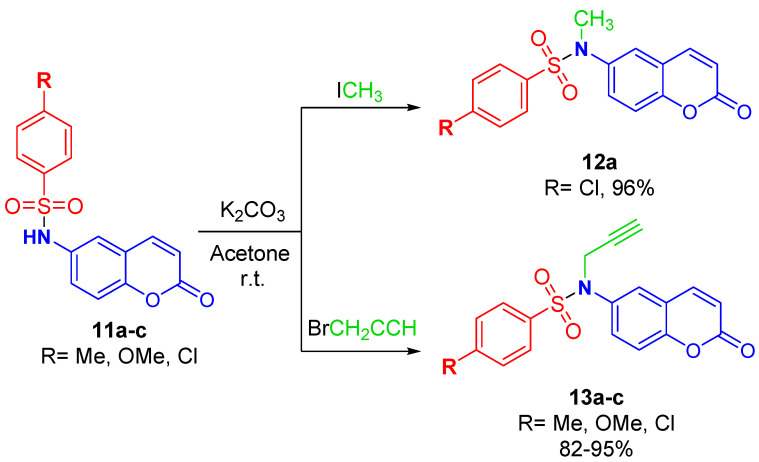
Synthesis of **12a** and **13a**–**c**.

The synthesis of coumarin–sulfonamide–nitroindazolyl–triazole hybrids was performed in two stages, first by alkylating the *N*-(2-oxo-2*H*-chromen-6-yl)benzenesulfonamides **11a**–**c** with a propargyl group and afterwards, linking them with nitroindazolyl-azide through a copper alkyne azide cycloaddition condition (CuAAC). Initially, we tried to alkylate **11a** with methyl iodide and propargyl bromide with the intention of obtaining the best experimental conditions for *N*-alkylation. With methyl iodide, 4-chloro-*N*-methyl-*N*-(2-oxo-2*H*-chromen-6-yl)benzenesulfonamide **12a** was obtained in a 96% yield, after 4 h at room temperature. The *N*-alkylation reaction with propargyl bromide was performed in acetone at room temperature in the presence of potassium carbonate, leading, after 12 h, to new *N*-(2-oxo-2*H*-chromen-6-yl)-*N*-(prop-2-ynyl)benzenesulfonamides **13a**–**c** in excellent yields (Scheme 6). The new compounds’ structures were fully confirmed using NMR spectroscopy and mass spectrometry analysis (Figures S21–S36).

**Scheme 7:**
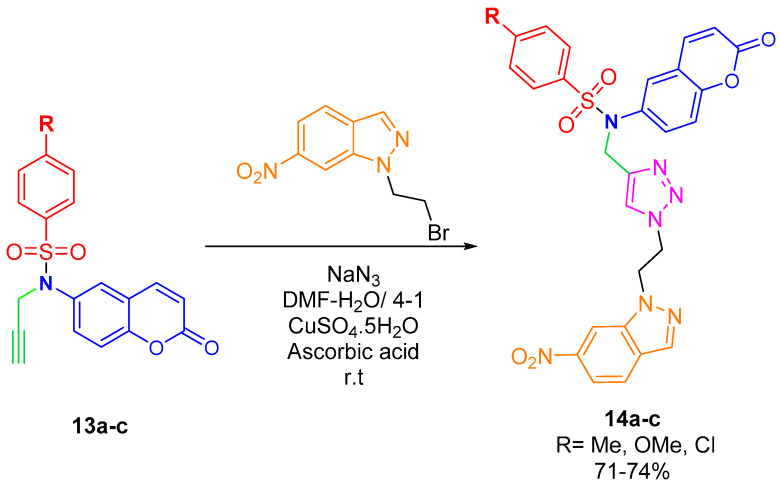
One-pot CuAAC reaction of **14a**–**c**.

The one-pot copper azide alkyne cycloaddition reaction between *N*-(2-oxo-2*H*-chromen-6-yl)-*N*-(prop-2-ynyl)benzenesulfonamides **13a**–**c** bearing *N*-terminal alkynes and 6-nitroindazole azide formed in situ using 1-(2-bromoethyl)-6-nitro-1*H*-indazole [62] was performed in a mixture of DMF and water (4:1) at room temperature in the presence of sodium azide, CuSO_4_.5H_2_O, and ascorbic acid. It led, after 16–24 h, to coumarin–sulfonamide–nitroindazolyl–triazole hybrids **14a**–**c** in good yields of 71–74% (Scheme 7).

The hybrid structures were characterized using NMR spectroscopy and high-resolution mass spectrometry (Figures S37–S48). The ^1^H and ^13^C NMR spectra of hybrids present some signals that are common to all prepared compounds. The ^1^H NMR spectra for **14a**–**c** showed the CH_2_-proton peaks linked to the triazole moiety at δ 4–5 ppm. The triazole proton signal appears as a singlet at around 7.7 ppm. The ^13^C spectra display all the signals of carbon and mainly the signals related to CH_2_ at δ 46–50 ppm, and carbonyl carbons appear at δ 160 ppm.

### 2.5. Biological Studies

Coumarin sulfonamides **11a**–**c**, their *N*-propargyl derivatives **13a**–**c**, and final compounds **14a**–**c** were tested as inhibitors of human isoforms of acetyl- and butyrylcholinesterase (AChE and BChE) and monoamine oxidases A and B (MAO-A, MAO-B). These biological targets were considered due to their structural similarity with previously investigated coumarin [63] and indazole-based [64,65] libraries acting as single- and dual-target inhibitors of AChE and MAO-B. The exploitation of a multitarget activity toward these key enzymes involved in neurodegenerative processes has been recognized as a promising new approach for the treatment of neurodegenerative diseases, particularly Alzheimer’s disease [66].

Results of inhibition assays reported in Table 4 witnessed a higher selectivity for AChE over BChE, and for MAO-A over its isoform B. The IC_50_ was calculated only for compounds having >60% inhibition at 10 µM. As a general remark, the lack of a basic moiety hampered a stable interaction with the catalytic site of cholinesterases, although a fair activity in AChE inhibition can be evidenced (42–60% inhibition). Tolylsulfonamide **11a** emerged as a good inhibitor of human AChE with an IC_50_ in the low micromolar range, comparable with that of reference drug galantamine.

Regarding MAO inhibition, data confirmed a known feature of coumarin sulfonamides as selective MAO-A inhibitors [67]. We retrieved comparable potencies with those previously reported [67], with tolylsulfonamide **11a** being the most effective MAO-A inhibitor with an IC_50_ in the same range as the reference drug pargyline, though with inverse selectivity (inhibition of MAO-A > MAO-B). Compounds **14a**, **14b**, and **14c** were less active than **11a**, but they maintain a significant efficacy despite the large size of these molecules. Apparently, the incorporation of the nitroindazolyltriazole unit is not a major obstacle to the building of new inhibitors of MAO-A.

### 2.6. Docking Studies

A molecular docking study was conducted to gain insights into the binding mode for the best compounds in the series, using the crystallographic structures of MAO-A and MAO-B available from the protein data bank (PDB: 2Z5X and 2V5Z, respectively). Docking models were built for each compound (**11a**–**c**/**13a**–**c**/**14a**–**c**) bound to the protein active site in order to calculate the empirical energy of interaction (ΔE) and the free energy of hydration (ΔG) (Table 5).

**Figure 5:**
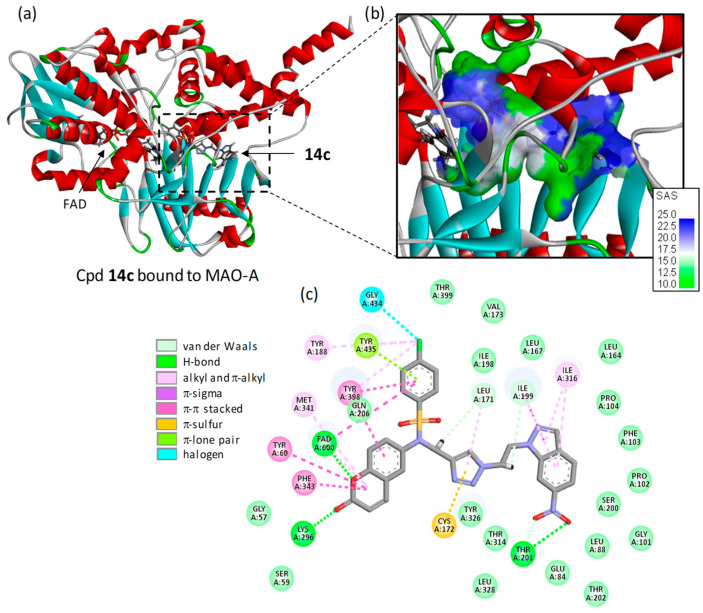
Molecular model of compound **14c** bound to MAO-A (PDB: 2Z5X). (**a**) A ribbon model of the entire protein with the FAD cofactor and **14c**. (**b**) Detailed view of the central ligand binding site with the solvent accessible surface (SAS color code indicated). (**c**) Binding map contacts (color code indicated).

**Figure 6:**
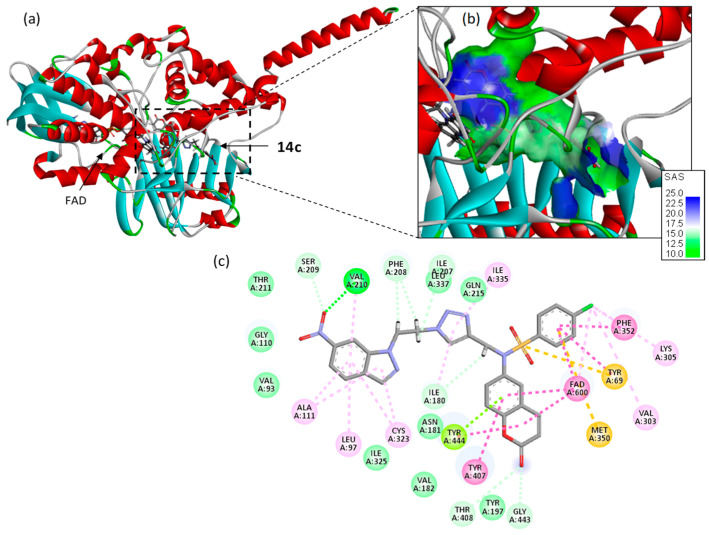
Molecular model of compound **14c** bound to MAO B (PDB: 2V5Z). (**a**) A ribbon model of the entire protein with the FAD cofactor and **14c**. (**b**) Detailed view of the central ligand binding site with the solvent accessible surface (SAS color code indicated). (**c**) Binding map contacts (color code indicated).

The disubstituted triazole derivatives **14a**–**c** provided significantly better binders than the benzenesulfonamides **13a**–**c** and the smaller coumarins **11a**–**c**. The incorporation of the nitroindazolyl–triazole unit significantly reinforces the enzyme interaction. The best compound in terms of docking was compound **14c**, which gave ΔE values a little more negative with MAO-A and MAO-B than with **14a**–**b**. The energy of hydration (ΔG) is also more favorable for **14c** compared to **14a**–**b** with MAO-B. With both enzymes, the indazolyl unit brought additional protein contacts and the appended nitro group served as an *H*-bond donor to further stabilize the ligand–protein complex. The nitro group of **14c** allows an *H*-bond interaction with residues Thr-201 and Val-210 for MAO-A and B, respectively (Figure 5 and Figure 6). Both the energy of the protein–ligand complexes (ΔE, the predominant parameter) and aqueous solvation of the molecules (ΔG, secondary parameter) were considered to select **14c** as the best ligand in the series.

The same configurations were observed with **14a** and **14b**, although the contact amino acids can be different (Figures S49 and S50). In each case, the three parts of the molecules participate in the protein interaction. The bulky size of compounds **14a**–**c** is not an obstacle to protein binding, but it does not translate into a potent enzyme inhibitory action.

## 3. Materials and Methods

### 3.1. General Remarks

Melting points were measured using Büchi-Tottoli capillary apparatus (Büchi, Flawil, Switzerland) and are uncorrected. Commercially available reagents were used without further purification. Reactions were monitored using thin-layer chromatography (TLC) using aluminum silica gel plates (silica gel 60, F 254 Merck (Merck KGaA, Darmstadt, Germany) 0.063–0.200 mm), and the spots were located with UV light (254 nm, 365 nm). Column chromatography was carried out on SiO_2_ (silica gel 60 Merck 0.063–0.200 mm). Thin-layer chromatography (TLC) was carried out on SiO_2_. ^1^H NMR and ^13^C NMR spectra were recorded in CDCl_3_ or DMSO-*d*_6_ and solution (unless otherwise specified), with TMS as an internal reference using a Bruker AC 300 (300 MHz) (Bruker, Billerica, MA, USA) (^1^H) or 75MHz (^13^C) instruments. Chemical shifts are given in δ parts per million (ppm). Multiplicities of ^13^C NMR resources were assigned by distortionless enhancement via polarization transfer (DEPT) experiments. High-resolution mass spectra (HRMS) were obtained with a Q-TOF Premier MALDI/ESI Tandem Mass Spectrometer (Waters Corporation, Milford, MA, USA).

### 3.2. Synthesis of 6-Nitro-2H-chromen-2-one *(**2**)*

In a 100 mL round-bottomed flask equipped with a magnetic stirrer, 2*H*-chromen-2-one 1 (6.84 mmol) and KNO_3_ (6.84 mol) were added to a concentrated H_2_SO_4_ solution (60 mL). The reaction was stirred for 24 h at room temperature and was then slowly poured over ice water (1 L), while maintaining stirring. The white precipitate obtained was filtered, washed with water, dried and purified using column chromatography on silica gel (eluent: AcOEt/Hexane; 20/80). White solid, yield: 92% (920 mg); mp: 199–201 °C; ^1^H NMR (DMSO-*d*_6_, 300 MHz): *δ* (ppm) 8.73 (H-5, d, *J* = 2.8 Hz, 1H), 8.42 (H-7, dd, *J* = 9.1, 2.8 Hz, 1H), 8.23 (H-8, d, *J* = 9.1 Hz, 1H), 7.62 (H-4, d, *J* = 9.7 Hz, 1H), 6.70 (H-3, d, *J* = 9.7 Hz, 1H); ^13^C NMR (DMSO-*d*_6_, 75 MHz) δ (ppm) 158.9 (CO), 157.2 (C8a), 143.5 (C6), 143.3 (C4), 126.5 (C8), 124.3 (C4a), 119.1 (C7), 118.1 (C3), 117.8 (C5); MS-ESI: m/z calculated for C_9_H_6_NO_4_ [M+H]^+^ 192,0297; found, 192,0302.

### 3.3. Synthesis of 6-Amino-2H-chromen-2-one *(**10a**)*

A 100 mL round-bottom flask was charged with 6-nitro-2*H*-chromen-2-one **2** (2.61 mmol) and ethanol (10 mL). Under vigorous agitation, iron powder (26.1 mmol) and a concentrated HCl solution (31.4 mmol) were added. The reaction mixture was heated over a period of 5 min. Once the starting material was consumed completely, the hot reaction mixture was cooled, filtered, and washed with ethanol. The filtrate was neutralized with a 10% aqueous NaOH solution and extracted with ethyl acetate. The crude mixture was then dried over Na_2_SO_4_ and the solvent was removed under reduced pressure. The crude product was purified using column chromatography on silica gel (eluent: AcOEt/Hexane; 40/60). Yellow solid; yield: 78% (390 mg); mp: 171–173 °C; ^1^H NMR (300 MHz, DMSO-*d*_6_) δ (ppm): 7.90 (H-4, d, *J* = 9.5 Hz, 1H), 7.12 (H-8, d, *J* = 8.8 Hz, 1H), 6.87 (H-7, dd, *J* = 8.8, 2.7 Hz, 1H), 6.77 (H-5, d, *J* = 2.7 Hz, 1H), 6.37 (H-3, d, *J* = 9.5 Hz, 1H), 5.33 (NH_2_, s, 2H); ^13^C NMR (75 MHz, DMSO-*d*_6_): *δ* (ppm) 160.5 (CO), 145.4 (C8a), 145.2(C6), 144.4 (C4), 119.1 (C8), 118.78 (C3), 116.6 (C7), 115.9 (C4a), 110.3 (C5); MS-ESI: m/z calculated for C_9_H_8_NO_2_ [M+H]^+^162,0555; found, 162,0555.

### 3.4. Procedure for the Preparation of N-(2-oxo-2H-Chromen-6-yl)benzenesulfonamides *(**11a**–**c**)*

Method A: To an equimolar amount of 6-nitro-2*H*-chromen-2-one **2** and the appropriate arylsulfonyl chloride in ethanol (5 mL), iron powder (10 equiv.) was added. The mixture was heated at reflux for 2 h. The reaction mixture was filtered and washed with ethanol. The solvent was evaporated and the crude product was purified using column chromatography on silica gel (eluent: AcOEt/Hexane; 20/80).

Method B: An equimolar amount of 7-amino-2*H*-chromen-2-one **10a** and the appropriate arylsulfonyl chloride in pyridine (5 mL) were stirred at room temperature for 1 h. A 5% aqueous HCl solution was added and a white precipitate was formed. The resulting solid was collected, washed with water several times, dried, and purified using column chromatography on silica gel (eluent: AcOEt/Hexane; 20/80).

Method C: Potassium carbonate (3 equiv.) was added to an equimolar amount of 6-amino-2*H*-chromen-2-one **10a** and the appropriate arylsulfonyl chloride in EtOH (5 mL). The reaction mixture was stirred at room temperature for 24 h. A total of 20 mL of water was added and the mixture was extracted with CH_2_Cl_2_. After evaporation of solvents, the crude product was purified using column chromatography on silica gel (eluent: AcOEt/Hexane; 20/80).

*4-Methyl-N-(2-oxo-2H-chromen-6-yl)benzenesulfonamide* (**11a**). Brown solid; yield: 85% (85 mg); mp: 250–252 °C; ^1^H NMR (300 MHz, DMSO-*d*_6_): *δ* (ppm) 10.35 (*NH*, s, 1H), 7.99 (H-4, d, *J* = 9.6 Hz, 1H), 7.60 (H_Ar_, d, *J* = 8.0 Hz, 2H), 7.39 (H-5, d, *J* = 2.5 Hz, 1H), 7.29 (H_Ar_, d, *J* = 8.0 Hz, 2H), 7.25 (H-8, d, *J* = 8.9 Hz, 1H), 7.20 (H-7, dd, *J* = 8.9, 2.5 Hz, 1H), 6.41 (H-3, d, *J* = 9.6 Hz, 1H), 2.28 (-CH_3_, s, 3H).^13^C NMR (126 MHz, DMSO-*d*_6_): *δ* (ppm) 160.3 (CO), 150.8 (C8a), 144.5(C4), 144.0(C6), 136.8 (C-SO_2_), 134.6 (C-Me), 130.3 (C_Ar_), 127.3 (C_Ar_), 125.1(C8), 119.7(C3), 119.6 (C4a), 117.8 (C7), 117.4 (C5), 21.48 (-CH_3_); MS-ESI: *m*/*z* calculated for C_16_H_14_NO_4_S [M+H]^+^ 316,0644; found, 316,0642.

*4-Methoxy-N-(2-oxo-2H-chromen-6-yl)benzenesulfonamide* (**11b**). White solid; yield: 71% (71 mg); mp: 202–204 °C; ^1^H NMR (300 MHz, DMSO-*d*_6_): *δ* (ppm) 10.35 (*NH*, s, 1H), 8.05 (H-4, d, *J* = 9.6 Hz, 1H), 7.71 (H_Ar_, d, *J* = 9.0 Hz, 2H), 7.45 (H-5, d, *J* = 2.2 Hz, 1H), 7.34–7.22 (H-8, H-7,m, 2H), 7.06 (H_Ar_, d, *J* = 9.0 Hz, 1H), 6.47 (H-3, d, *J* = 9.6 Hz, 1H), 3.79 (-OCH_3_, s, 3H).^13^C NMR (126 MHz, DMSO-*d*_6_): *δ* (ppm) 162.5 (CO), 159.7 (C-OMe), 150.2 (C8a), 143.9(C4), 134.2 (C6), 130.7 (C-SO_2_), 128.9 (C_Ar_), 124.5 (C8), 119.1(C3), 119.0(C4a), 117.2 (C7), 116.8 (C5), 114.4 (C_Ar_), 55.6 (-OCH_3_); MS-ESI: *m*/*z* calculated for C_16_H_14_NO_5_S [M+H]^+^ 332,0593; found, 332,0593.

*4-Chloro-N-(2-oxo-2H-chromen-6-yl)benzenesulfonamide* (**11c**). Yellow solid; yield: 82% (85 mg); mp: 234–236 °C; ^1^H NMR (300 MHz, DMSO-*d*_6_): *δ* (ppm) 10.35 (*NH*, s, 1H, D_2_O and HDO exchangeable), 7.93 (H-4, d, *J* = 9.6 Hz, 1H), 7.69 (H_Ar_, d, *J* = 8.7 Hz, 2H), 7.55 (H_Ar_, d, *J* = 8.7 Hz, 1H), 7.35 (H-5, d, *J* = 2.5 Hz, 1H), 7.24 (H-8,d, *J* = 8.9 Hz, 1H), 7.19 (H-7,d, *J* = 8.9, 2.5 Hz, 1H), 6.40 (H-3, d, *J* = 9.6 Hz, 1H);^13^C NMR (126 MHz, DMSO-*d*_6_): *δ* (ppm) 160.5 (CO), 151.0 (C8a), 144.4 (C4), 138.6 (C-Cl), 138.3 (C6), 134.1 (C-SO_2_), 130.0 (C_Ar_),129.2 (C_Ar_), 125.6 (C8), 120.4 (C3), 119.6 (C4a), 117.9 (C7), 117.4 (C5); MS-ESI: *m*/*z* calculated for C_15_H_11_NO_4_SCl [M+H]^+^ 336,0097; found, 336,0096.

### 3.5. N-Alkylation of *(**11a**–**c**)*

#### 3.5.1. General Procedure for Synthesis of 4-Chloro-N-methyl-N-(2-oxo-2H-chromen-6-yl)benzenesulfonamide (**12a**)

Potassium carbonate (0.87 mmol) and methyl iodide (0.32 mmol) were added to a stirring solution of 4-chloro-N-(2-oxo-2H-chromen-7-yl)benzenesulfonamide **11c** (0.29 mmol) in acetone (5 mL) at room temperature. The reaction was monitored using TLC until the starting material was consumed completely. The solvent was evaporated and the crude product was purified using column chromatography on silica gel (eluent: AcOEt/Hexane; 20/80). White solid, yield: 96% (96 mg); mp: 244–246 °C; ^1^H NMR (300 MHz, DMSO-*d*_6_): *δ* (ppm) 8.04 (H-4, d, *J* = 9.6 Hz, 1H), 7.69 (H_Ar_, d, *J* = 8.7 Hz, 2H), 7.63–7.49 (H_Ar_, H-5, m, 3H), 7.46–7.25 (H-8, H-7,m, 2H), 6.55 (H-3, d, *J* = 9.6 Hz, 1H), 3.18 (-NCH_3_, s, 3H); ^13^C NMR (126 MHz, DMSO-*d*_6_): *δ* (ppm) 159.7 (CO), 152.2 (C8a), 143.7 (C4), 138.5 (C-Cl), 136.9 (C6), 134.3 (C-SO_2_), 130.0 (C8),129.5 (C_Ar_), 129.4 (C_Ar_),126.1 (C3), 119.0 (C4a), 117.0 (C7), 116.9 (C5),37.9 (-*N*CH_3_); MS-ESI: *m*/*z* calculated for C_16_H_13_NO_4_SCl [M+H]^+^ 350,0254; found, 350,0266.

#### 3.5.2. General Procedure for Synthesis of N-(2-oxo-2*H*-Chromen-6-yl)-N-(prop-2-ynyl)benzenesulfonamides (**13a**–**c**)

Potassium carbonate (3 equiv.) and propargyl bromide (1.2 equiv.) were added to a solution of appropriate *N*-(2-oxo-2H-chromen-7-yl)benzenesulfonamides **11a**–**c** (1 equiv.) in acetone (5 mL). The reaction mixture was stirred overnight at room temperature. The solvent was removed under reduced pressure and the crude residue was purified using chromatography on silica gel (eluent: AcOEt/Hexane; 20/80).

*4-Methyl-N-(2-oxo-2H-chromen-6-yl)-N-(prop-2-yn-1-yl)benzenesulfonamide* (**13a**). White solid; yield: 95% (95 mg); mp:128–130 °C; ^1^H NMR (300 MHz, DMSO-*d*_6_): *δ* (ppm) 8.01 (H-4, d, *J* = 9.6 Hz, 1H), 7.58 (H-5, d, *J* = 2.5 Hz, 1H), 7.47 (HAr, d, *J* = 8.7 Hz, 2H), 7.38–7.32 (H_Ar_, H-8, m, 3H), 7.28 (H-7, dd, *J* = 8.8, 2.5 Hz, 1H), 6.48 (H-3, d, *J* = 9.6 Hz, 1H), 4.46 (-CH_2_, d, *J* = 2.4 Hz, 2H), 3.20 (≡CH, t, *J* = 2.4 Hz, 1H), 3.18 (Ar-CH_3_, s, 3H); ^13^C NMR (126 MHz, DMSO-*d*_6_): *δ* (ppm) 160.2 (CO), 153.2 (C8a), 144.6 (C6), 144.2 (C4), 135.3 (C-SO_2_), 135.0 (C-Me), 131.8 (C8),130.4 (CAr),128.8 (C3), 128.1 (C_Ar_),119.6 (C4a), 117.6 (C7), 117.5 (C5), 78.71(≡CH), 77.17(-C≡), 41.0 (-CH_2_-), 21.6(Ar-CH_3_); MS-ESI: *m*/*z* calculated for C_19_H_16_NO_4_S [M+H]^+^ 354,0800; found, 354,0809.

*4-Methoxy-N-(2-oxo-2H-chromen-6-yl)-N-(prop-2-yn-1-yl)benzenesulfonamide* (**13b**). Yellow solid; yield: 82% (82 mg); mp:135–137 °C; ^1^H NMR (300 MHz, DMSO-*d*_6_): *δ* (ppm): 8.00 (H-4, d, *J* = 9.5 Hz, 1H), 7.57 (H-5, d, *J* = 2.6 Hz, 1H), 7.51 (H_Ar_, d, *J* = 9.0 Hz, 2H), 7.36 (H-8,d, *J* = 8.9 Hz, 1H), 7.29 (H-7, dd, *J* = 8.9, 2.6 Hz, 1H), 7.05 (H_Ar_, d, *J* = 9.0 Hz, 2H), 6.49 (H-3, d, *J* = 9.6 Hz, 1H), 4.44 (-CH_2_, d, *J* = 2.5 Hz, 2H), 3.80 (O-CH_3_, s, 3H).3.19 (≡CH, t, *J* = 2.5 Hz, 1H). ^13^C NMR (126 MHz, DMSO-*d*_6_): *δ* (ppm) 163.5 (CO), 160.2 (C-OMe), 153.1 (C8a), 144.2 (C4), 135.4 (C6), 131.8(C8), 130.3(C_Ar_),129.3 (C-SO_2_), 128.7 (C3), 119.5 (C4a), 117.6 (C7), 117.5 (C5), 115.1 (C_Ar_),78.8(≡CH)., 77.1(-C≡), 56.3 (O-CH_3_), 41.0 (-CH_2_-); MS-ESI: *m*/*z* calculated for C_19_H_16_NO_5_S [M+H]^+^ 370,0749; found, 370,0763.

*4-Chloro-N-(2-oxo-2H-chromen-6-yl)-N-(prop-2-yn-1-yl)benzenesulfonamide* (**13c**). White solid; yield: 83% (83 mg); mp: 165–167 °C; ^1^H NMR (300 MHz, DMSO-*d*_6_): *δ* (ppm) 8.07 (H-4, d, *J* = 9.6 Hz, 1H), 7.74–7.57 ((H_Ar_, H-5, m, 5H), 7.45 (H-8, d, *J* = 8.8 Hz, 1H), 7.38 (H-7, dd, *J* = 8.9, 2.5 Hz, 1H), 6.56 (H-3, d, *J* = 9.6 Hz, 1H), 4.55 (-CH_2_, d, *J* = 2.5 Hz, 2H), 3.29 (≡CH, t, *J* = 2.5 Hz, 1H); ^13^C NMR (126 MHz, DMSO-*d*_6_): *δ* (ppm) 159.6 (CO), 152.8 (C8a), 143.6 (C4), 138.5 (C-Cl), 136.1 (C6), 134.4 (C-SO_2_), 131.4(C8), 129.6 (C_Ar_), 129.4 (C_Ar_), 128.3 (C3), 119.1 (C4a), 117.2 (C7), 117.0 (C5), 77.9(≡CH), 76.9(-C≡), 40.7(-CH_2_-); MS-ESI: *m*/*z* calculated for C_18_H_13_NO_4_SCl [M+H]^+^ 374,0254; found, 374,0258.

### 3.6. General Procedure for 1,3-Dipolar Cycloaddition of Azides with Terminal Alkynes

Copper sulfate (0.8 mmol) and ascorbic acid (0.8 mmol) were added to a mixture of 1-(2-azidoethyl)-6-nitro-1H-indazole (0.8 mmol), sodium azide NaN_3_ (5 equiv., 4 mmol), and *N*-(2-oxo-2H-chromen-7-yl)-*N*-(prop-2-ynyl)benzenesulfonamides **13a**–**c** (0.8 mmol) in 4 mL of DMF/H_2_O (4:1). After 16 h–24 h, the starting material was consumed completely, as monitored using TLC. The reaction mixture was filtered, washed with water, and extracted with ethyl acetate. The solvent was removed under reduced pressure and the crude residues were purified using chromatography on silica gel (eluent: AcOEt/Hexane; 50/50).

*4-Methyl-N-((1-(2-(6-nitro-1H-indazol-1-yl)ethyl)-1H-1,2,3-triazol-4-yl)methyl)-N-(2-oxo-2H-chromen-6-yl)benzenesulfonamide* (**14a**). White solid; yield: 72% (203 mg); mp: 146–148 °C; ^1^H NMR (300 MHz, DMSO-*d*_6_): *δ* (ppm) 8.46 (H-7’, t, *J* = 1.8 Hz, 1H), 8.15 (H-3’, d, *J* = 0.9 Hz, 1H), 7.93–7.82 (H-4, H-5, H-5’, m, 3H), 7.74 (H-triazole, s, 1H), 7.43 (H_Ar_, d, *J* = 8.3 Hz, 2H), 7.37–7.31 (H_Ar_, H-8,m, 3H), 7.20 (H-4’, d, *J* = 8.9 Hz, 1H), 7.07 (H-7, dd, *J* = 8.9, 2.6 Hz, 1H), 6.45 (H-3, d, *J* = 9.6 Hz, 1H), 4.95 (Triazole-CH_2_, dd, *J* = 6.6, 4.8 Hz, 2H), 4.75 (Indazole-CH_2_, dd, *J* = 6.6, 4.8 Hz, 2H), 4.69 (CH_2_-*N*-Coumarin, s, 2H), 2.35 (Ar-CH_3_, s, 3H); ^13^C NMR (126 MHz, DMSO-*d*_6_): *δ* (ppm) 160.2 (CO), 152.9 (C8a), 144.2 (C7a’), 142.6 (C7), 141.4 (C6’), 138.7, 135.0, 134.7 (C3’), 133.2, 132.3, 130.4 (C_Ar_), 128.8 (C3), 127.9 (C_Ar_), 124.9, 122.7, 120.6, 119.3 (C4a), 117.3 (C7), 115.6 (C5’), 107.1 (C7’), 106.1, 49.6 (-CH_2_-), 49.0 (-CH_2_-), 46.0 (-CH_2_-), 21.6 (Ar-CH_3_); MS-ESI: *m*/*z* calculated for C_28_H_24_N_7_O_6_S [M+H]^+^ 586,1509; found, 586,1505.

*4-Methoxy-N-((1-(2-(6-nitro-1H-indazol-1-yl)ethyl)-1H-1,2,3-triazol-4-yl)methyl)-N-(2-oxo-2H-chromen-6-yl)benzenesulfonamide* (**14b**). White solid; yield: 71% (209 mg); mp: 252–254 °C; ^1^H NMR (300 MHz, DMSO-*d*_6_): *δ* (ppm) 8.47 (H-7’, t, *J* = 1.8 Hz, 1H), 8.15 (H-3’, d, *J* = 1.0 Hz, 1H), 7.94–7.82 (H-4, H-5, H-5’, m, 3H), 7.73 (H-triazole, s, 1H), 7.46 (H_Ar_, d, *J* = 9.0 Hz, 2H), 7.35 (H-8, d, *J* = 2.6 Hz, 1H), 7.20 (H-4’, d, *J* = 8.9 Hz, 1H), 7.07 (H-7, dd, *J* = 8.9, 2.6 Hz, 1H), 7.04 (H_Ar_, d, *J* = 9.0 Hz, 2H), 6.45 (H-3, d, *J* = 9.6 Hz, 1H), 4.95 (triazole-CH_2_, t, *J* = 5.7 Hz, 2H), 4.75 (indazole-CH_2_, t, *J* = 5.7 Hz, 2H), 4.67 (CH_2_-*N*-coumarin, s, 2H), 3.80 (OCH_3_, s, 3H); ^13^C NMR (126 MHz, DMSO-*d*_6_): *δ* (ppm) 163.4 (CO), 160.2 (C-OMe), 152.9 (C8a), 146.4 (C4), 144.2 (C7), 142.6 (C6’), 138.7, 135.2, 134.7 (C3’), 132.4 (C_Ar_), 130.2, 129.3, 128.8 (C3), 127.1, 124.9, 122.7,119.3 (C4a), 117.3 (C7), 115.6 (C5’), 115.1 (C_Ar_), 107.1 (C7’), 56.3 (O-CH_3_), 49.6 (-CH_2_-), 49.0 (-CH_2_-), 46.0 (-CH_2_-); MS-ESI: *m*/*z* calculated for C_28_H_24_N_7_O_7_S [M+H]^+^ 602,1458; found, 602,1464.

*4-Chloro-N-((1-(2-(6-nitro-1H-indazol-1-yl)ethyl)-1H-1,2,3-triazol-4-yl)methyl)-N-(2-oxo-2H-chromen-6-yl)benzenesulfonamide* (**14c**). White solid; yield: 74% (220 mg); mp: 200–202 °C; ^1^H NMR (300 MHz, DMSO-*d*_6_): *δ* (ppm) 8.47 (H-7’, t, *J* = 1.6 Hz, 1H), 8.15 (H-3’, d, *J* = 1.0 Hz, 1H), 7.94–7.82 (H-4, H-5, H-5’, m, 3H), 7.76 (H-triazole, s, 1H), 7.60 (H_Ar_, d, *J* = 8.8 Hz, 2H), 7.54 (H_Ar_, d, *J* = 8.8 Hz, 2H), 7.38 (H-8, d, *J* = 2.6 Hz, 1H), 7.22 (H-4’, d, *J* = 8.8 Hz, 1H), 7.11 (H-7, dd, *J* = 8.9, 2.6 Hz, 1H), 6.46 (H-3, d, *J* = 9.6 Hz, 1H), 4.96 (triazole-CH_2_, t, *J* = 5.6 Hz, 2H), 4.75 (indazole-CH_2_, t, *J* = 5.6 Hz, 2H), 4.72 (CH_2_-*N*-coumarin, s, 2H); ^13^C NMR (126 MHz, DMSO-*d*_6_): *δ* (ppm) 160.1 (CO), 153.1 (C8a), 146.4 (C7a’), 144.2 (C6), 142.4 (C6’), 138.9, 138.7, 136.7, 134.7, 134.6 (C3’), 132.5, 130.1 (C_Ar_), 129.8 (C_Ar_), 128.9, 127.1, 125.0, 122.7, 119.5 (C4a), 117.5, 117.4 (C7), 115.6 (C5’), 107.1 (C7’), 49.6 (-CH_2_-), 49.0 (-CH_2_-), 46.3 (-CH_2_-). MS-ESI: *m*/*z* calculated for C_27_H_21_N_7_O_6_SCl [M+H]^+^ 606,0963; found, 606,0958.

### 3.7. Computational Details

The *ω*B97X-D [68] functionals, together with the standard 6-311G(d,p) basis set [69] were used in this MEDT study. The TSs were characterized by the presence of only one imaginary frequency. The Berny method was used in optimizations [70,71]. Solvent effects were taken into account by full optimization of the gas phase structures at the same computational level using the polarizable continuum model [72,73] (PCM). Values of *ω*B97X-D/6-311G(d,p) enthalpies, entropies, and Gibbs free energies in methanol were calculated with standard statistical thermodynamics at 298.15 K and 1 atm [69], using PCM frequency calculations at the solvent optimized structures.

The GEDT [53] values were computed using the following equation: GEDT(f) = Σqf, where q is the natural charge [59,60] of the atoms belonging to one of the two frameworks (f) at the TS geometries. Global CDFT indices [48,49] were calculated using the equations given in reference [49].

The Gaussian suite of programs [74] was used to perform the calculations. ELF [56] analyses of the *ω*B97X-D/6-311G(d,p) monodeterminantal wave functions were performed using the TopMod [75] package with a cubical grid with a step size of 0.1 Bohr. Molecular geometries and ELF basin attractors were visualized using the GaussView program [76].

### 3.8. Biological Methods

Human recombinant AChE, BChE from human serum, and human MAOs from baculovirus-infected insect cells were purchased from Sigma Aldrich (Milan, Italy). Incubations were carried out in 96-well plates (Greiner Bio-One, Kremsmünster, Austria) and spectrometric measures were achieved with an Infinite M1000 Pro plate reader (Tecan, Cernusco s.N., Milan, Italy). Inhibition data and IC_50_s were calculated with Prism (version 5.01 for Windows; GraphPad Software, San Diego, CA, USA). All experiments were carried out in triplicate and results expressed as mean ± SEM.

AChE/BChE and MAO A/B inhibition were, respectively, determined with the spectrophotometric method of Ellman, and a fluorimetric method using kynuramine as MAO substrate, as previously described [77].

### 3.9. Molecular Docking Procedure

The tridimensional structures of monoamine oxidases A and B were retrieved from the Protein Data Bank (www.rcsb.org) under the PDB codes 2Z5X and 2V5Z, respectively [78,79]. The GOLD 5.3 software (Cambridge Crystallographic Data Centre, Cambridge, UK) was used to perform molecular docking analysis. Prior to the docking operations, the structure of each ligand was optimized using a classical Monte Carlo conformational searching procedure via the BOSS software, version 4.9 [80]. Molecular graphics and analysis were performed using Discovery Studio Visualizer, Biovia 2020 (Dassault Systèmes BIOVIA Discovery Studio Visualizer 2020, San Diego, CA, USA, Dassault Systèmes, 2020). With both MAO-A and -B, the active site was defined by the ligand included in the crystallographic structure. During the process, the side chains of the following amino acids within the binding site were rendered fully flexible: Tyr69, Leu97, Phe108, Tyr197, Ile207, Phe208, Phe352, Tyr407, Trp441, and Tyr444 (for MAO-A), and Phe99, Phe103, Phe118, Trp119, Phe168, Tyr188, Tyr326, Phe343, Tyr398, and Tyr435 (for MAO-B). A docking grid centered in the volume defined by the central amino acid has been defined based on shape complementarity and geometry considerations. In general, up to 100 poses considered to be energetically reasonable are selected during the search for the correct binding mode of the ligand. The decision to select a trial pose is based on ranked poses, using the fitness scoring function (PLP score). The same procedure was used to establish molecular models for all compounds. The procedure has been previously described with other protein–ligand complexes [81,82].

## 4. Conclusions and Perspectives

Multifunctional agents targeting monoamine oxidases and cholinesterases are actively searched to improve the treatment of diseases associated with a cognitive decline, notably Alzheimer’s and Parkinson’s diseases. In this context, we have designed a novel extended scaffold comprising a coumarin unit coupled to sulfonamide and nitroindazolyltriazole units.

The mechanism and regioselectivity in the initial nitration reaction of coumarin **1** have been studied within the MEDT. In sulfuric acid solution, coumarin: SO_4_H_2_ complex **6** is formed, which undergoes an SEA reaction with NO_2_^+^ ion **4**. The subsequent nitration reaction proceeds via a two-step mechanism with the formation of a tetrahedral cation intermediate, **IN-C6**. The preferential electrophilic attack on the C6 carbon of the aromatic coumarin ring determines the regioselectivity in this SEA reaction.

The branching and elongation of the scaffold led to hybrid compounds (**14a**–**c**), which maintain a significant activity against MAO-A. The synthesis of these compounds is well controlled, with a precise mechanistic analysis of the initial nitration step for the coumarin core. This knowledge provides the necessary background for the further design and synthesis of new molecules. The selectivity for MAO-A versus MAO-B is interesting and may be exploited for the design of compounds specifically targeting depression and anxiety. Antidepressant agents with selectivity for the MAO-A isoform are actively being researched [83,84,85,86].

Currently, we are engaged in the synthesis of additional compounds in the series, including slightly shorter molecules maintaining a marked preference for MAO-A vs. MAO-B.

## Figures and Tables

**Table 1:** B3LYP/6-311G(d,p) electronic chemical potential, µ; chemical hardness, η; electrophilicity, ω; and nucleophilicity, *N* in eV of coumarin **1**, coumarin: SO_4_H_2_ complex **6**, and nitronium NO_2_^+^ ion **4** and benzene **3**.

	µ	ɳ	ω	*N*
Nitronium NO_2_^+^ ion **4**	−16.18	9.35	14.01	−11.74
Coumarin: SO_4_H_2_ complex **6**	−5.09	4.62	2.81	1.72
Coumarin **1**	−4.19	4.62	1.90	2.62
Benzene **3**	−3.30	6.80	0.80	2.42

**Table 3:** Study of optimization conditions of the *N*-coupling reaction of 6-amino-2*H*-chromen-2-one **10a** with arylsulfonyl chlorides.

Entry	Product	Solvent	Base	T °C	Time (h)	Yield %
1	**11a**	H_2_O	K_2_CO_3_	r.t.	24	51
2	**11a**	EtOH	K_2_CO_3_	r.t.	6	73
3	**11a**	EtOH	K_2_CO_3_	80 °C	4	48
4	**11a**	-	Pyridine	r.t.	1	85
5	**11b**	-	Pyridine	r.t.	1	71
6	**11c**	-	Pyridine	r.t.	1	82

**Table 4:** Biological activities of compounds **11a**–**c**, **13a**–**c**, and **14a**–**c** ^(a)^.

	*h*AChE		*h*BChE	*h*MAO-A		*h*MAO-B
	% Inhibition	IC_50_ µM	% Inhibition	% Inhibition	IC_50_ µM	% Inhibition
**11a**	55 ± 3		ni	76 ± 3	**2.46 ± 0.67**	45 ± 2
**11b**	57 ± 4		ni	26 ± 3		34 ± 3
**11c**	55 ± 3		ni	9 ± 4		26 ± 4
**13a**	60 ± 3	**3.73 ± 0.37**	ni	13 ± 5		12 ± 1
**13b**	49 ± 3		ni	22 ± 7		28 ± 2
**13c**	48 ± 2		ni	37 ± 5		20 ± 2
**14a**	51 ± 2		48 ± 4	76 ± 5	**14.8 ± 1.6**	39 ± 3
**14b**	44 ± 3		31 ± 5	81 ± 5	**16.9 ± 1.9**	23 ± 4
**14c**	42 ± 1		ni	67 ± 5	**28.0 ± 2.2**	45 ± 1
**Galantamine**		**0.721 ± 0.152**	**8.78 ± 0.36 ^(b)^**			
**Pargyline**					**10.9 ± 0.6**	**2.69 ± 0.48 ^(b)^**

^(a)^ % Inhibition calculated at 10 µM concentration; ni: no inhibition. ^(b)^ IC_50_ µM. Values are mean ± SEM (*n* = 3).

**Table 5:** Calculated potential energy of interaction (ΔE) and free energy of hydration (ΔG) for the interaction of the compounds with MAO-A and B.

Compound	Monoamine Oxidase A (MAO-A)		Monoamine Oxidase B (MAO-B)	
	ΔE (kcal/mol)	ΔG (kcal/mol)	ΔE (kcal/mol)	ΔG (kcal/mol)
**11a**	−52.30	−22.20	−53.85	−15.00
**11b**	−56.65	−18.40	−59.70	−17.65
**11c**	−56.65	−24.30	−58.40	−18.50
**13a**	−56.80	−17.20	−58.70	−14.80
**13b**	−59.40	−14.50	−66.60	−15.50
**13c**	−56.10	−18.75	−60.55	−16.75
**14a**	−89.60	−24.70	−94.30	−26.60
**14b**	−89.65	−36.30	−94.40	−20.25
**14c**	−91.75	−21.90	−97.40	−30.00
**Harmine**	−32.35	−9.00	-	-
**Safinamide**	-	-	−53.80	−21.55

## Data Availability

Data is contained within the article or Supplementary Materials.

## Supplementary Materials

The following supporting information can be downloaded at: https://www.mdpi.com/article/10.3390/ijms25126803/s1.
