# Supplementary material for: Synthesis, Molecular Electron Density Theory Study, Molecular Docking, and Pharmacological Evaluation of New Coumarin–Sulfonamide–Nitroindazolyl–Triazole Hybrids as Monoamine Oxidase Inhibitors
Source: Int J Mol Sci. 2024 Jun 20;25(12):6803. doi: 10.3390/ijms25126803 (PMC11203676; doi:10.3390/ijms25126803)
Supplement: Supplementary file 1 [file ijms-25-06803-s001.zip › ijms-3041643-supplementary.pdf]

# Supporting Information

## Synthesis, Molecular Electron Density Theory Study, Molecular Docking, and Pharmacological Evaluation of New Coumarin–Sulfonamide–Nitroindazolyl–Triazole Hybrids as Monoamine Oxidase Inhibitors

Mohammed Eddahmi,[1] Gabriella La Spada,[2] Luis R. Domingo,[3] Gérard Vergoten,[4] Christian Bailly,[4,*] Marco Catto,[2] Latifa Bouissane [1,*]

[1] Molecular Chemistry, Materials and Catalysis Laboratory, Faculty of Sciences and Technologies, Sultan Moulay Slimane University, BP 523, 23000 Beni-Mellal, Morocco.

[2] Department of Pharmacy-Pharmaceutical Sciences, University of Bari Aldo Moro, via E. Orabona 4, 70125 Bari, Italy.

[3]Department of Organic Chemistry, University of Valencia, Dr. Moliner 50, 46100 Burjassot, Valencia, Spain.

[4] Institute of Pharmaceutical Chemistry Albert Lespagnol (ICPAL), Faculty of Pharmacy, University of Lille, rue du Professeur Laguesse, BP-83, F-59006 Lille, France.

* Correspondence: christian.bailly@univ-lille.fr (C.B.); l.bouissane@usms.ma (L.B.)

**Table of contents**


| | | |
|---|---|---|
| **A.** | Thermodynamic properties in the EAS nitration reaction nitration reaction of coumarin:$SO_4H_2$ complex **6** with nitronium $NO_2^+$ ion **4** | **2** |
| **B.** | NMR and mass spectrum of compounds **2** and **10a** | **3** |
| **C.** | NMR and mass spectrum of compounds **11a-c** | **7** |
| **D.** | NMR and mass spectrum of compounds **12a** and **13a-c** | **13** |
| **E.** | NMR and mass spectrum of compounds **14a-c** | **21** |
| **F.** | Binding map contacts for compound **14a** and **14b** bound to MAO-A | **27** |

## A. Thermodynamic properties in the EAS nitration reaction nitration reaction of coumarin:$SO_4H_2$ complex 6 with nitronium $NO_2^+$ ion 4

**Table S1.** SCRF wB97X-D/6-311G(d,p) total enthalpies, H in a.u., entropies, S in kcal·mol$^{-1}$K$^{-1}$, and Gibbs free energies, G in a.u., computed at 25 ºC and 1 atm, in water, for the stationary points involved in the EAS nitration reaction of coumarin:$SO_4H_2$ complex **6** with nitronium $NO_2^+$ ion **4.**

| | H | S | G |
|---|---|---|---|
| Coumarin:$SO_4H_2$ **6** | -1197.050072 | 122.444 | -1197.108249 |
| $NO_2^+$ **4** | -204.804604 | 50.988 | -204.828829 |
| **MC-6** | -1401.855317 | 151.129 | -1401.927124 |
| **TS-C5** | -1401.847722 | 139.19 | -1401.913856 |
| **TS-C6** | -1401.850198 | 139.35 | -1401.916407 |
| **TS-C7** | -1401.845899 | 138.331 | -1401.911624 |
| **TS-C8** | -1401.847829 | 133.497 | -1401.911258 |
| **IN-C5** | -1401.856789 | 136.858 | -1401.921815 |
| **IN-C6** | -1401.868227 | 139.289 | -1401.934408 |
| **IN-C7** | -1401.857670 | 140.085 | -1401.924229 |
| **IN-C8** | -1401.867728 | 134.883 | -1401.931815 |
| **7** | -1401.525723 | 136.687 | -1401.590667 |
| **2** | -1401.533492 | 136.848 | -1401.598513 |
| **8** | -1401.531906 | 138.514 | -1401.597718 |
| **9** | -1401.530036 | 132.687 | -1401.593080 |

## B. NMR and mass spectrum of compounds 2 and 10a

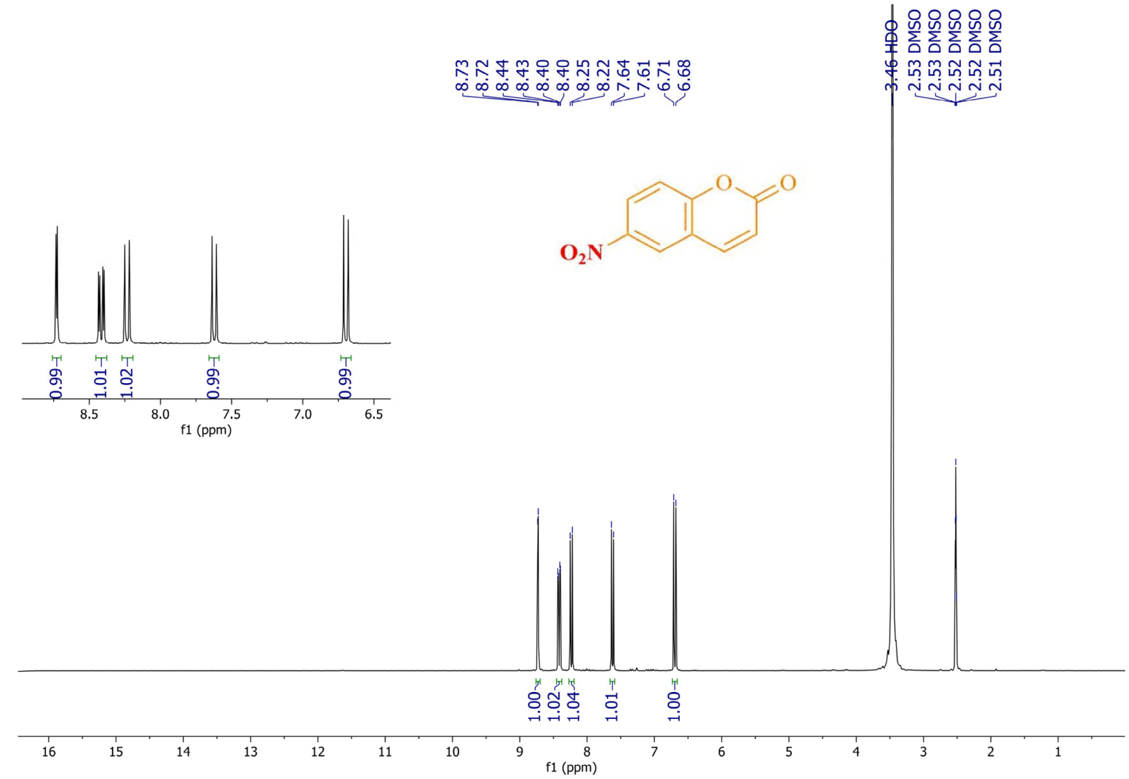


**Figure S1**. ¹H NMR spectrum of compound **2** in DMSO-$d_6$.

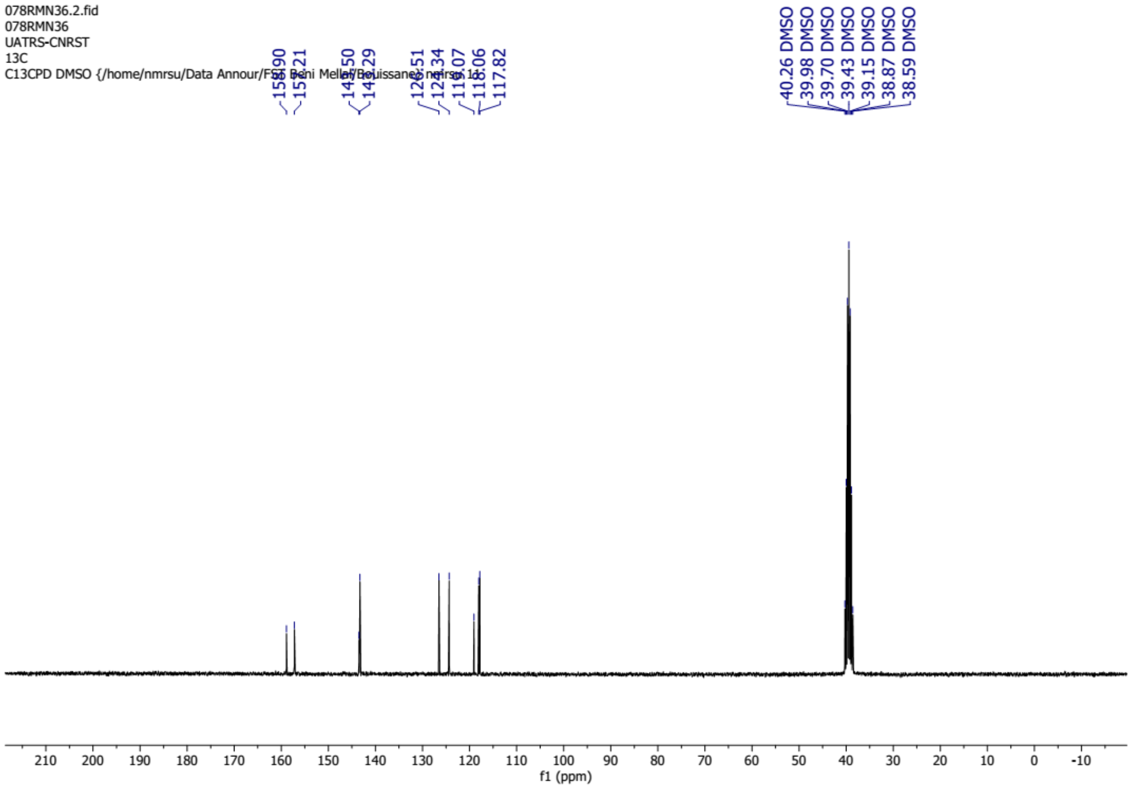


**Figure S2**. ¹³C NMR spectrum of compound **2** in DMSO-$d_6$.

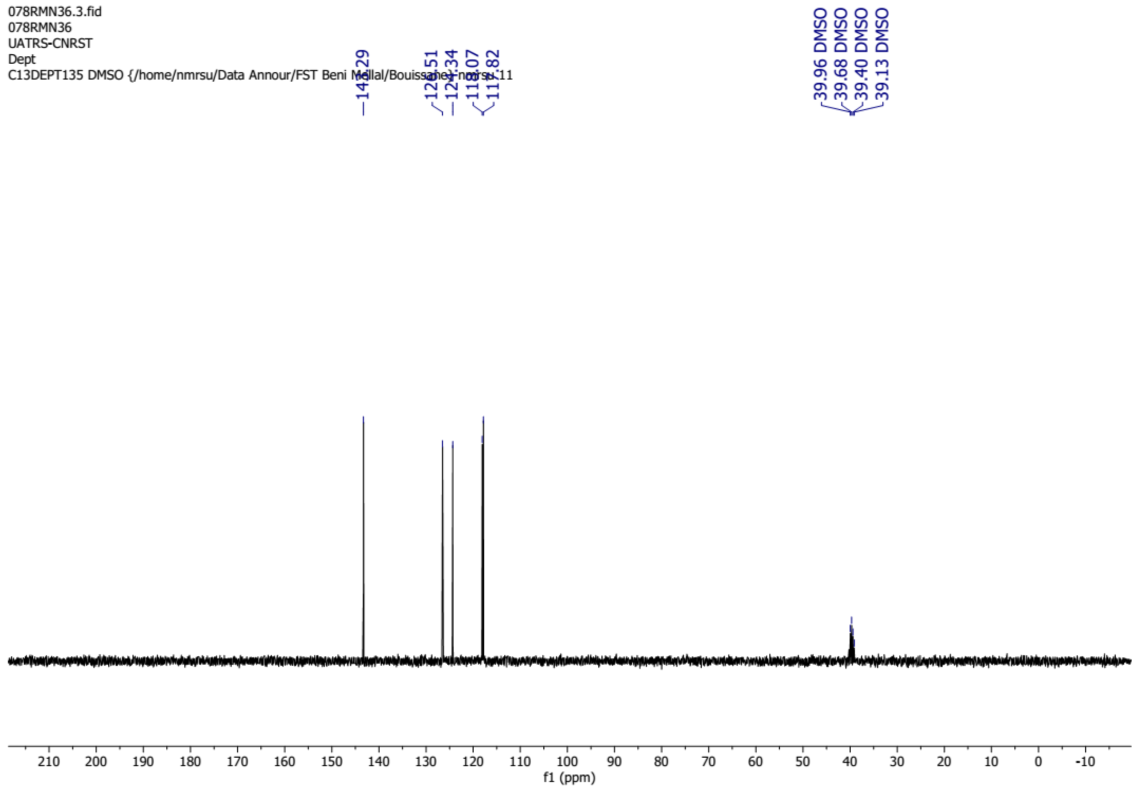


**Figure S3**. $^{13}C$ NMR, DEPT 135 spectrum of compound **2** in DMSO-$d_6$.

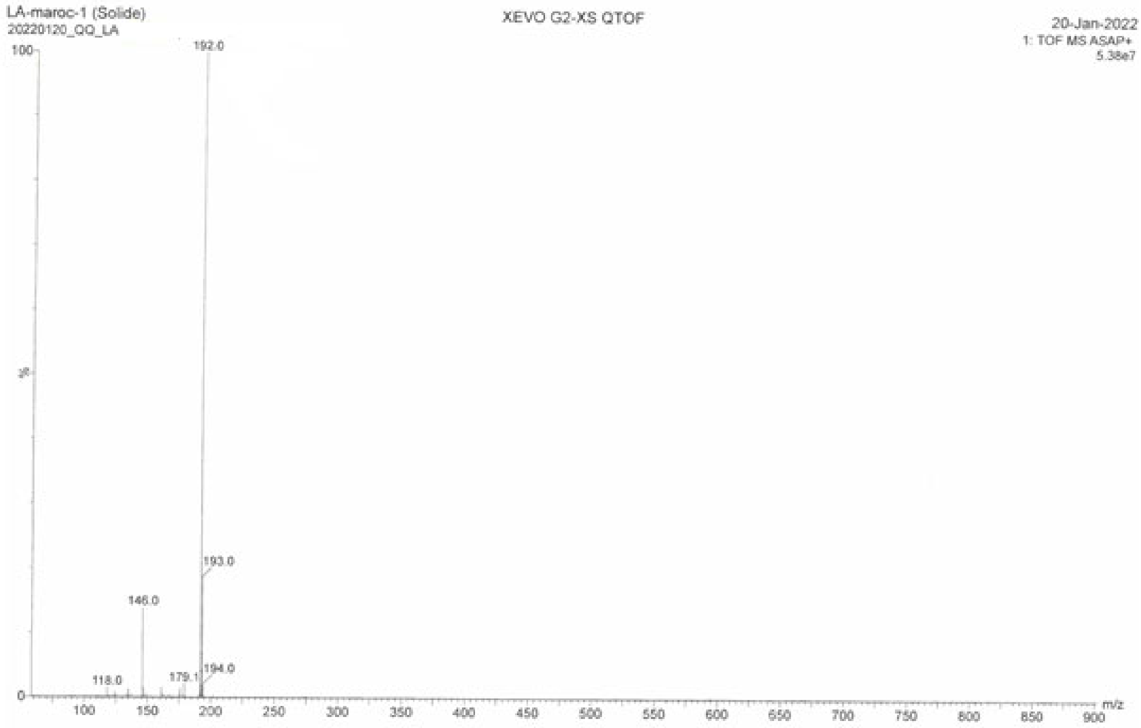


**Figure S4**. MS-ESI(+) spectrum of compound **2**

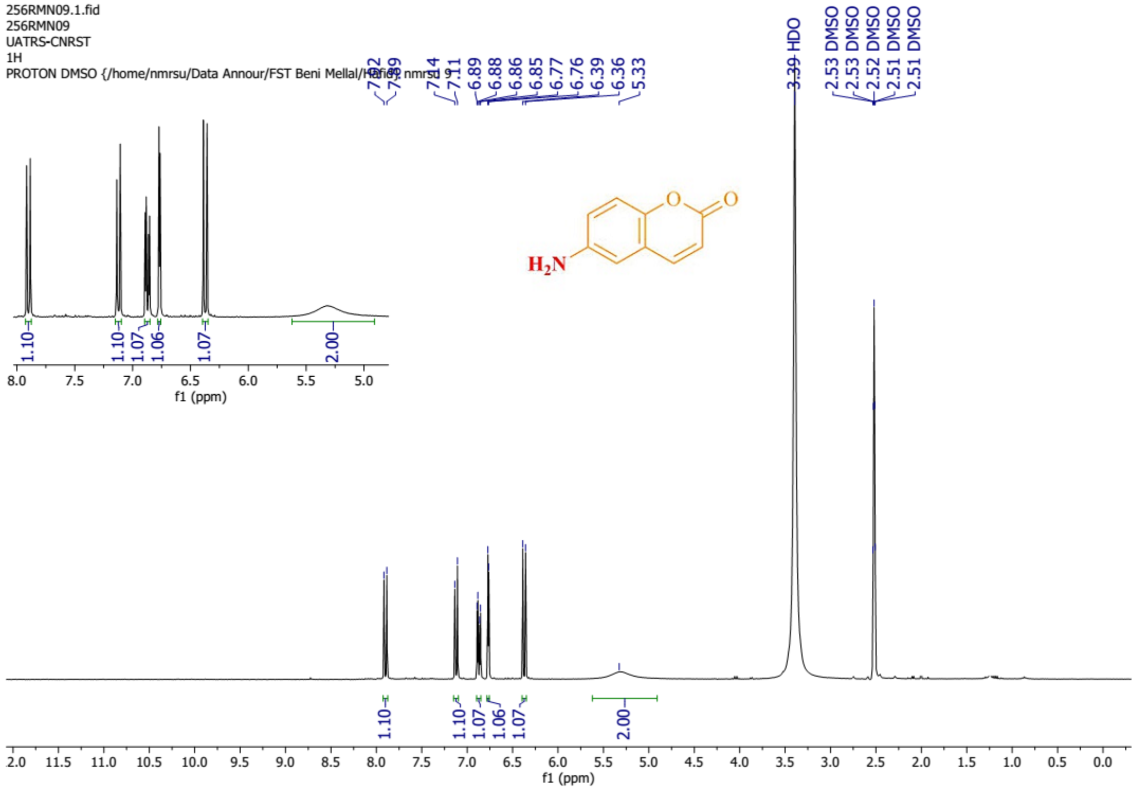


**Figure S5**. $^{1}$H NMR spectrum of compound **10a** in DMSO-$d_6$.

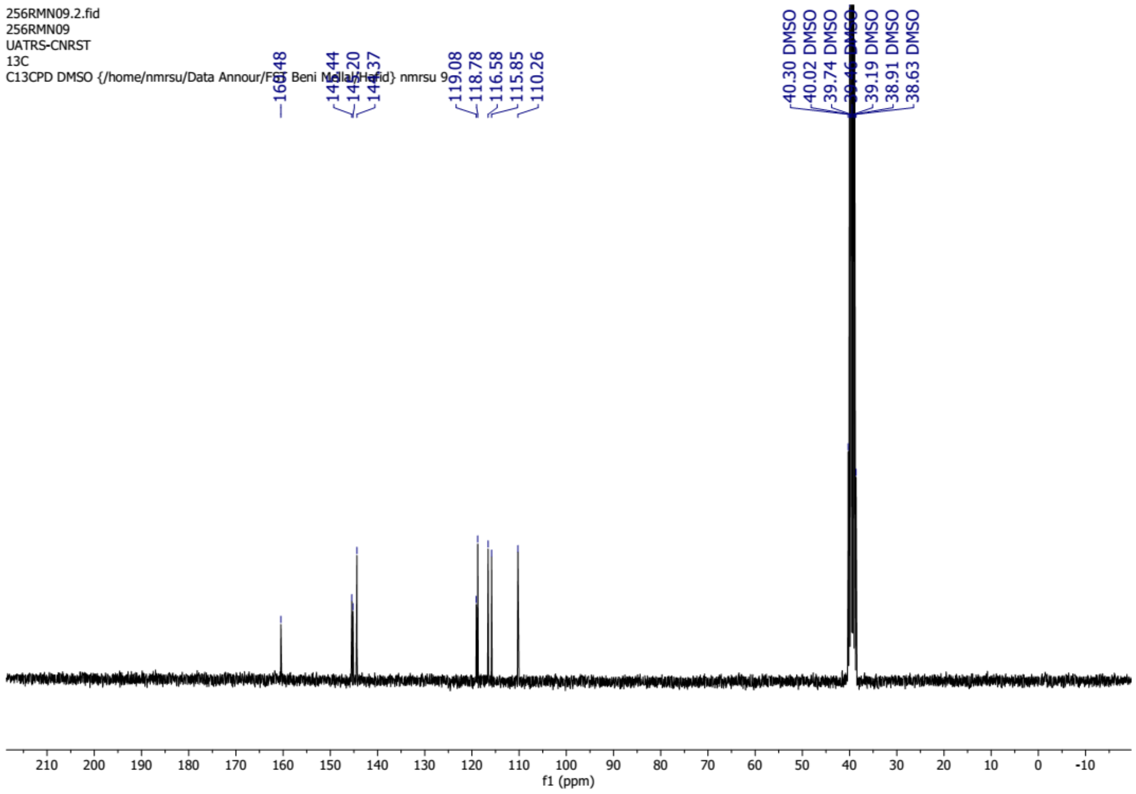


**Figure S6**. $^{13}$C NMR spectrum of compound **10a** in DMSO-$d_6$.

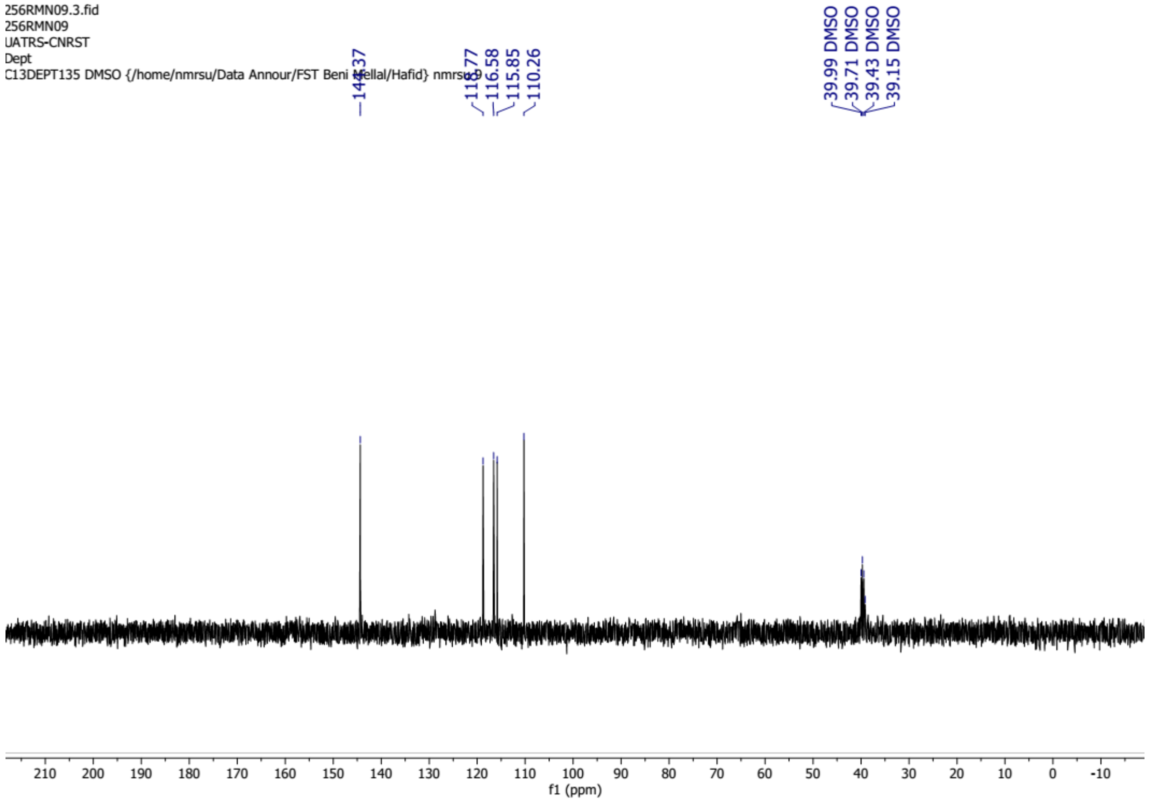


**Figure S7**. $^{13}$C NMR, DEPT 135 spectrum of compound **10a** in DMSO-*d*$_6$.

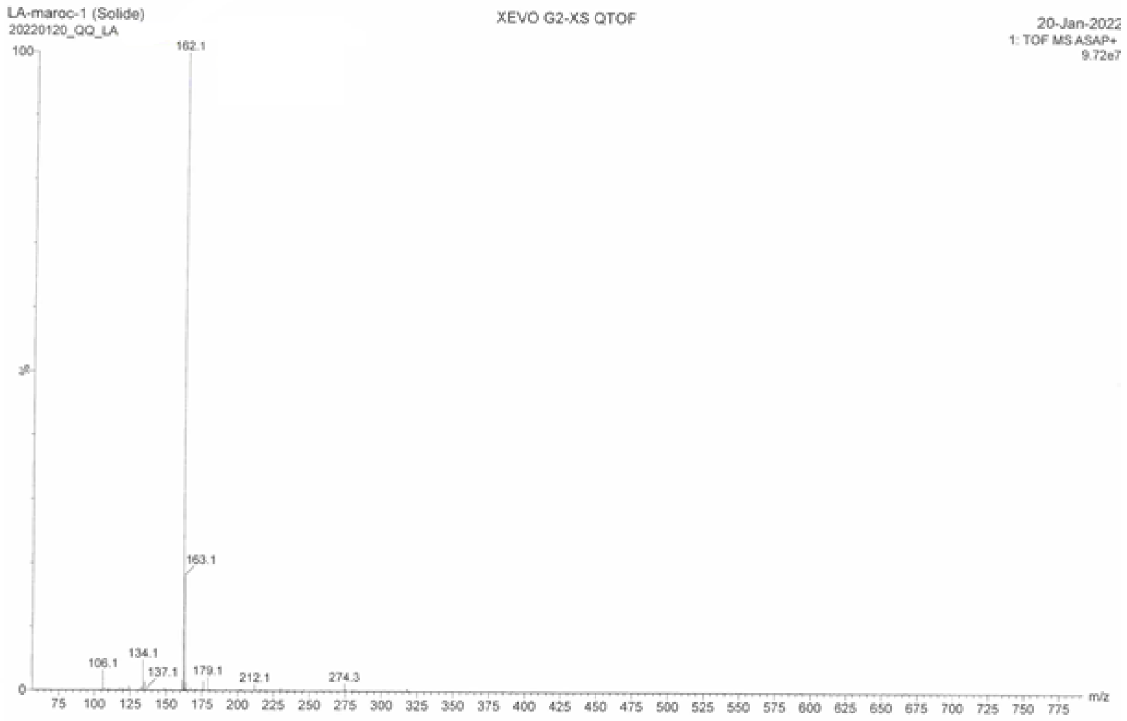


**Figure S8**. MS-ESI(+) spectrum of compound **10a**

## C. NMR and mass spectrum of compounds 11a-c

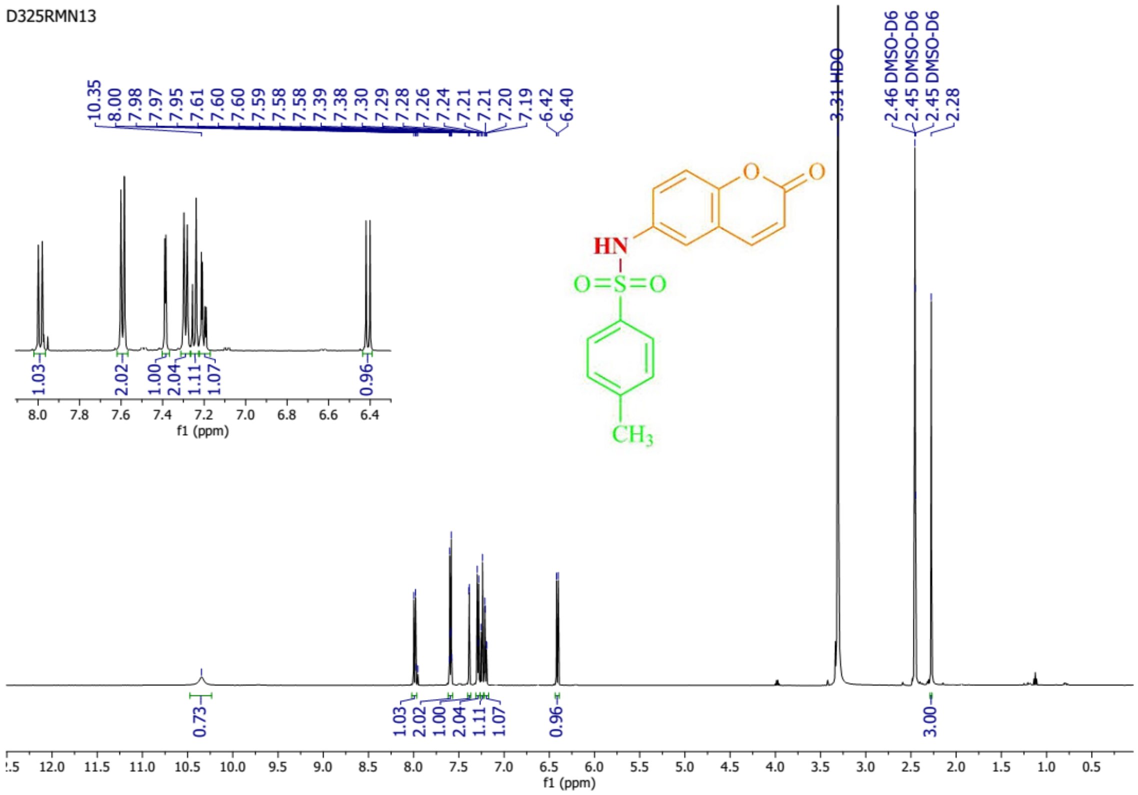


**Figure S9**. $^{1}$H NMR spectrum of compound **11a** in DMSO-$d_6$.

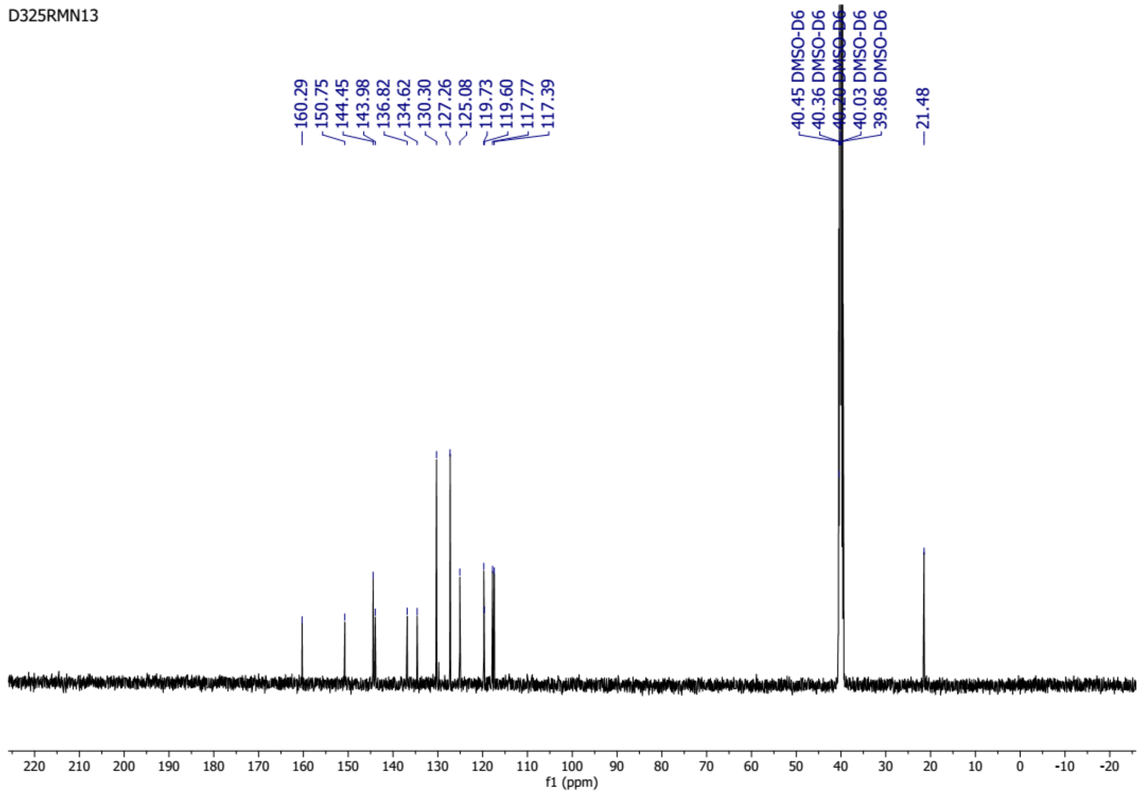


**Figure S10**. $^{13}$C NMR spectrum of compound **11a** in DMSO-$d_6$.

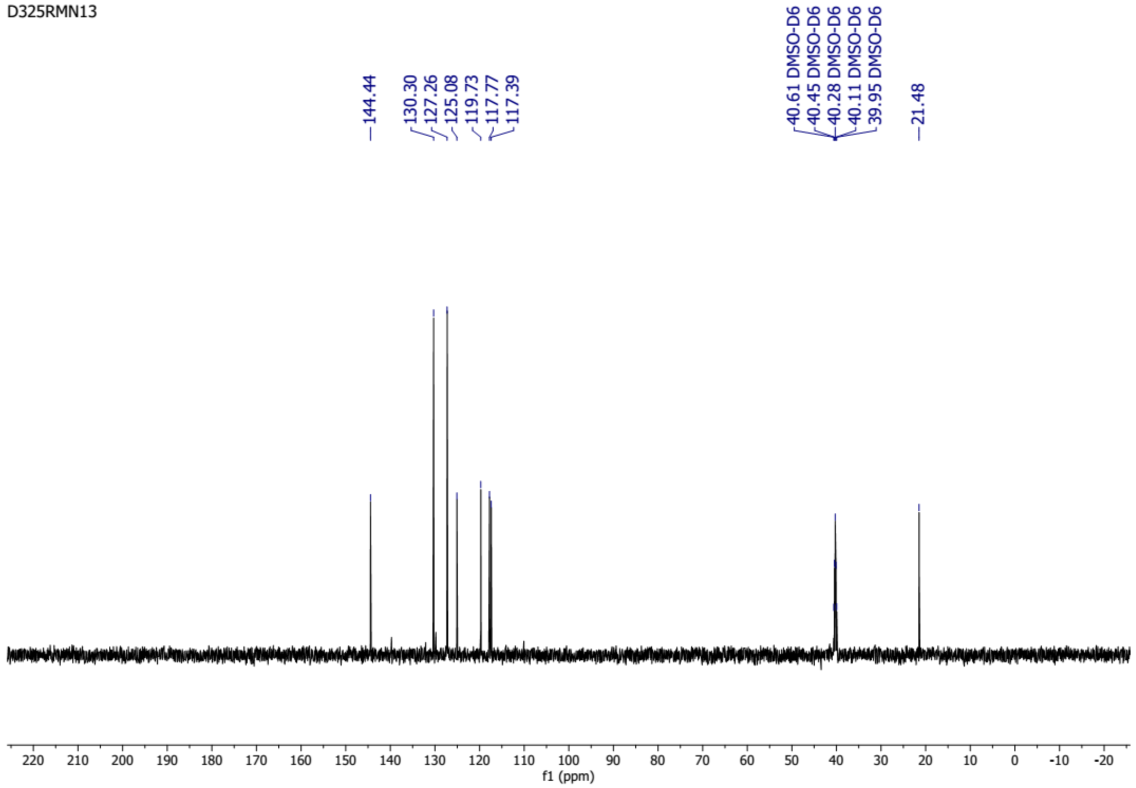


**Figure S11**. $^{13}$C NMR, DEPT 135 spectrum of compound **11a** in DMSO-$d_6$.

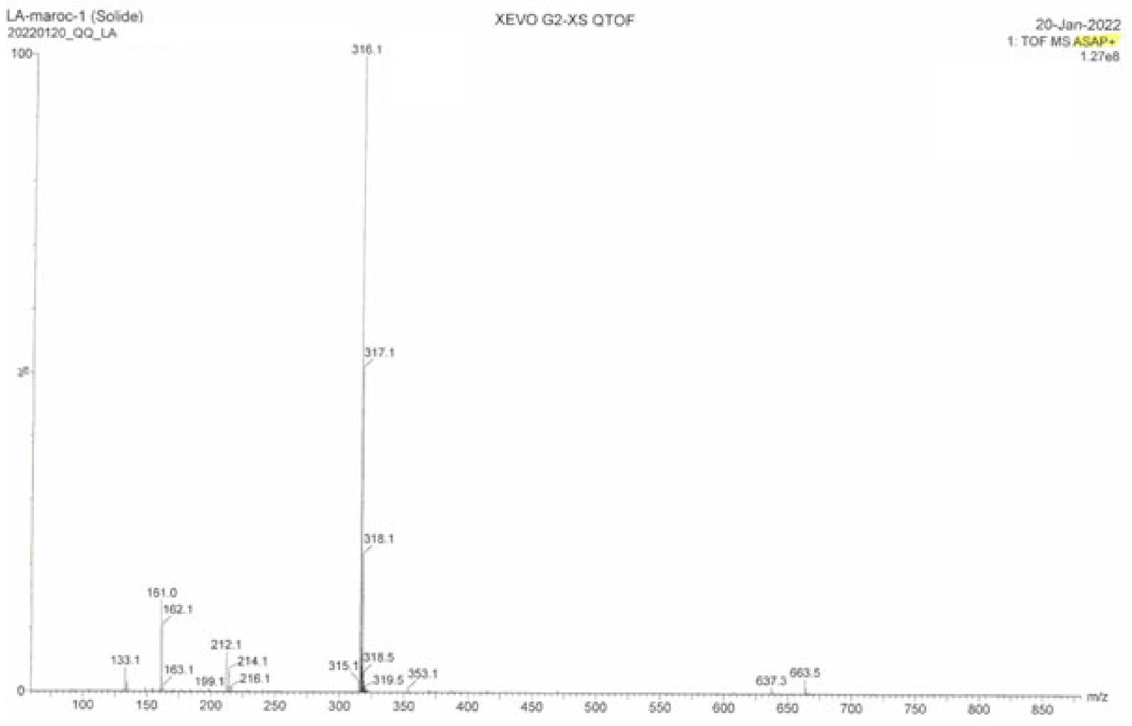


**Figure S12**. MS-ESI(+) spectrum of compound **11a**

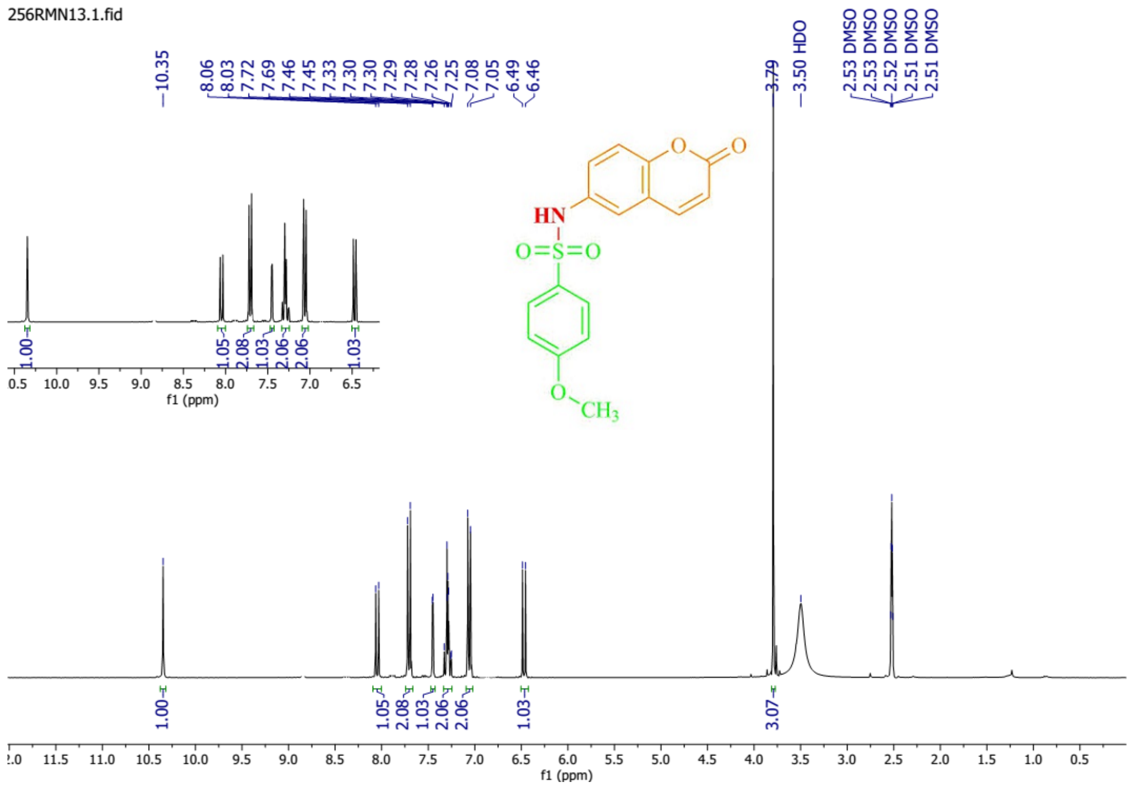


**Figure S13**. $^{1}$H NMR spectrum of compound **11b** in DMSO-*d*$_6$.

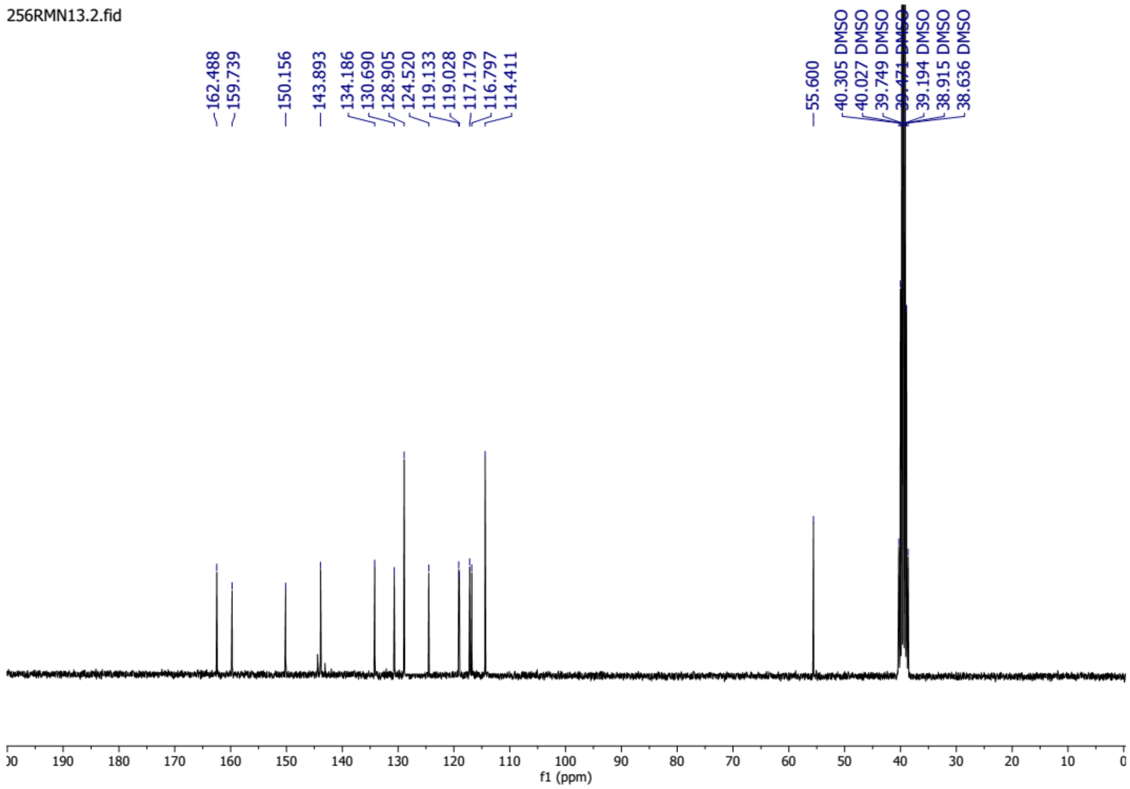


**Figure S14**. $^{13}$C NMR spectrum of compound **11b** in DMSO-*d*$_6$.

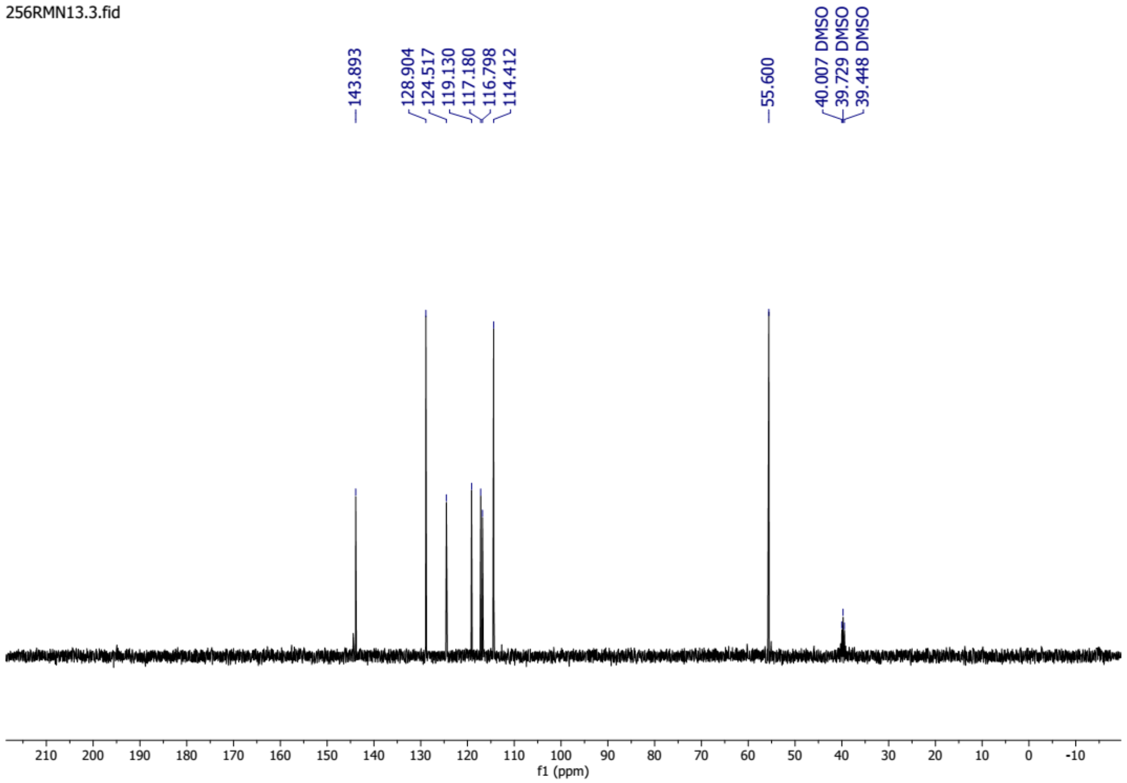


**Figure S15**. $^{13}C$ NMR, DEPT 135 spectrum of compound **11b** in DMSO-$d_6$.

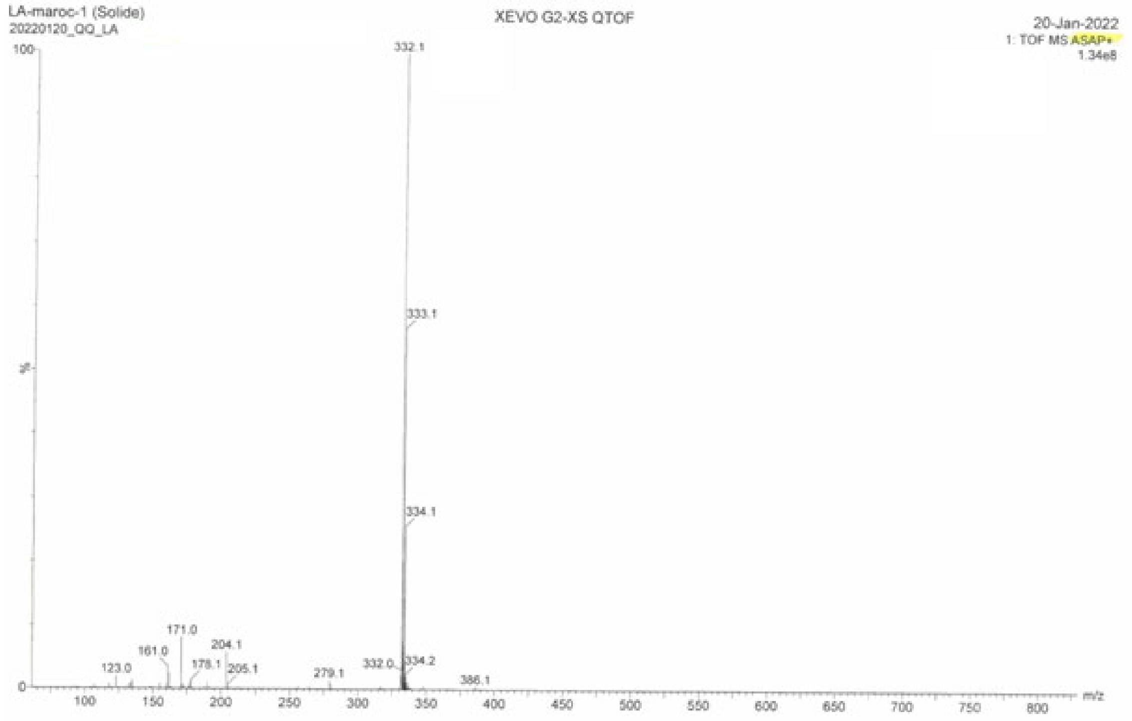


**Figure S16**. MS-ESI(+) spectrum of compound **11b**

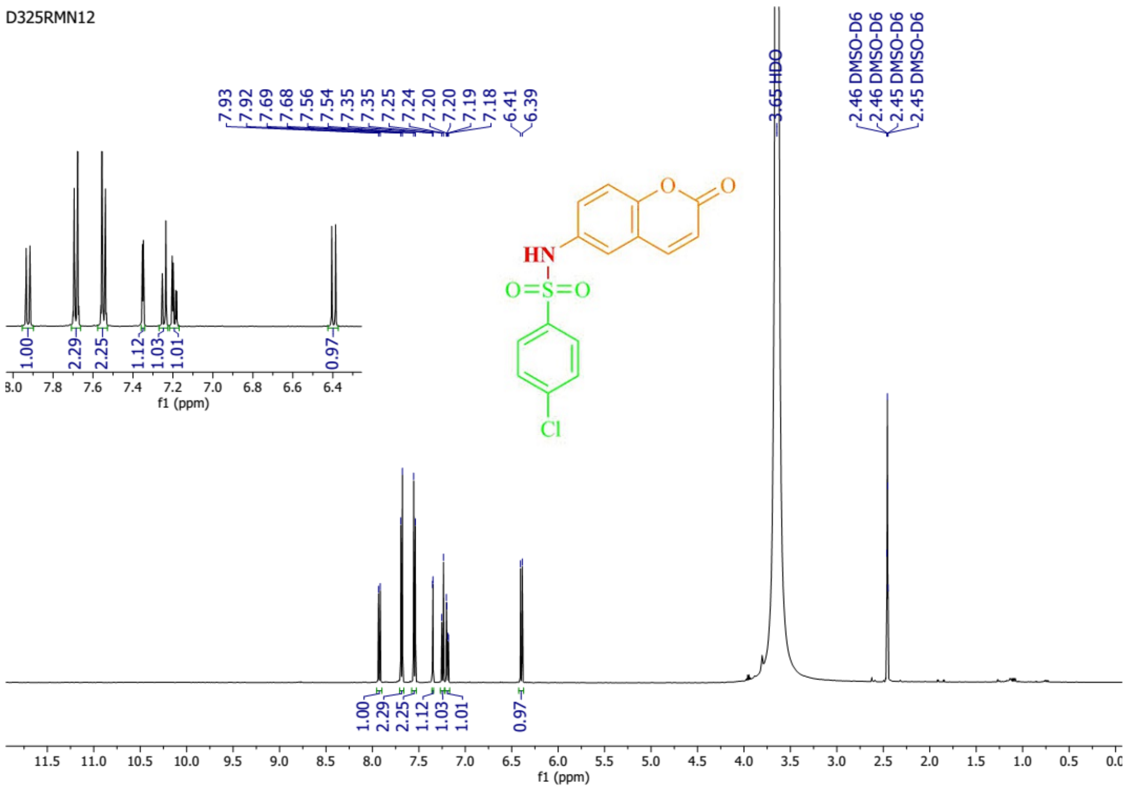


**Figure S17**. $^{1}$H NMR spectrum of compound **11c** in DMSO-$d_6$.

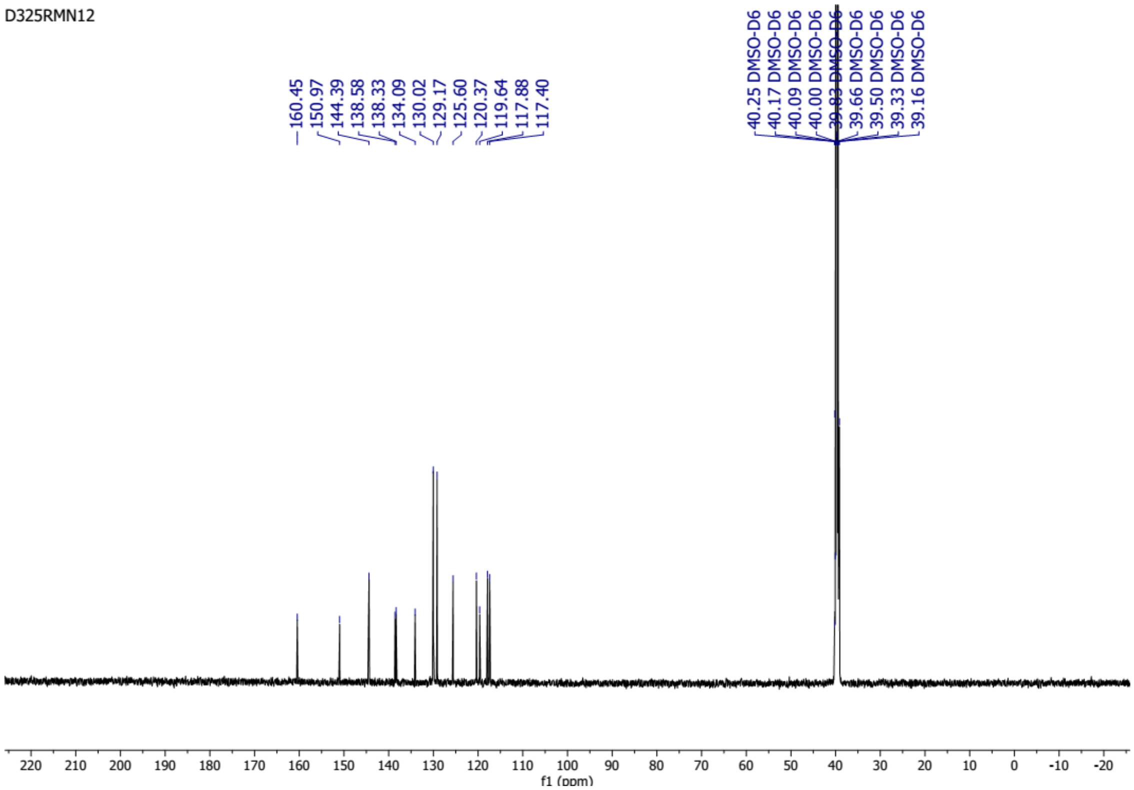


**Figure S18**. $^{13}$C NMR spectrum of compound **11c** in DMSO-$d_6$.

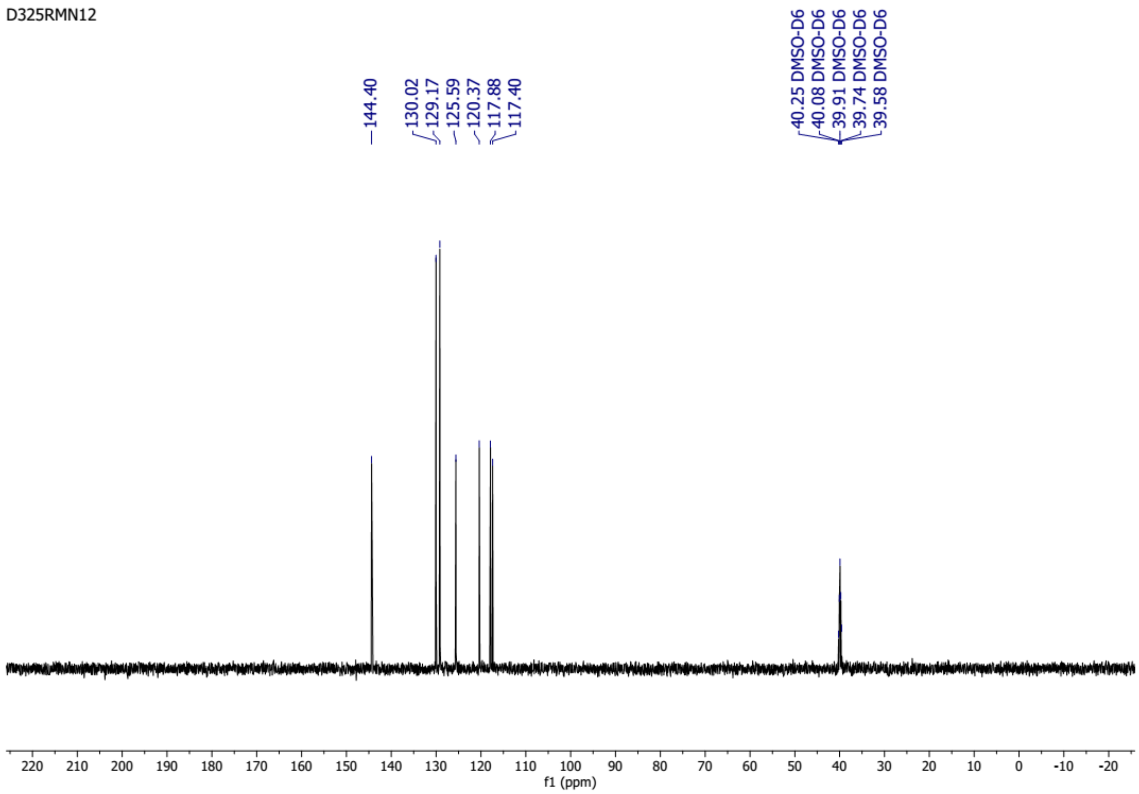


**Figure S19**. $^{13}$C NMR, DEPT 135 spectrum of compound **11c** in DMSO-$d_6$.

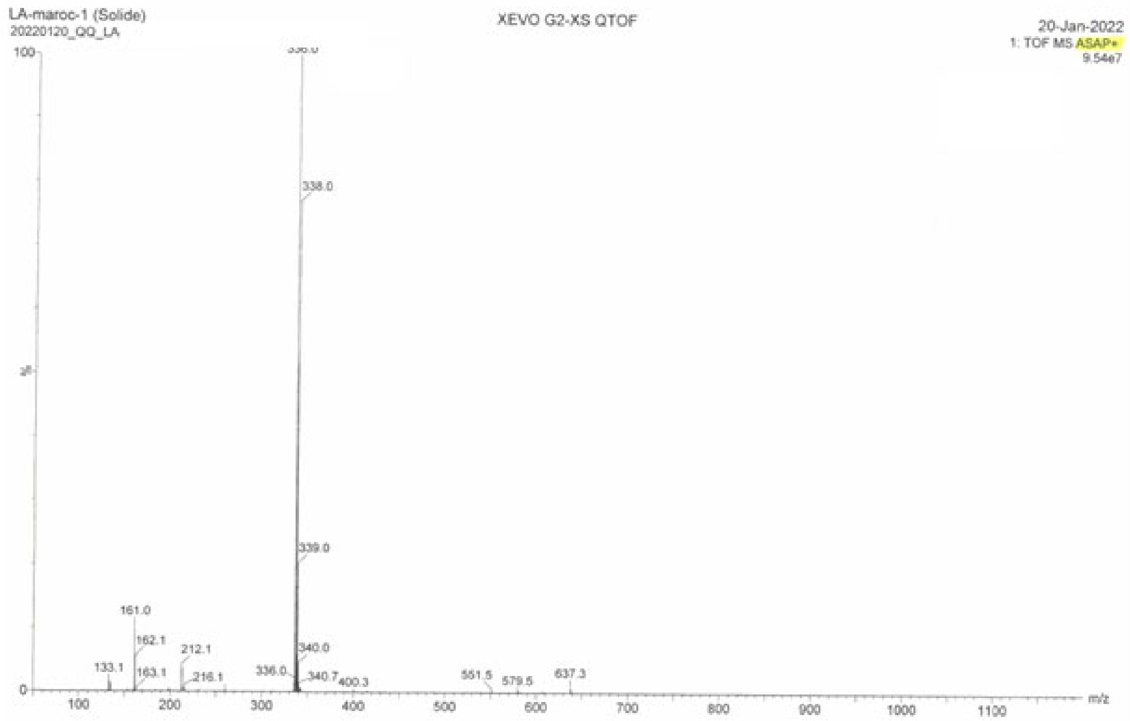


**Figure S20**. MS-ESI(+) spectrum of compound **11c**

**D. NMR and mass spectrum of compounds 12a and 13a-c**

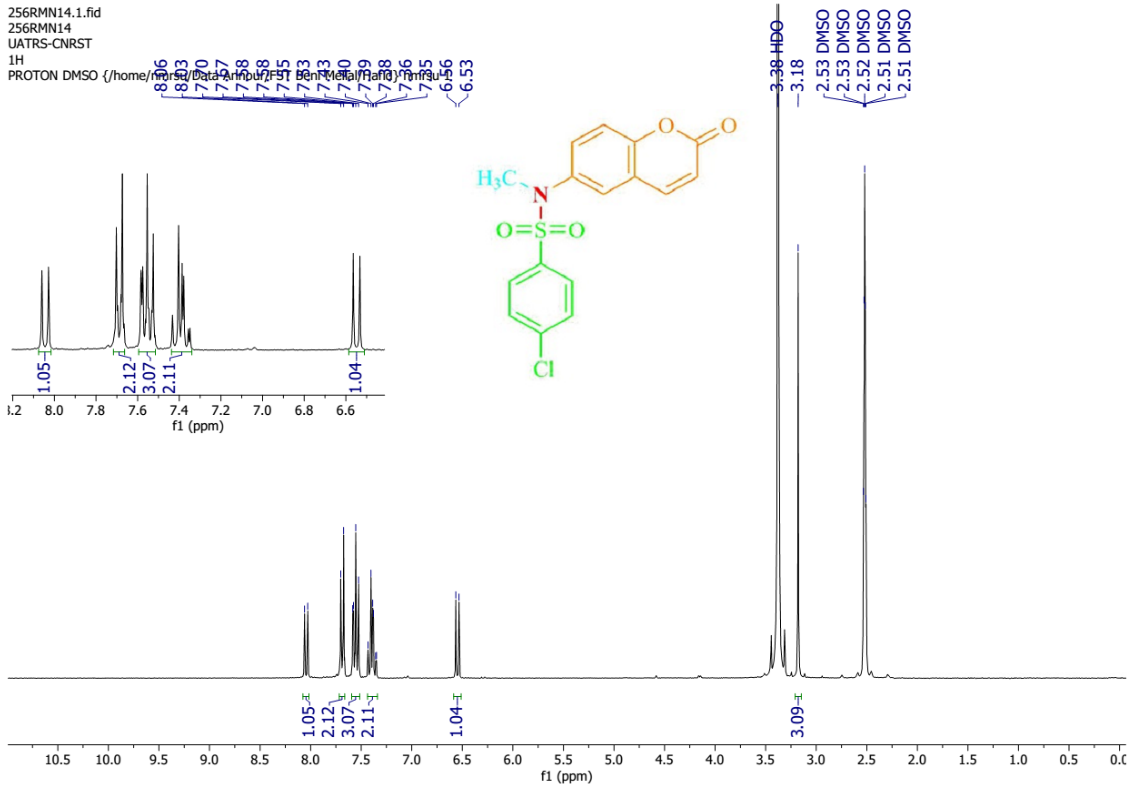


**Figure S21**. $^{1}$H NMR spectrum of compound **12a** in DMSO-*d*$_6$.

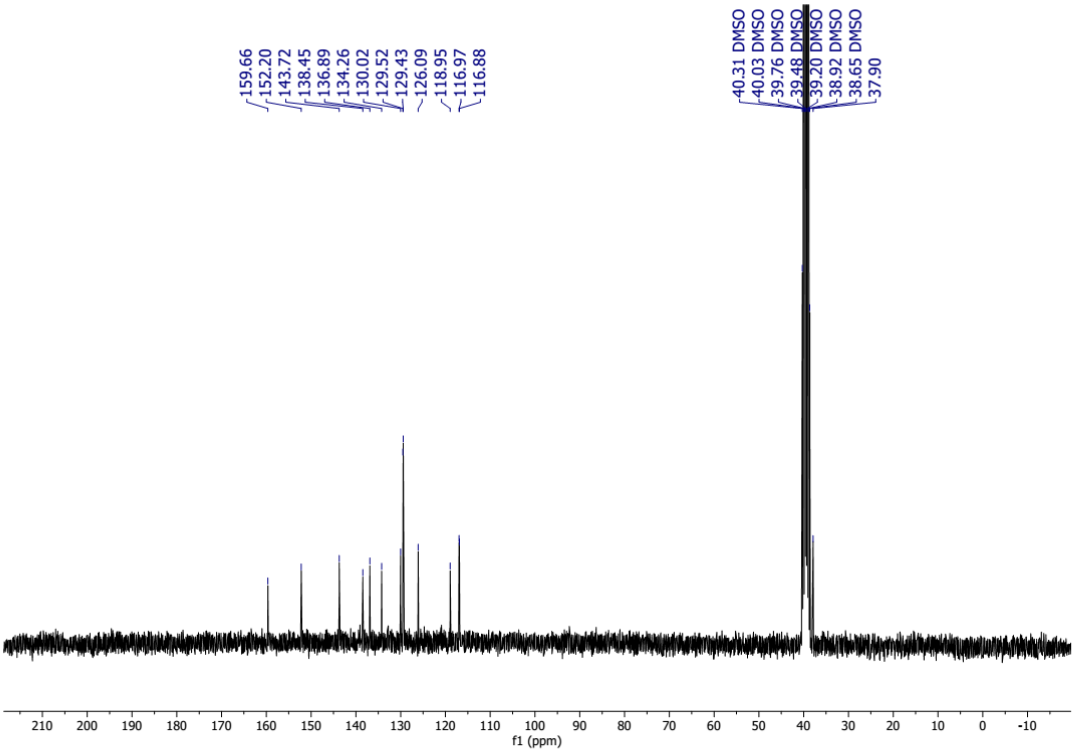


**Figure S22**. $^{13}$C NMR spectrum of compound **12a** in DMSO-*d*$_6$.

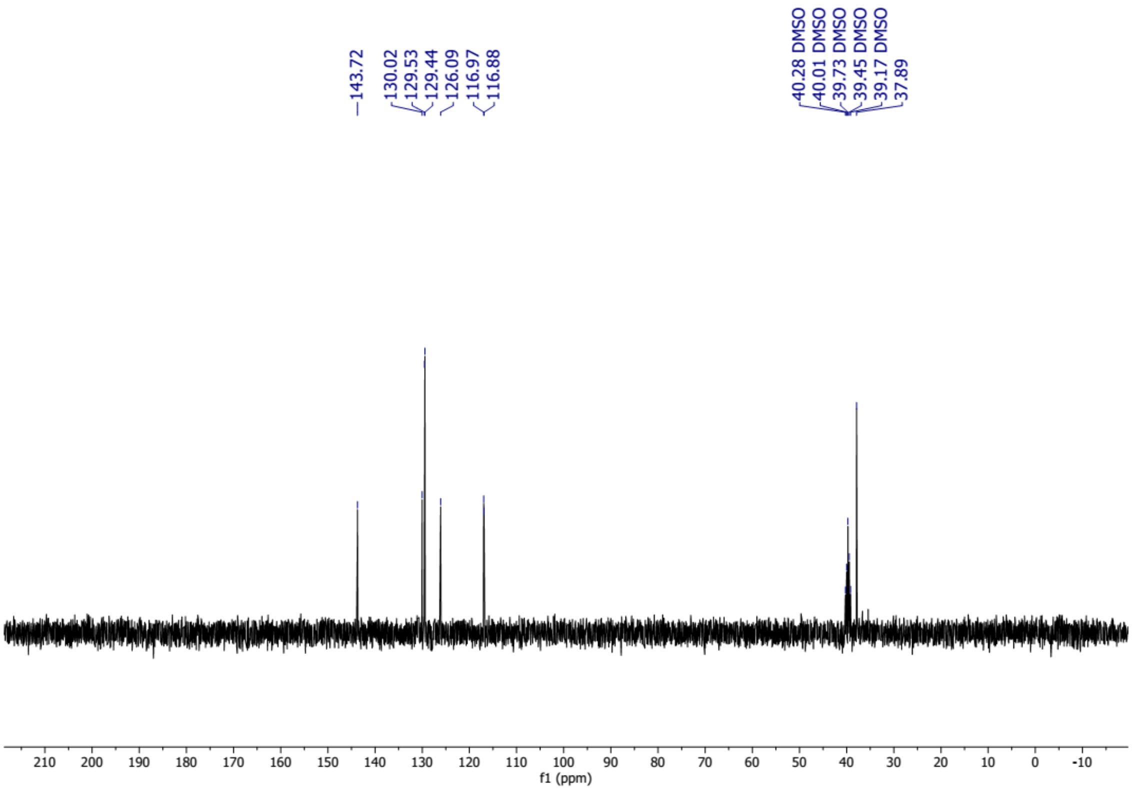


**Figure S23**. $^{13}$C NMR, DEPT 135 spectrum of compound **12a** in DMSO-$d_6$.

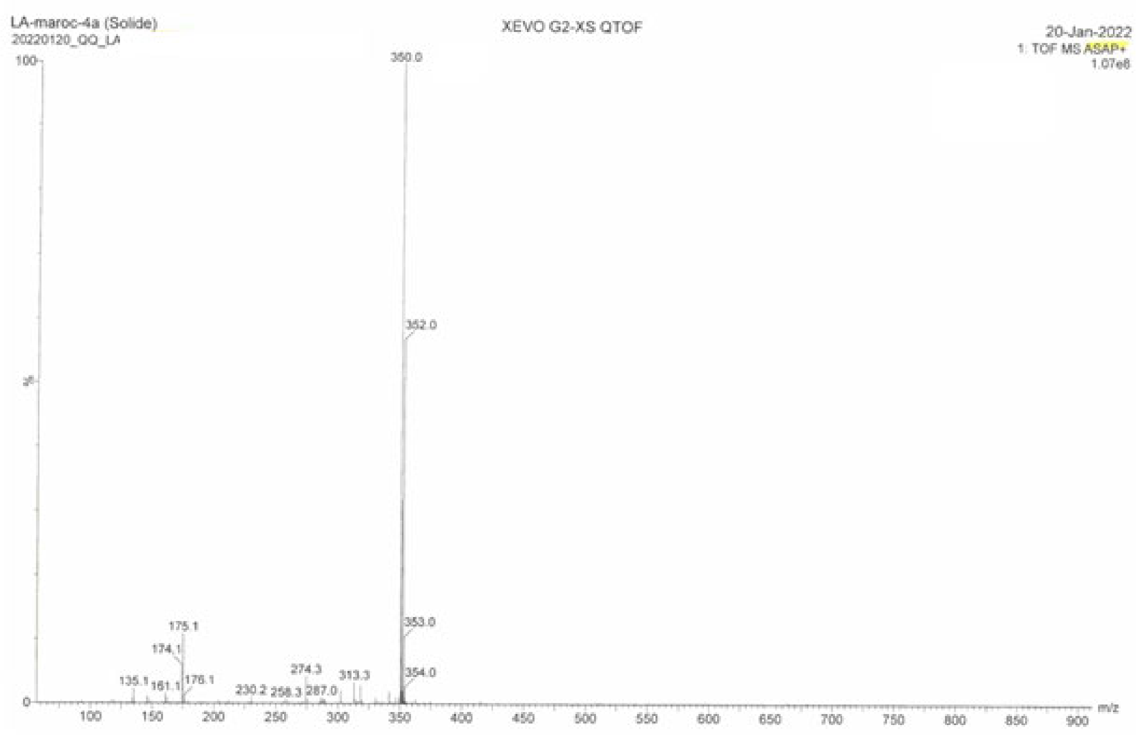


**Figure S24**. MS-ESI(+) spectrum of compound **12a**

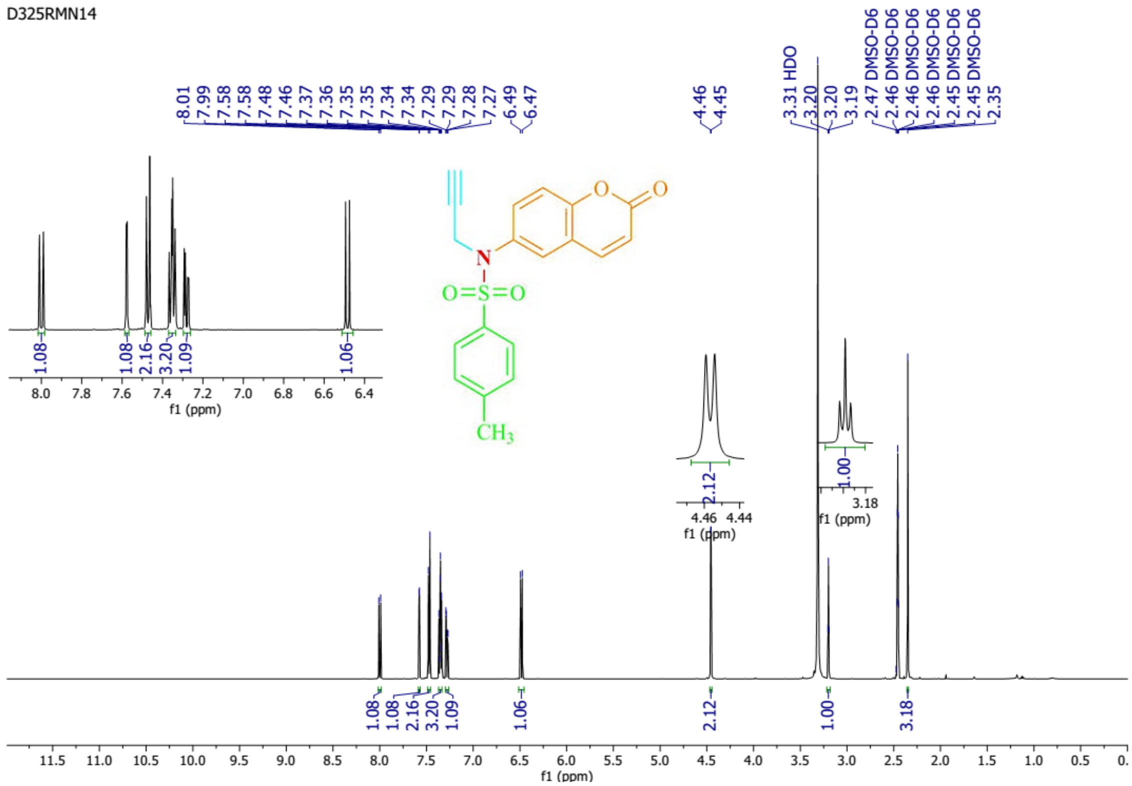


**Figure S25**. $^{1}H$ NMR spectrum of compound **13a** in DMSO-*d*$_6$.

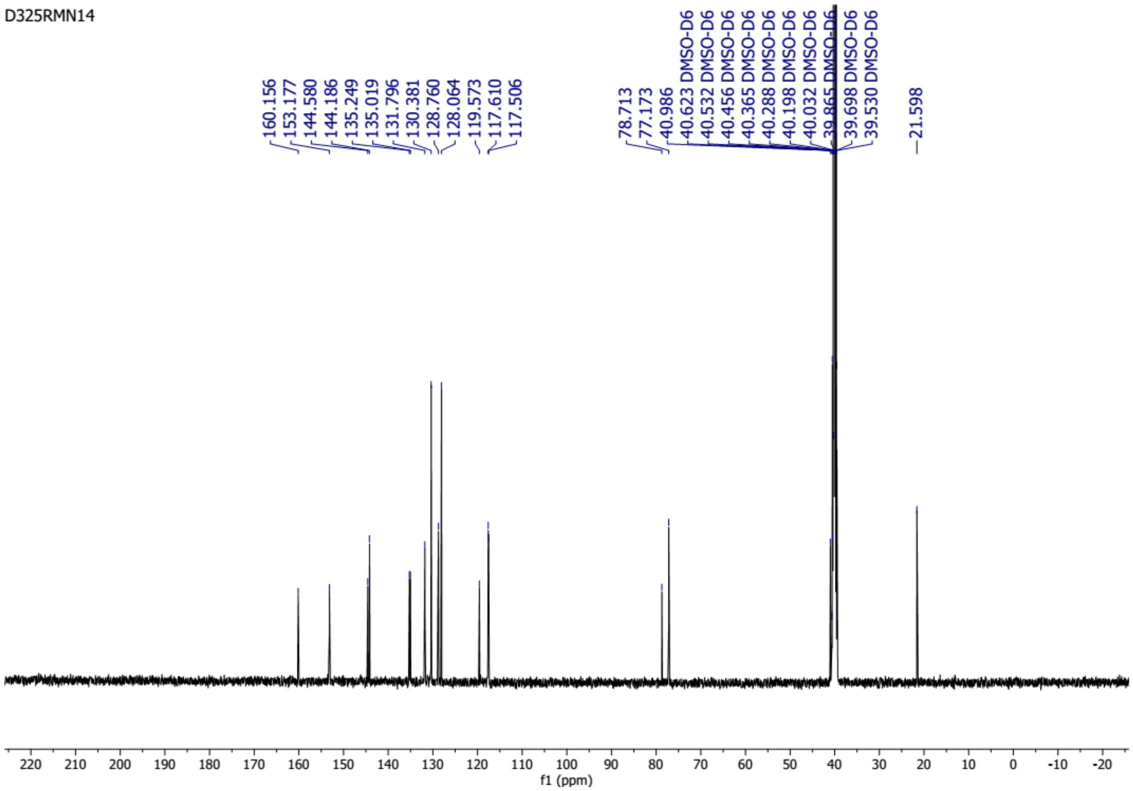


**Figure S26**. $^{13}C$ NMR spectrum of compound **13a** in DMSO-*d*$_6$.

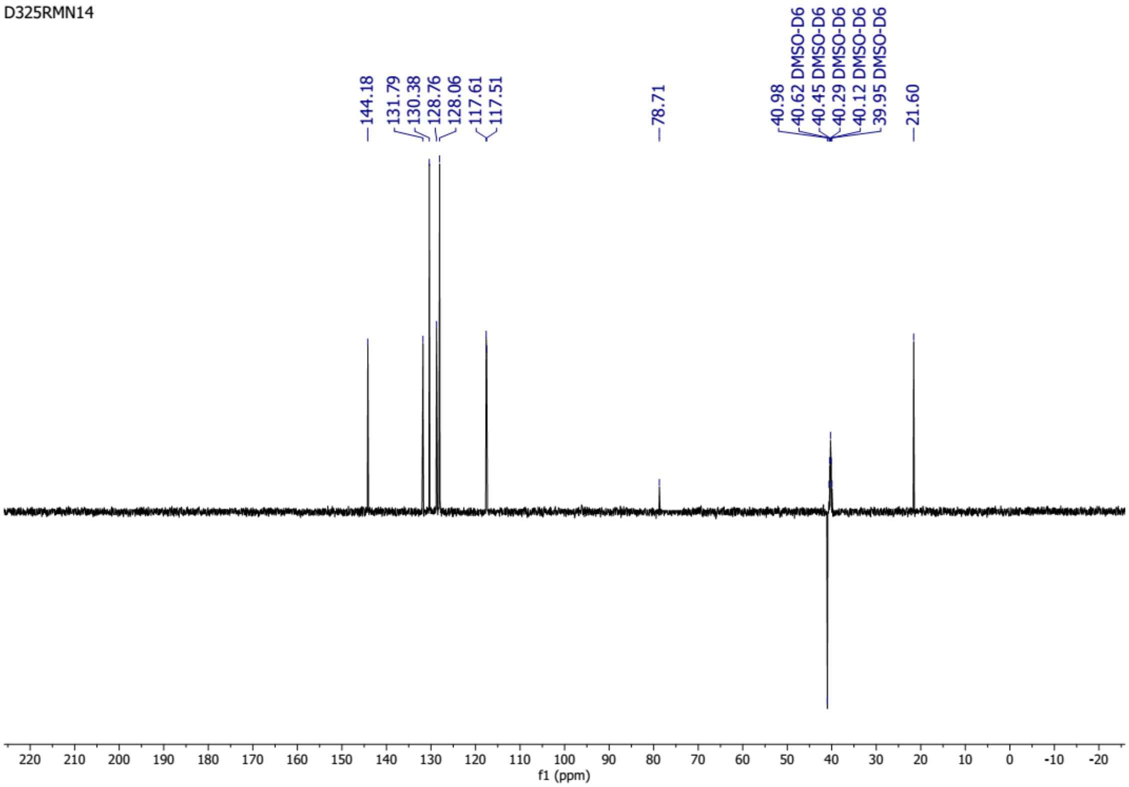


**Figure S27**. $^{13}C$ NMR, DEPT 135 spectrum of compound **13a** in DMSO-$d_6$.

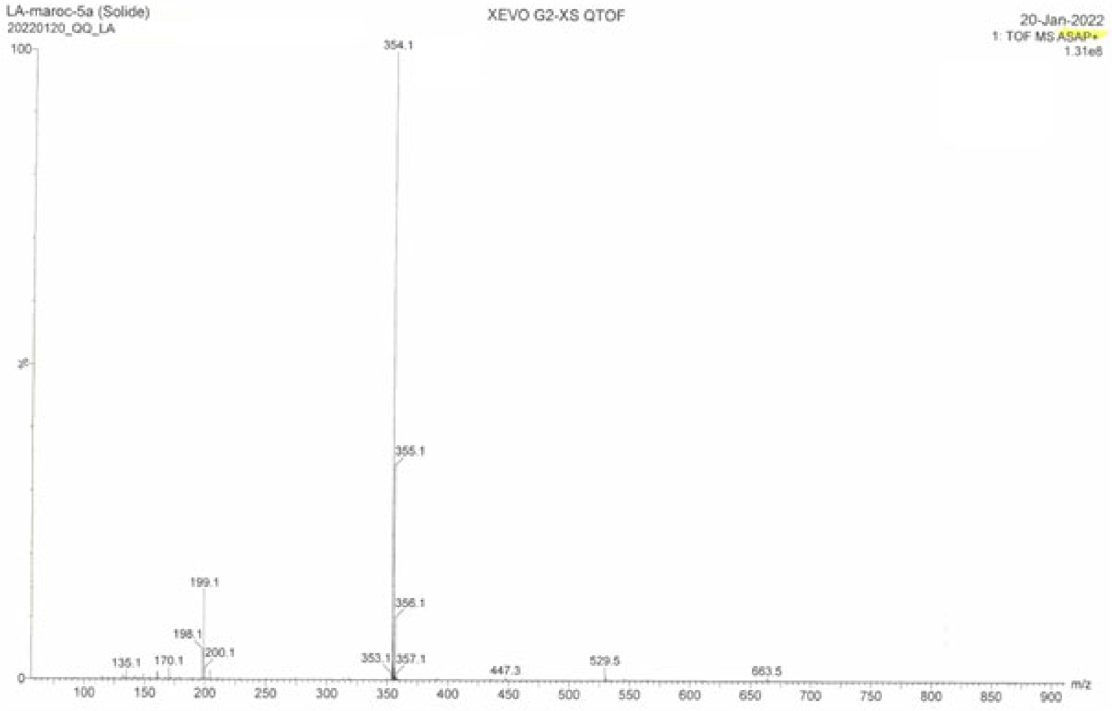


**Figure S28**. MS-ESI(+) spectrum of compound **13a**

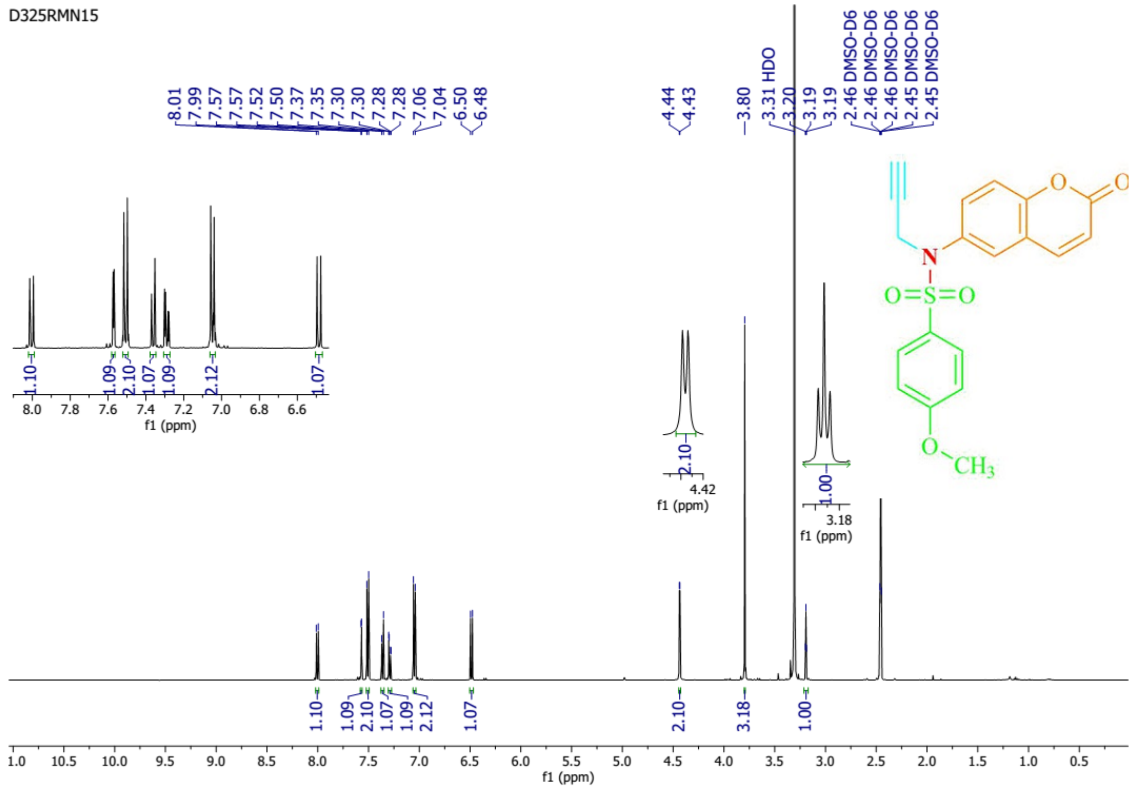


**Figure S29**. $^{1}$H NMR spectrum of compound **13b** in DMSO-$d_6$.

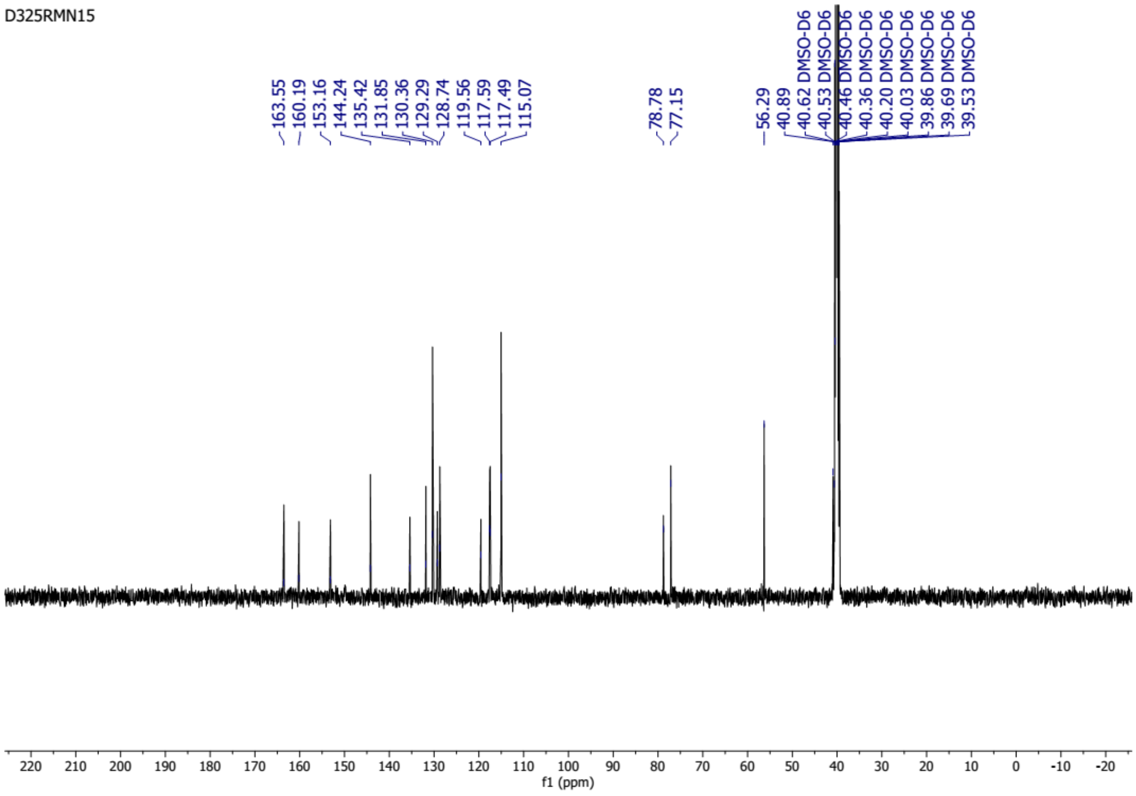


**Figure S30**. $^{13}$C NMR spectrum of compound **13b** in DMSO-$d_6$.

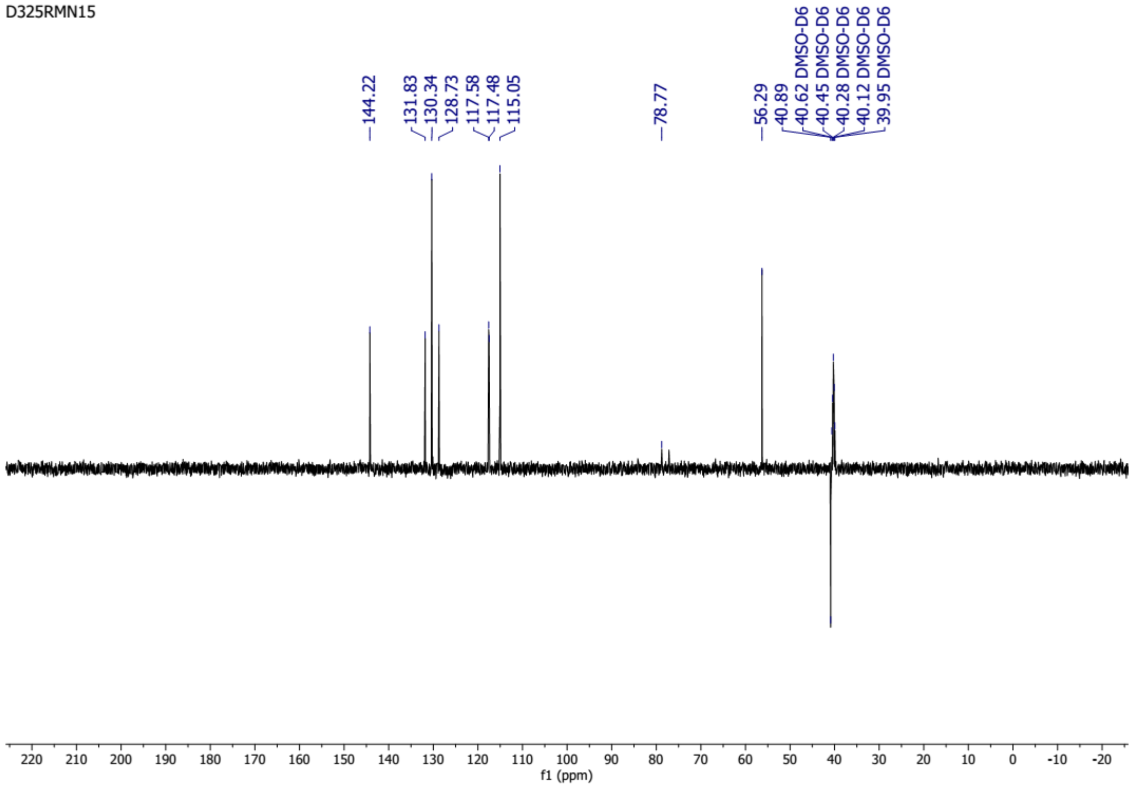


**Figure S31**. $^{13}C$ NMR, DEPT 135 spectrum of compound **13b** in DMSO-$d_6$.

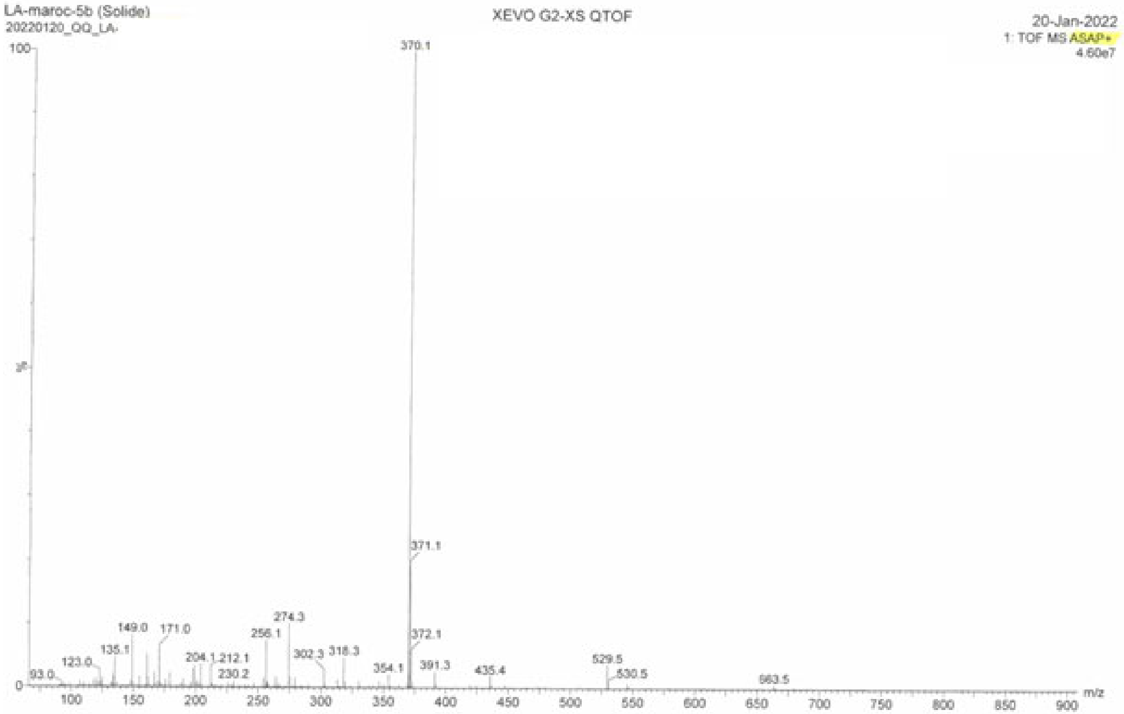


**Figure S32**. MS-ESI(+) spectrum of compound **13b**

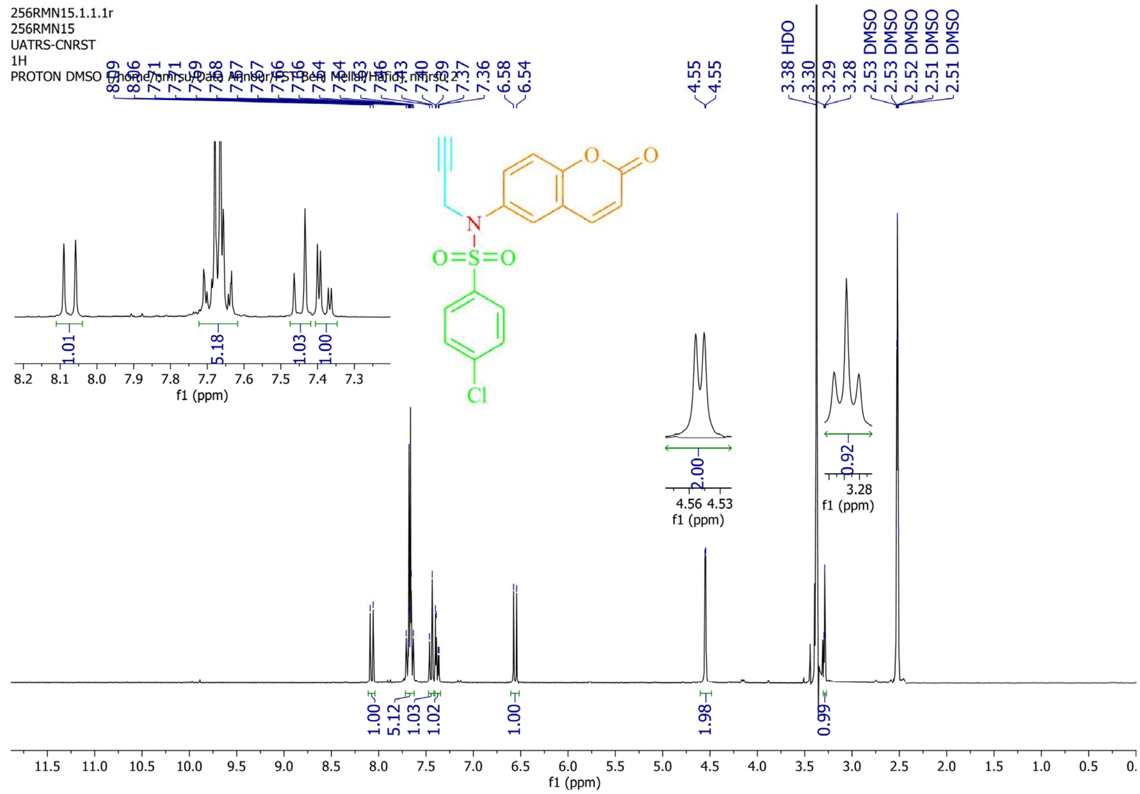

**Figure S33**. $^{1}$H NMR spectrum of compound **13c** in DMSO-$d_6$.

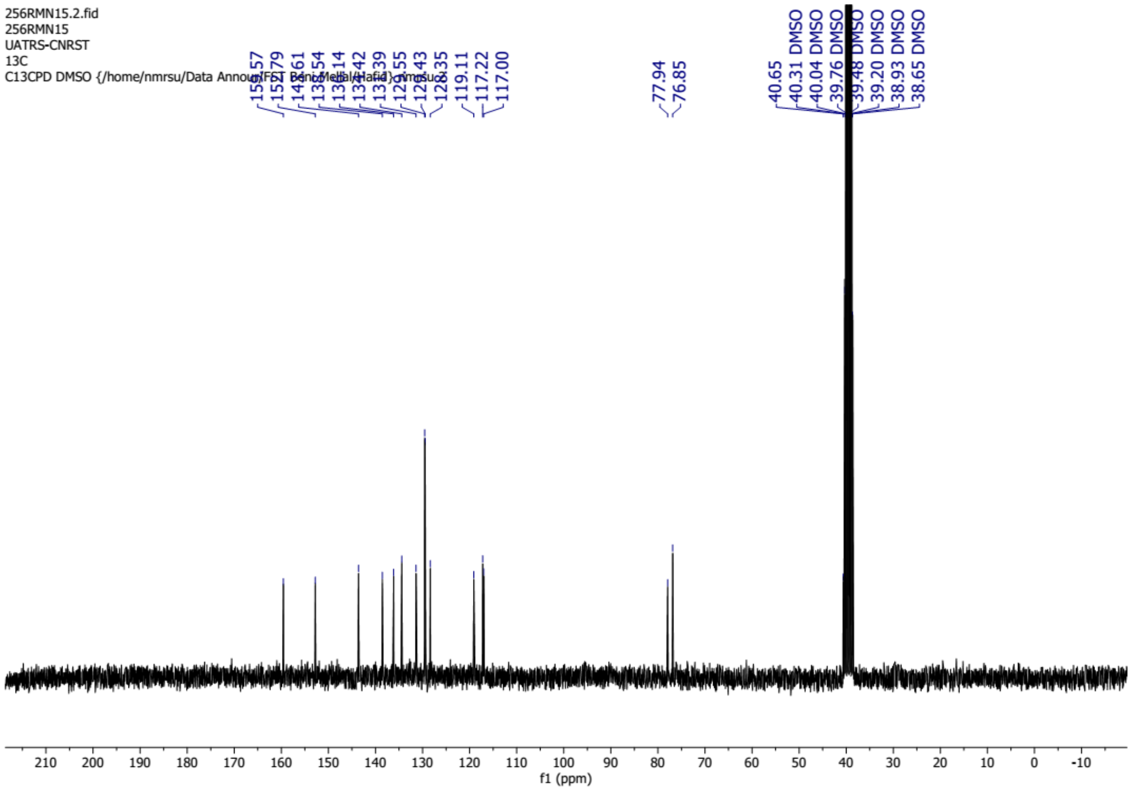

**Figure S34**. $^{13}$C NMR spectrum of compound **13c** in DMSO-$d_6$.

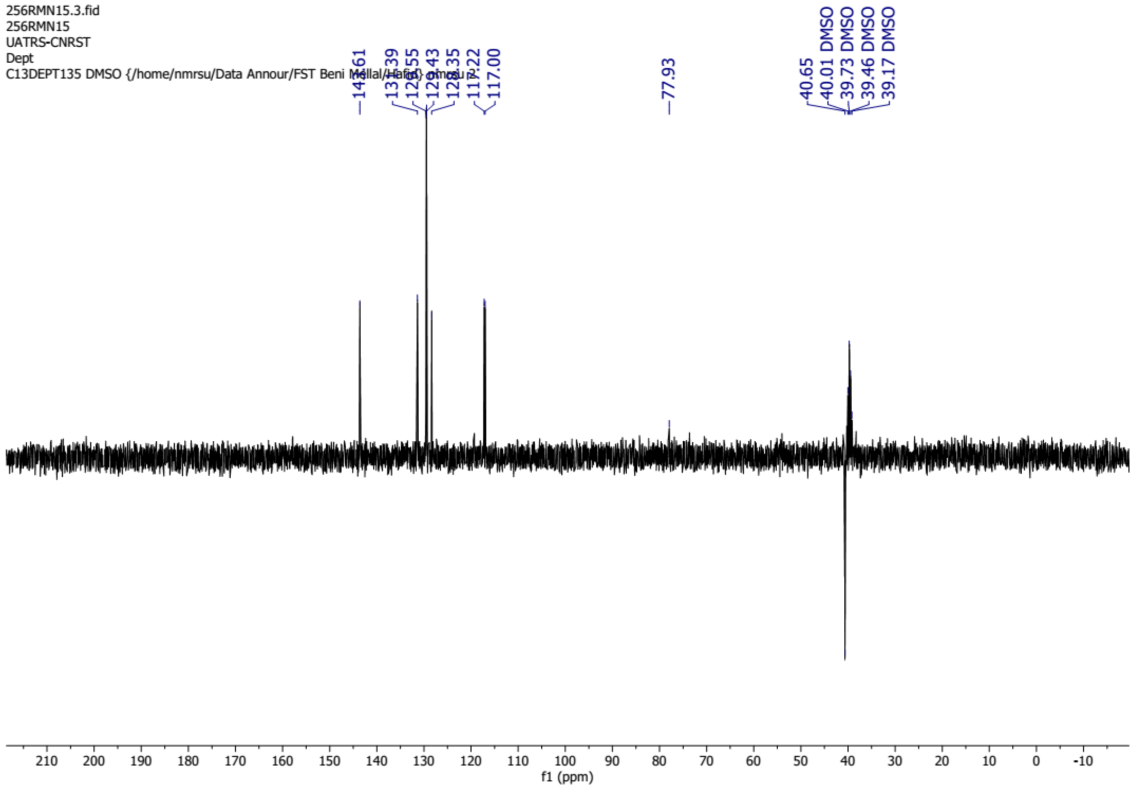


**Figure S35**. $^{13}$C NMR, DEPT 135 spectrum of compound **13c** in DMSO-$d_6$.

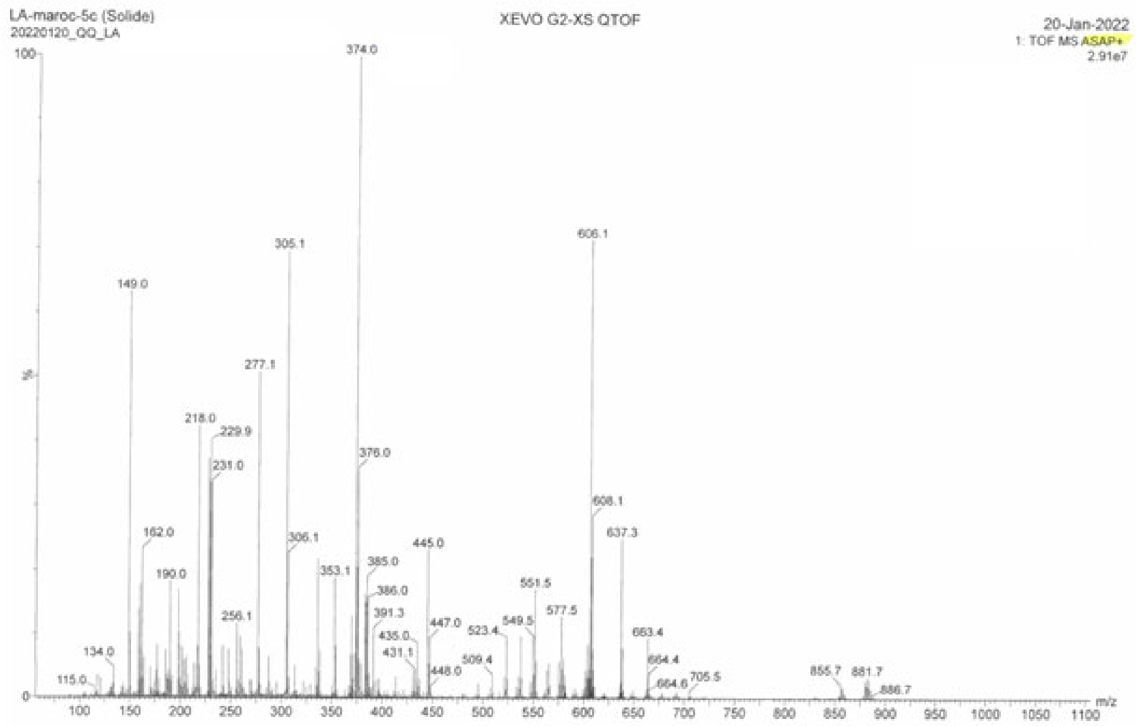


**Figure S36**. MS-ESI(+) spectrum of compound **13c**

## E. NMR and mass spectrum of compounds 14a-c

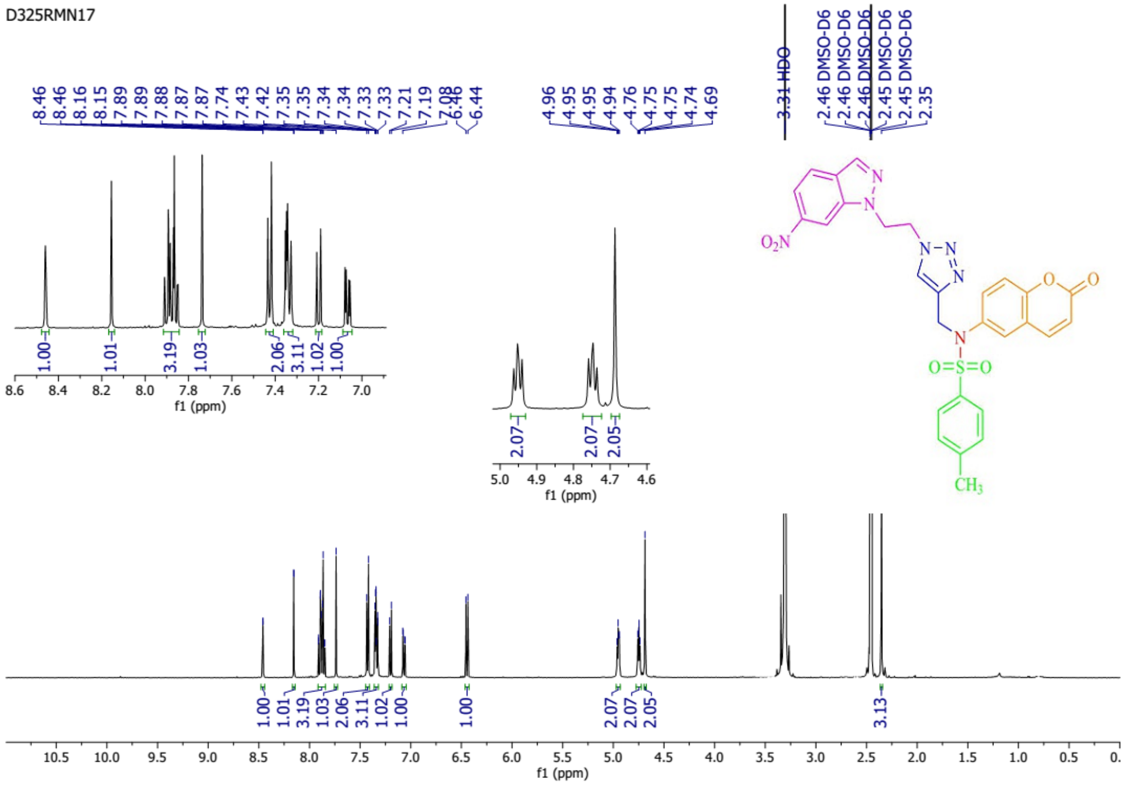


**Figure S37**. $^{1}$H NMR spectrum of compound **14a** in DMSO-$d_6$.

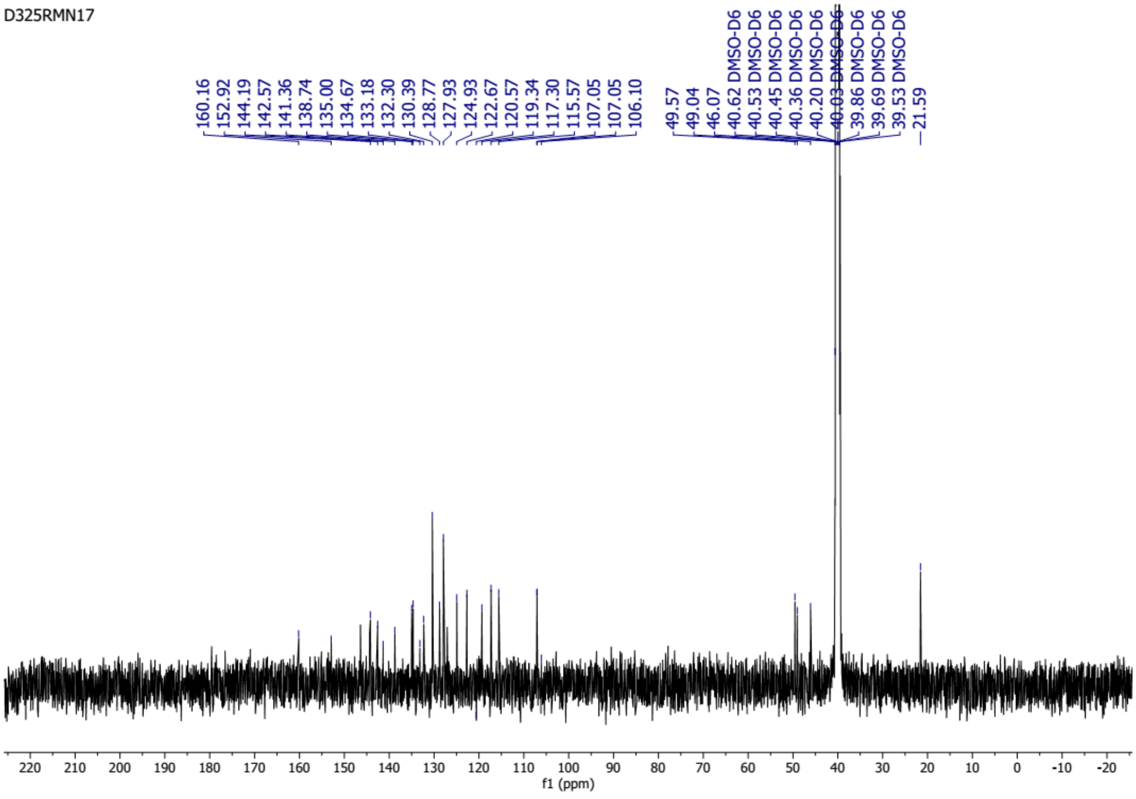


**Figure S38**. $^{13}$C NMR spectrum of compound **14a** in DMSO-$d_6$.

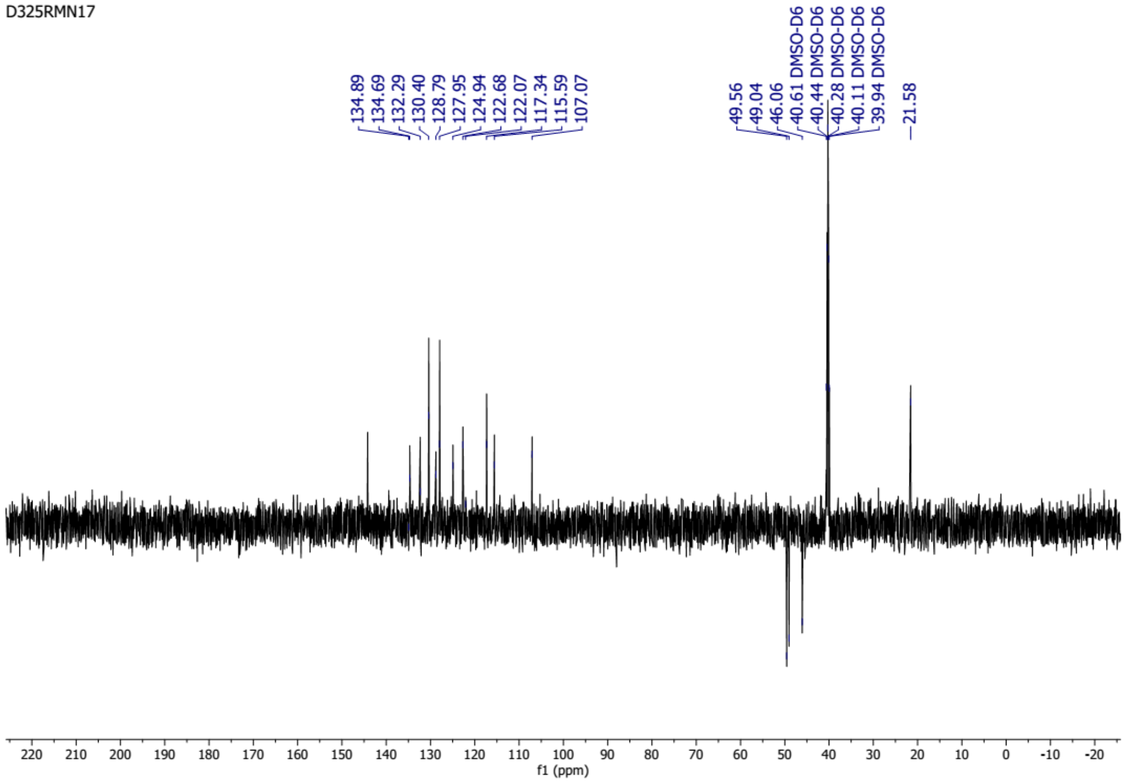


**Figure S39**. $^{13}$C NMR, DEPT 135 spectrum of compound **14a** in DMSO-$d_6$.

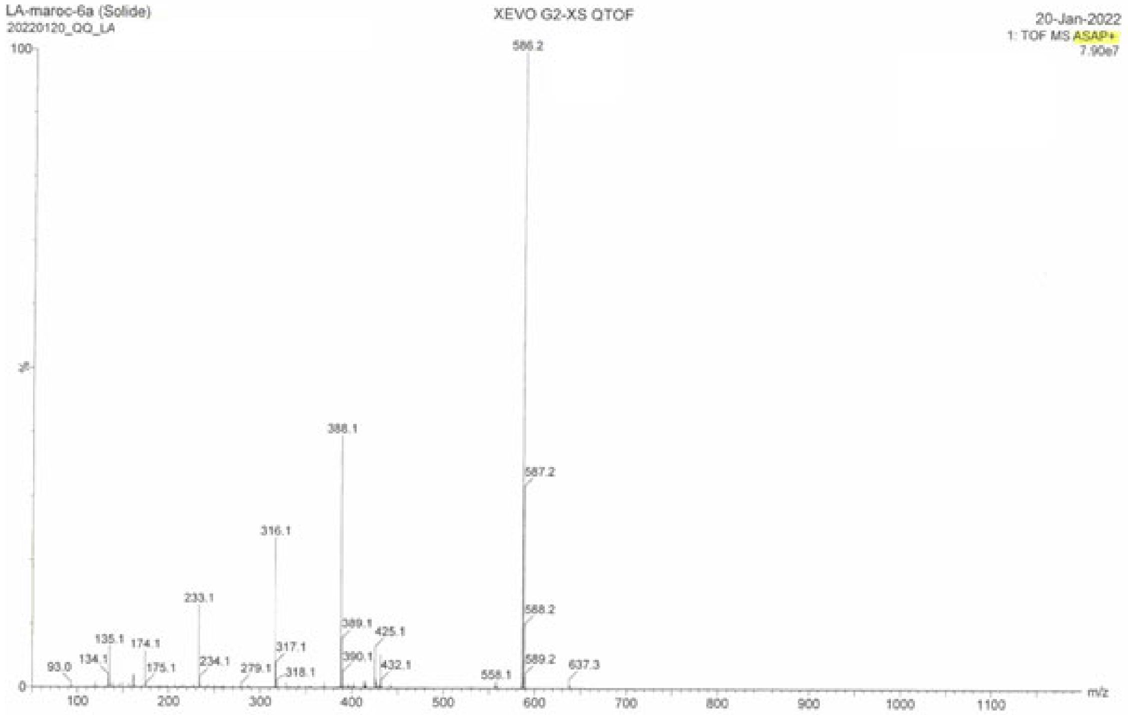


**Figure S40**. MS-ESI(+) spectrum of compound **14a**

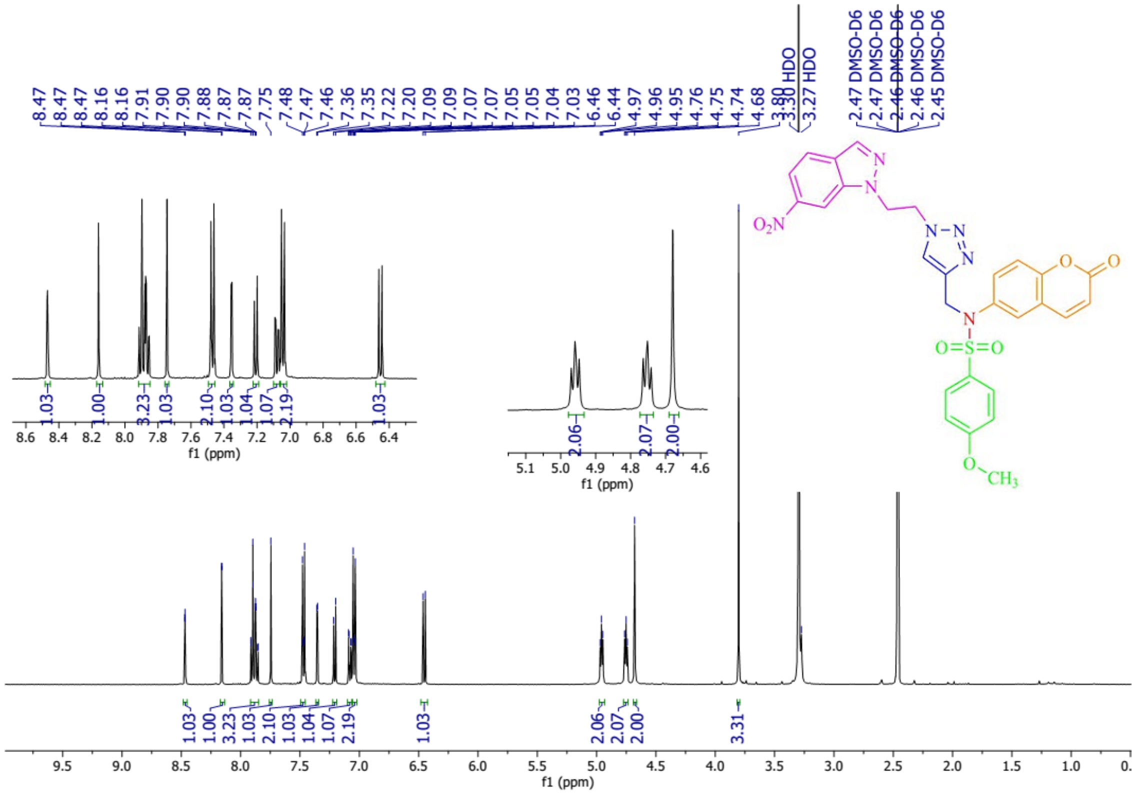

**Figure S41**. $^{1}$H NMR spectrum of compound **14b** in DMSO-$d_6$.

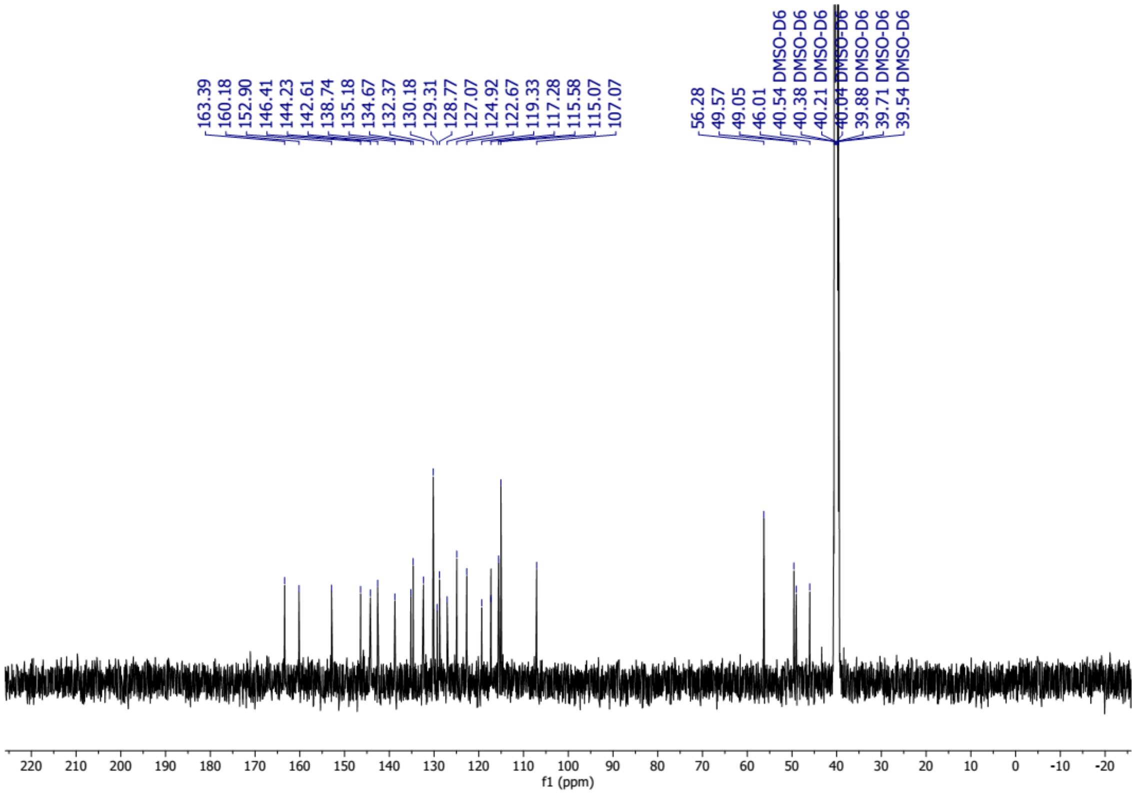

**Figure S42**. $^{13}$C NMR spectrum of compound **14b** in DMSO-$d_6$.

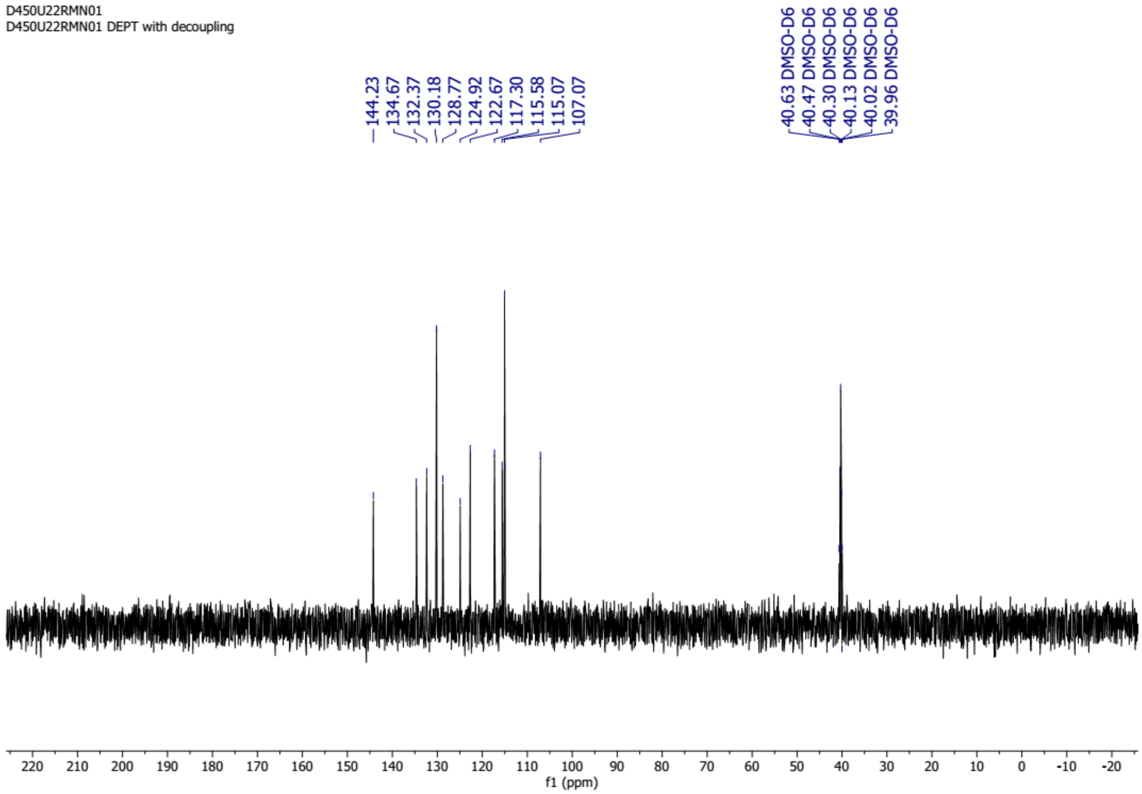


**Figure S43**. $^{13}$C NMR, DEPT 90 spectrum of compound **14b** in DMSO-$d_6$.

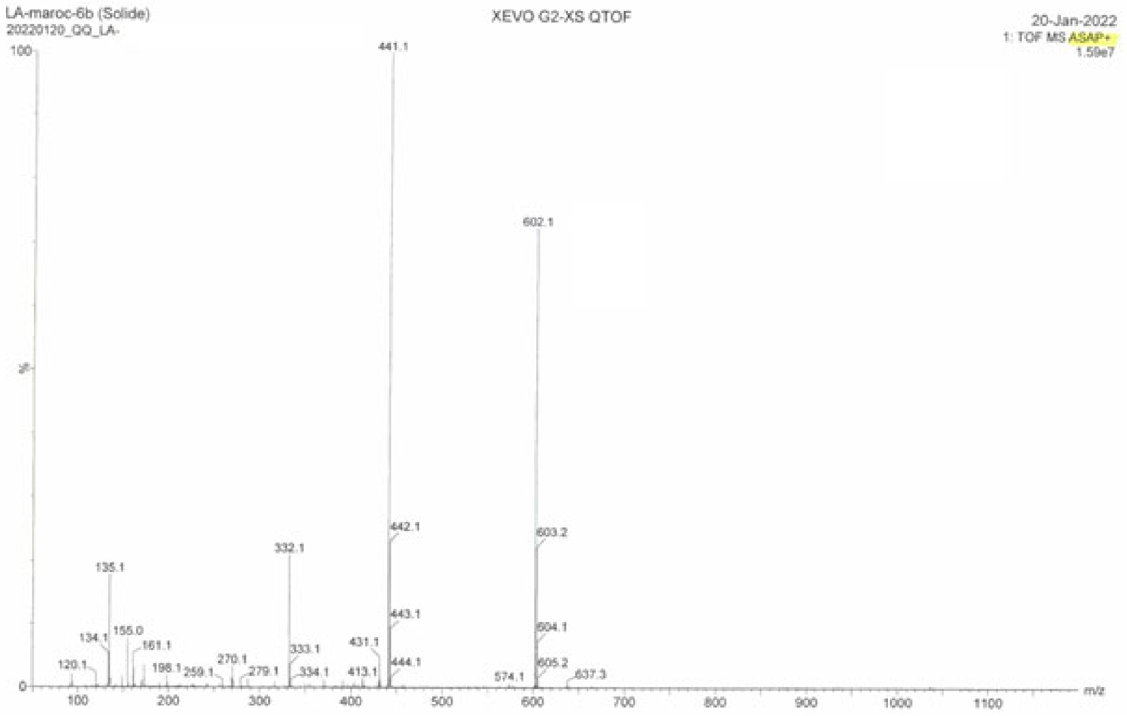


**Figure S44**. MS-ESI(+) spectrum of compound **14b**

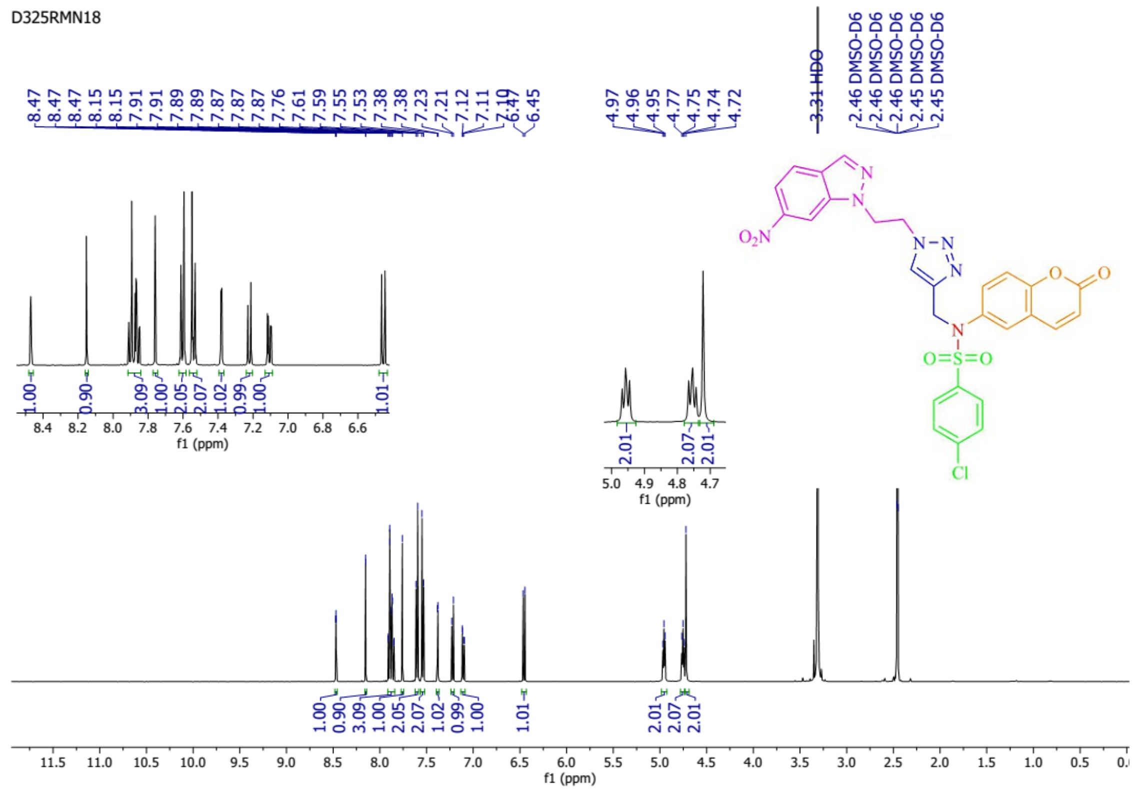


**Figure S45**. $^{1}$H NMR spectrum of compound **14c** in DMSO-$d_6$.

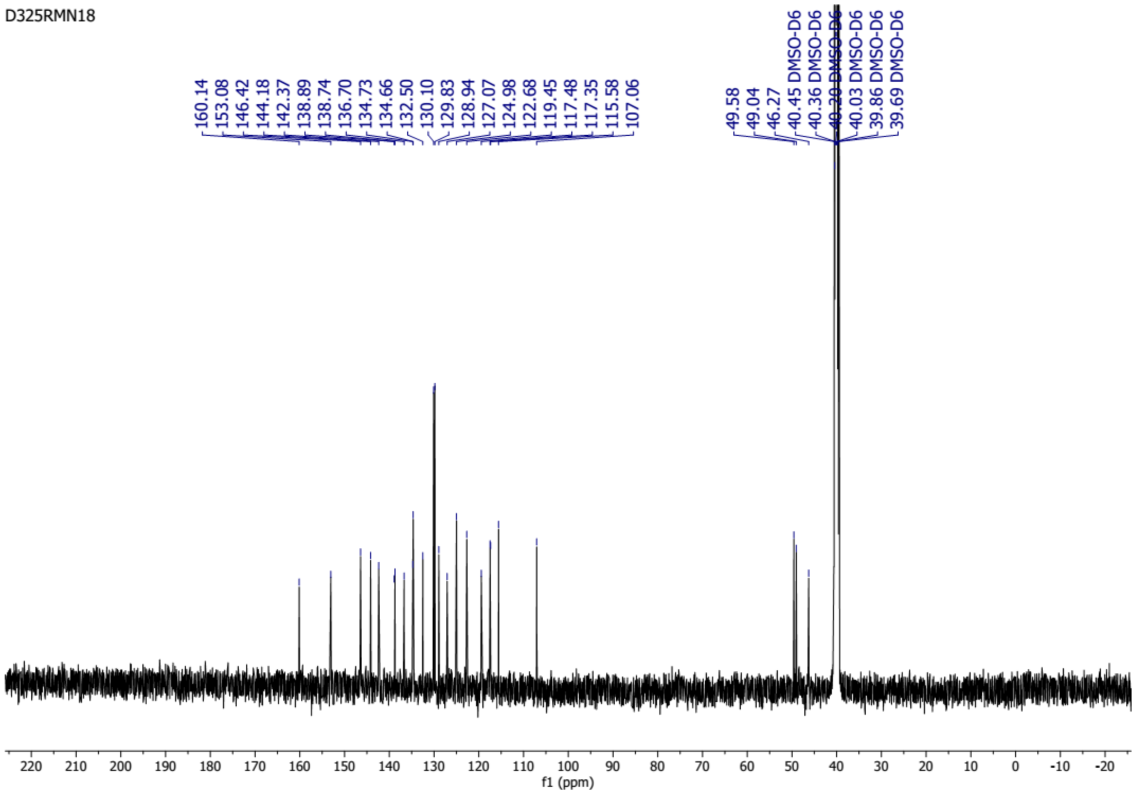


**Figure S46**. $^{13}$C NMR spectrum of compound **14c** in DMSO-$d_6$.

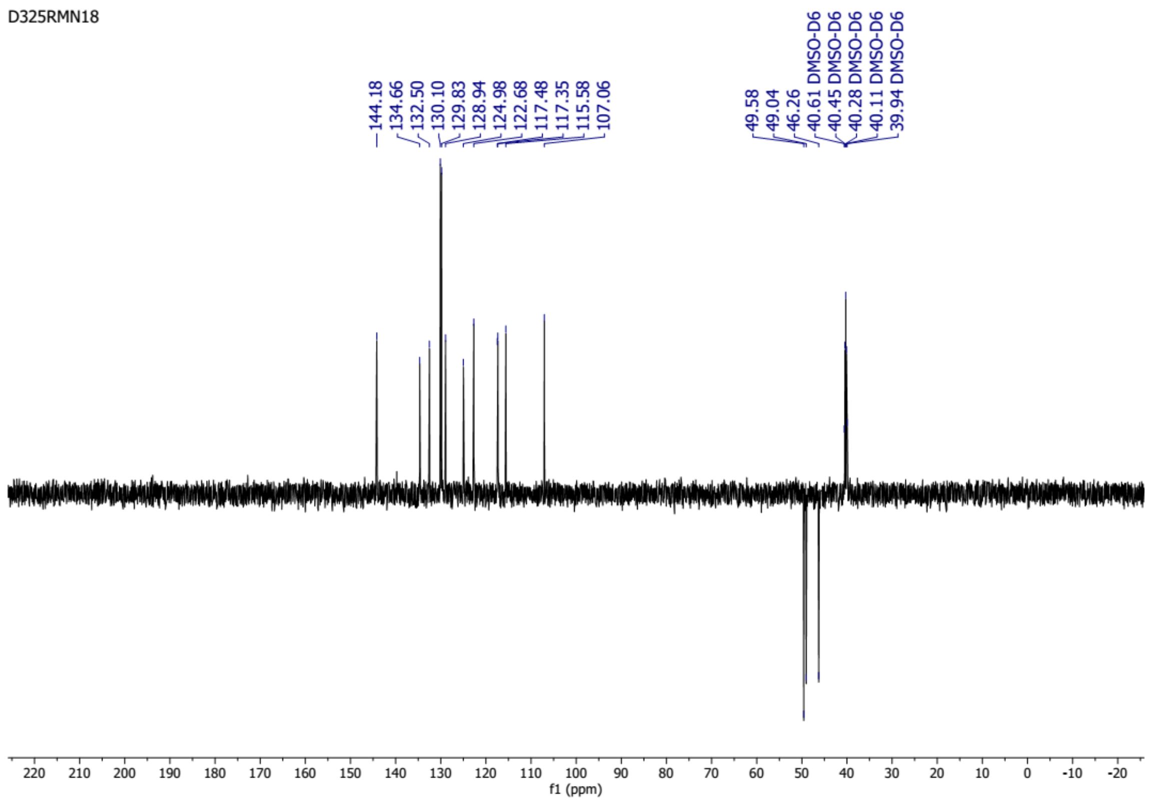


**Figure S47**. $^{13}C$ NMR, DEPT 135 spectrum of compound **14c** in DMSO-$d_6$.

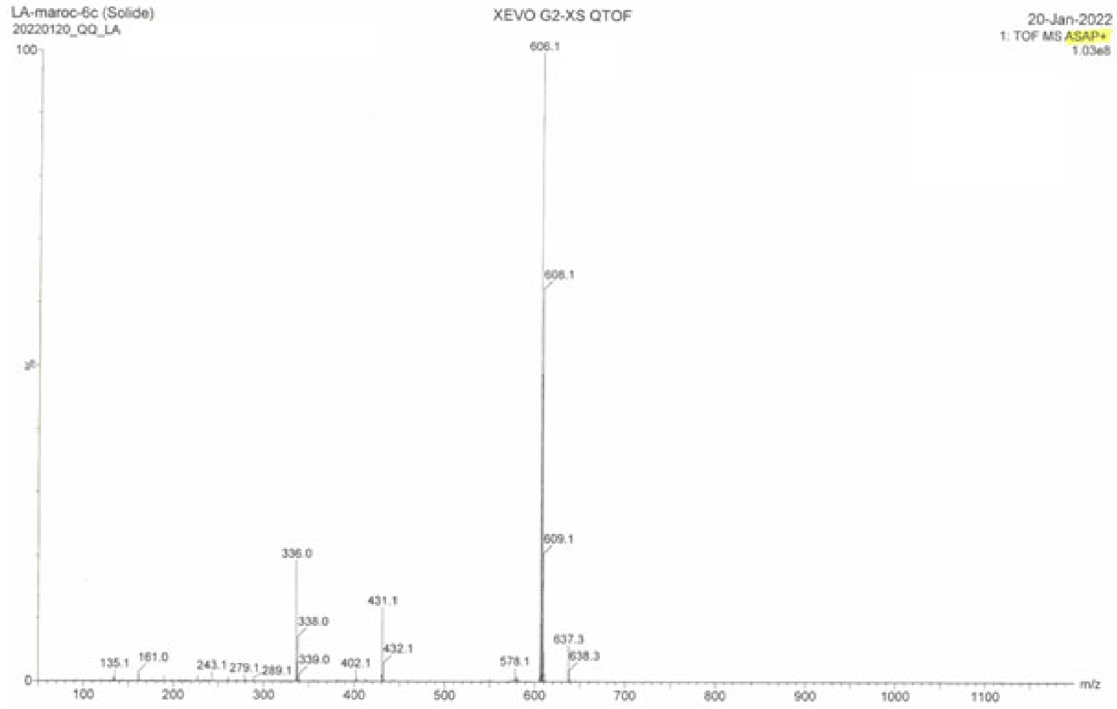


**Figure S48**. MS-ESI(+) spectrum of compound **14c**

## F. Binding map contacts for compound 14a and 14b bound to MAO-A

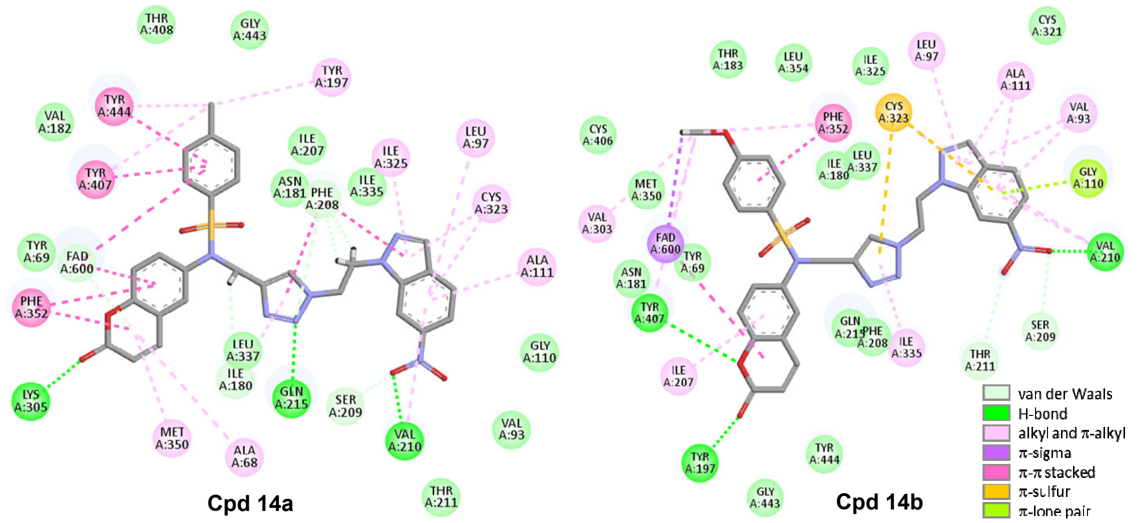


**Fig. S49.** Binding map contacts for compound **14a** and **14b** bound to MAO-A (PDB: 2Z5X). The color code indicated.

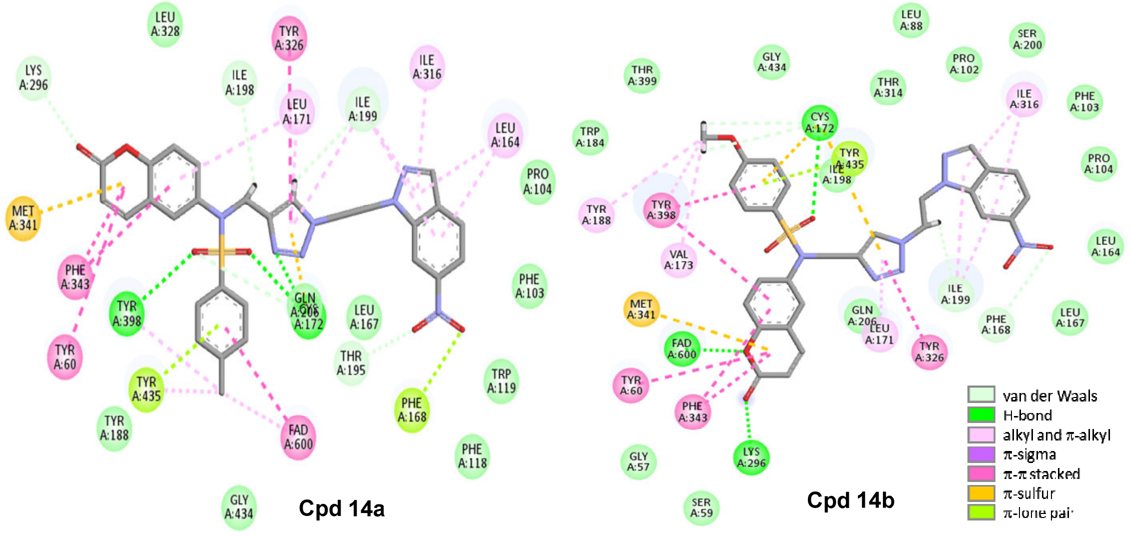


**Fig. S50.** Binding map contacts for compound **14a** and **14b** bound to MAO-B (PDB: 2V5Z). The color code indicated.
